# Catalytic Activity of Ruthenium Complexes Containing Hydrotris(pyrazolyl)methane or Cyclopentadienyl Ligands in the Azide–Alkyne Click Cycloaddition Reaction

**DOI:** 10.1021/acsomega.5c10216

**Published:** 2026-03-18

**Authors:** Belén López-Sánchez, Alberto Gobbo, Gianluca Ciancaleoni, Massimo Guelfi, Fabio Marchetti, Franco Scalambra, Luísa M. D. R. S. Martins, Antonio Romerosa

**Affiliations:** † Área de Química Inorgánica-CIESOL 16721 Universidad de Almería, Almería 04120, Spain; ‡ Department of Chemistry and Industrial Chemistry 9310 University of Pisa, Via Giuseppe Moruzzi 13, Pisa I-56124, Italy; § Centro de Química Estrutural, Departamento de Engenharia Química, Instituto Superior Técnico, Universidade de Lisboa, Av. Rovisco Pais 1, Lisboa 1000-049, Portugal

## Abstract

The Ru­(II) complexes bearing the hydrotris­(pyrazolyl)­methane ligand (Tpm)—([Ru­(κ^3^-*N*-Tpm)­(NCMe)_3_]­(NO_3_)_2_ (**1**), [RuCl­(κ^3^-*N*-Tpm)­(PPh_3_)­(NCMe)]Cl (**2**), [Ru­(κ^3^-*N*-Tpm)­(PPh_3_)­(NCMe)_2_]­(NO_3_)_2_ (**3**), [Ru­(κ^3^-*N*-Tpm)­(κ^2^-*N*-C_5_H_4_NCH_2_NH_2_)­(PPh_3_)]­(NO_3_)_2_ (**4**), [Ru­(κ^3^-*N*-Tpm)­(κ^2^-*N*-NH_2_CH_2_CH_2_NH_2_)­(PPh_3_)]­(NO_3_)_2_ (**5**), and [RuCl­(κ^3^-*N*-Tpm)­(PPh_3_)­(κ^1^-*N*-NH_2_(CH_2_)_2_OH)]Cl (**6**))—were evaluated for the azide/alkyne cycloaddition reaction (“click” reaction) in aqueous and organic media. In parallel, and for comparative purposes, piano-stool–Ru­(II) complexes featuring cyclopentadienyl and adamantane-like ligands, 1,3,5-triaza-7-phosphaadamantane (PTA) and its *N*-monomethylated derivative (mPTA = *N*-methyl-1,3,5-triaza-7-phosphaadamantane) ([RuClCp­(PTA)_2_] (**7**), [RuCp­(OH_2_)­(PTA)_2_]­CF_3_SO_3_ (**8**), [RuClCp­(mPTA)_2_]­(CF_3_SO_3_)_2_ (**9**), and [RuCp­(OH_2_)­(mPTA)_2_]­(CF_3_SO_3_)_3_ (**10**)), were also evaluated for azide–alkyne cycloaddition under similar conditions. Parameters, such as the solvent, substrate concentration, and temperature, were systematically varied to assess their influence on the reaction. The highest conversion rates and selectivity toward the 1,5-disubstituted triazole were achieved in water, acetonitrile/water, and dimethylformamide, while the formation of the 1,4-disubstituted product was slightly favored in methanol. Complexes **3** and **4** exhibited higher regioselectivity and conversion rates in DMF, favoring the formation of the respective 1,5-disubstituted triazole. Complex **3** also catalyzed the cycloaddition of benzyl azide to diphenylacetylene, a representative internal alkyne, reaching a TON of 75 (TOF = 12.5 h^–1^). DFT calculations and NMR and TOF-MS spectroscopic studies were conducted to elucidate the mechanism of catalytic cycloaddition between complex **3**, phenyl azide, and phenylacetylene under the optimized conditions.

## Graphical abstract

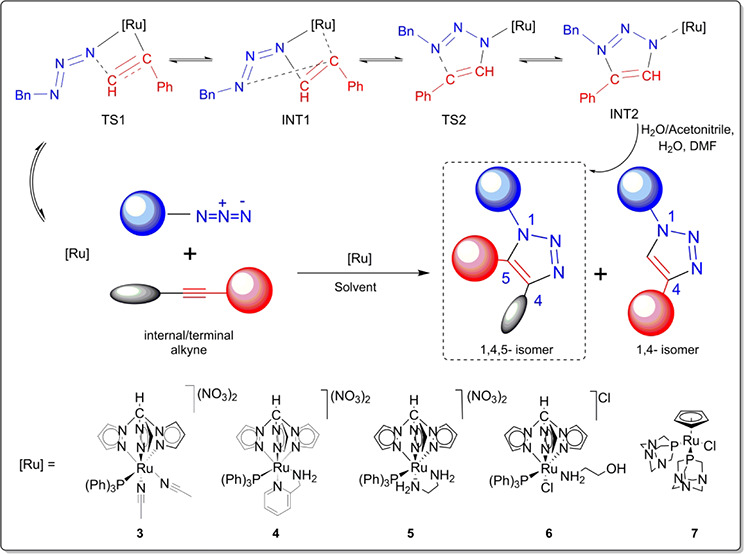

## Introduction

The term “Click chemistry,” introduced by Sharpless and coworkers in 2001,^1^ is a powerful strategy for building versatile products for chemical synthesis, materials science, industrial applications, and pharmaceuticals.^2–7^ This approach affords fast, high-yielding, selective reactions with high atom economy under mild conditions, minimizing purification steps and adhering to green chemistry principles.^8,9^ Click reactions enable access to varied architectures, including linear, grafted, star, branched, and cyclic structures, with the azide–alkyne cycloaddition (AAC) being a prime example. Other notable reactions include thiol–ene, thiol–yne, Diels–Alder, sulfur­(VI)–fluoride exchange, or oxime formation from aldehydes/ketones.^5^

The uncatalyzed AAC, namely the Huisgen reaction, yields mixtures of the 1,4-isomer and 1,5-isomer under high temperatures and extended reaction times (Scheme 1a).^10,11^ The metal-catalyzed versions (MAAC) of this reaction employ a wide variety of transition metals, such as Co, Au, Ag, Ru, Ir, Cu, Ni, Pd, Pt, and Zn.^4,7,12–17^ Copper­(I)-catalyzed AAC (CuAAC) facilitates the regioselective synthesis of 1,4-disubstituted 1,2,3-triazoles under mild conditions, becoming a widely used tool for the synthesis of this family of compounds over the past 22 years (Scheme 1b).^6,14,18–21^ Ruthenium­(II)-catalyzed AAC (RuAAC), introduced by Zhang and coworkers in 2005, offers the regioselective synthesis of 1,5-disubstituted 1,2,3-triazoles, broadening the scope of AAC applications^22^ (Scheme 1c). Despite CuAAC having been more extensively explored, reports on RuAAC for 1,5-disubstituted triazoles are rapidly increasing.^23–30^

**Scheme 1:**
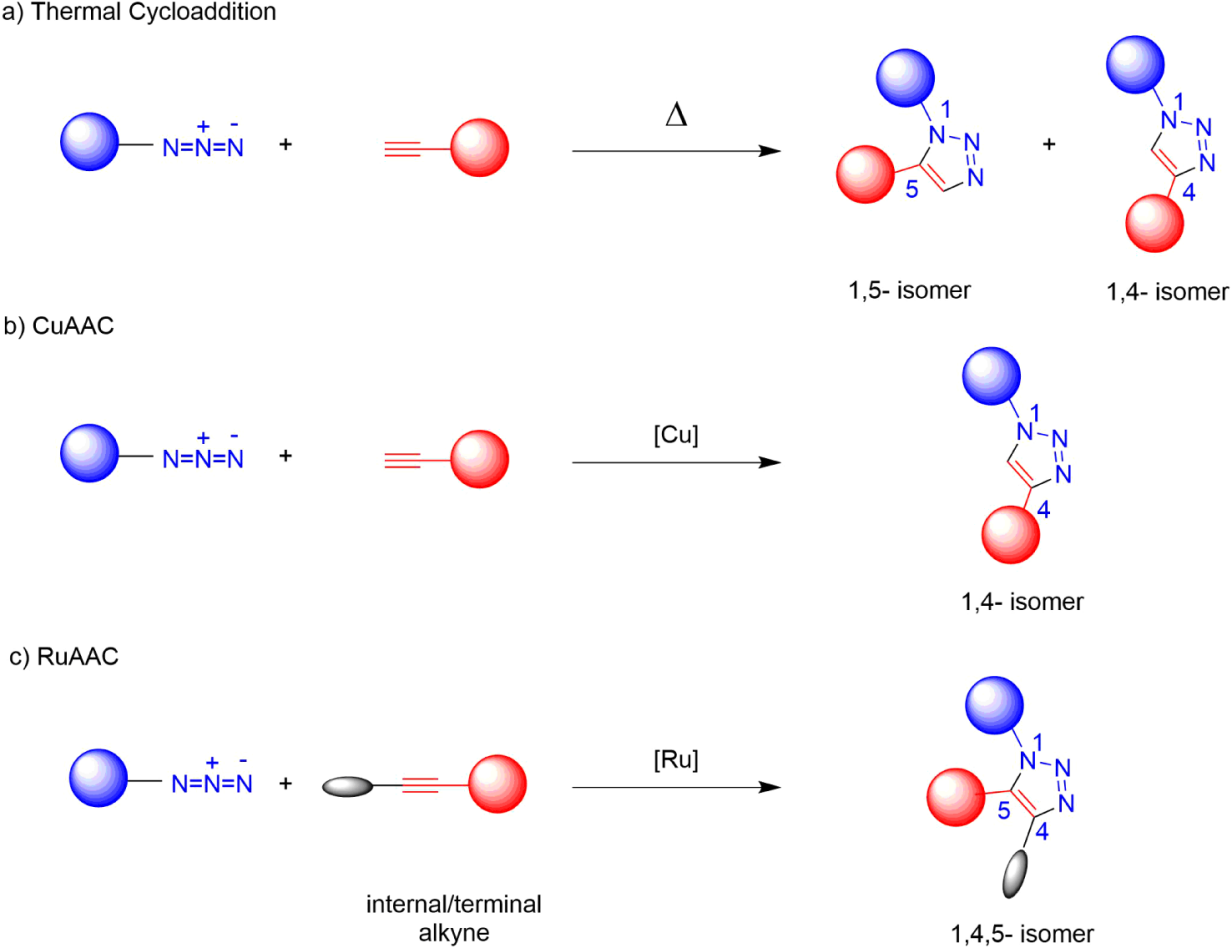
Methodologies Applied for the Synthesis of Triazoles

One of the most studied Ru catalysts for the AAC reaction is the pentamethylcyclopentadienyl (Cp*) ruthenium chloride-based ({RuClCp*}) complex. Unlike CuAAC catalysts, Ru complexes catalyze the regioselective formation of 1,5-disubstituted-1,2,3-triazoles^22,24,31,32^ and also react with internal alkynes to provide trisubstituted 1,2,3-triazoles (Scheme 1c).^22,31–33^ The catalytic activity of the complex [RuCpCl­(PPh_3_)_2_] (2 mol %) shows lower conversions (50% in benzene, <15% in THF) and selectivity (ratio of 1,5-isomer/1,4-isomer = 5.8:1 in benzene, 10:1 in THF) compared to the analogue complex with Cp* (>99%, ratio 1:0 in THF).^22^ This finding suggests that the steric encumbrance imparted by the Cp* ligand positively affects the selectivity for 1,5-disubstituted triazoles.^34^ On the other hand, Fokin’s group reported that the species {RuClCp} with a 1,5-cyclooctadiene (COD) ligand is more active than the analogue {RuClCp*} in cycloaddition reactions of 1-haloalkynes with nitrile oxides and organic azides, yielding mixtures of 4-haloisoxazoles and 5-halotriazoles.^35^ Additionally, the complex [RuClCp*COD] was also found to be active in RuAAC for a wide range of selenoalkyne substrates and azides, giving selenotriazoles in good yields.^36^ Likewise, other families of Ru catalysts have also been explored, such as Ru–Tp complexes (Tp = hydrotris­(pyrazolyl)­borate). The Tp ligand is frequently employed as a substitute for Cp due to its isoelectronic nature and comparable complexation properties.^37^ Examples of this family of complexes are [RuCl­(PPh_3_)_2_(Tp)] and [Ru­(^t^BuNC)­(PPh_3_)­N_3_(Tp)],^38^ which are similar to {RuCp*Cl} complexes, affording both the 1,5-disubstituted and the 1,4,5-trisubstituted 1,2,3-triazoles.^22,31–33,39^ Additionally, other RuAAC catalysts were found active for the synthesis of 1,4-disubstitued like [Ru­(CO)­H_2_(PPh_3_)_3_].^22,40,41^ While a variety of metal complexes (different from Cu) with Tpm (Tpm = hydrotris­(pyrazolyl)­methane), a neutral ligand structurally similar to Tp, have been evaluated for azide–alkyne cycloaddition,^42,43^ ruthenium-based catalysts had not been previously tested for these reactions.

In this work, the new ruthenium­(II) complex containing the Tpm ligand (Tpm = hydrotris­(pyrazolyl)­methane), [Ru­(κ^3^-Tpm)­(NCMe)_3_]­(NO_3_)_2_ (**1**, see Supporting Information, Figure S1–S4), and the other ones [RuCl­(κ^3^-*N*-Tpm)­(PPh_3_)­(NCMe)]Cl (**2**), [Ru­(κ^3^-*N*-Tpm)­(PPh_3_)­(NCMe)_2_]­(NO_3_)_2_ (**3**), [Ru­(κ^3^-*N*-Tpm)­(κ^2^-*N*-C_5_H_4_NCH_2_NH)­(PPh_3_)]­(NO_3_)_2_ (**4**), [Ru­(κ^3^-*N*-Tpm)­(k^2^-*N*-NH_2_CH_2_CH_2_CH_2_NH_2_)­(PPh_3_)]­(NO_3_)_2_ (**5**), and [RuCl­(κ^3^-*N*-Tpm)­(PPh_3_)­(κ^1^-*N*-NH_2_(CH_2_)_2_OH)]Cl (**6**), were tested for the AAC in both aqueous and organic media. Complexes **2**,^44^ **4**, and **5** ^45^ were prepared according to a published procedure. Complexes **3** and **6** ^46^ were synthesized as described in the (Supporting Information Figure S5 and S6). For the sake of comparison, the catalytic performances of the half-sandwich ruthenium­(II) complexes featuring cyclopentadienyl, 1,3,5-triaza-7-phosphaadamantane (PTA), and its *N*-monomethylated derivative (mPTA = *N*-methyl-1,3,5-triaza-7-phosphaadamantane),^47–49^ [RuClCp­(PTA)_2_] (**7**), [RuCp­(OH_2_)­(PTA)_2_]­CF_3_SO_3_ (**8**),^50,51^ ([RuClCp­(mPTA)_2_]­(CF_3_SO_3_)_2_ (**9**),^52^ and [RuCp­(OH_2_)­(mPTA)_2_]­(CF_3_SO_3_)_3_ (**10**)^53,54^ were also evaluated under similar conditions (Figure 1). Finally, a theoretical mechanism for the reaction catalyzed by **3** is proposed, supported by DFT calculations and NMR spectroscopy.

**Figure 1:**
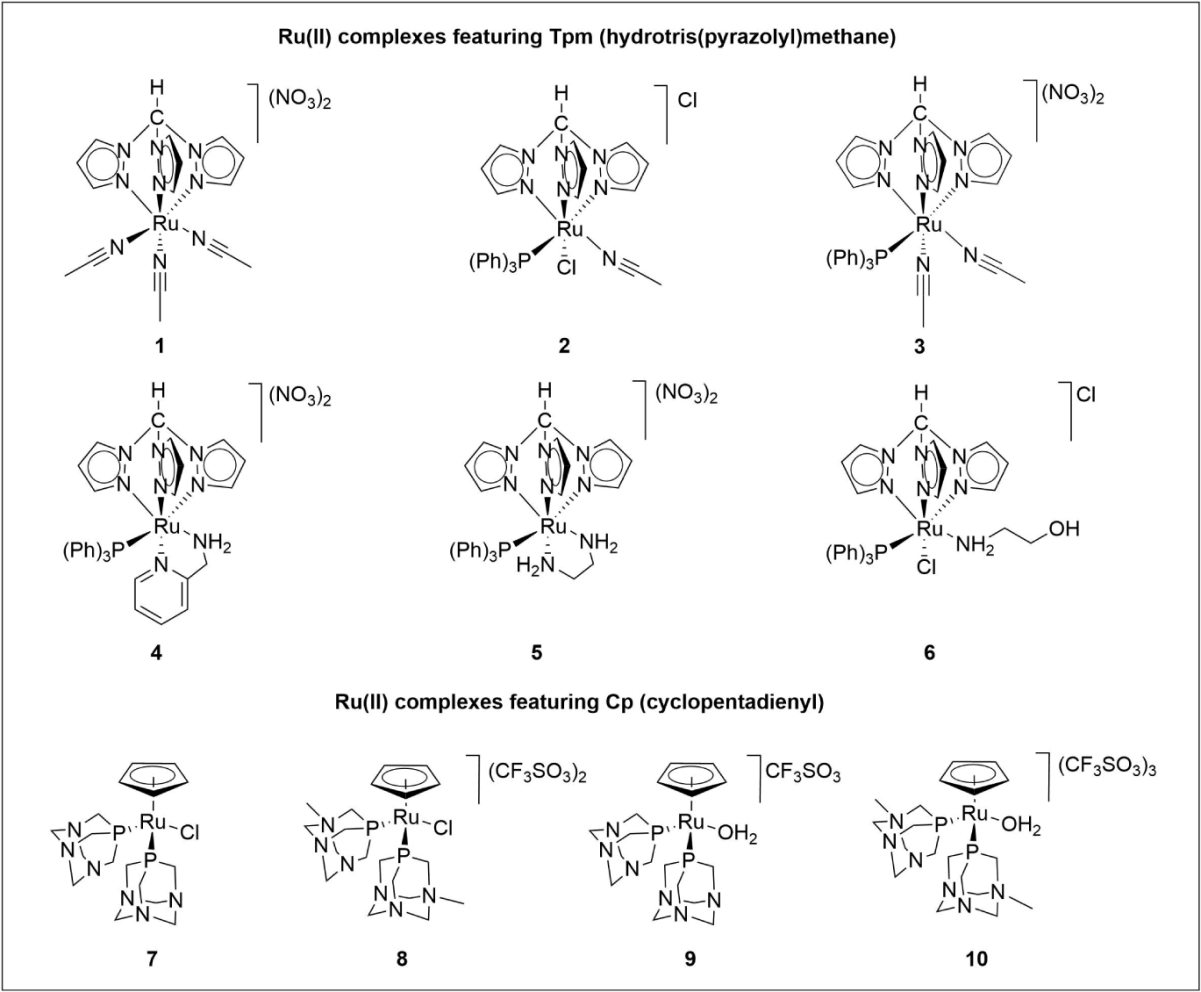
Structure of the complexes evaluated for the azide–alkyne cycloaddition.

## Results and Discussion

### Cycloaddition of Benzyl Azide with Alkynes (Phenyl Acetylene and Diphenylacetylene) Catalyzed by Ruthenium­(II)–C-Scorpionate and Ruthenium­(II) −Piano-Stool Complexes

The catalytic activities of complexes **1**–**6**, containing Tpm, and the hydrosoluble ruthenium cyclopentadienyl complexes **7**–**10** were evaluated for the cycloaddition reaction of benzyl bromide (**I’**), NaN_3_ (**I’’**), and phenylacetylene (**II**) to form the 1,5-disubstituted triazole 1-benzyl-5-phenyl-1H-1,2,3-triazole (**III**) and the 1,4-disubstituted triazole 1-benzyl-4-phenyl-1H-1,2,3-triazole (**IV**) (Scheme 2). The formation of benzyl azide (**I**) occurs *in situ* through the substitution reaction of **I’** with NaN_3_ at room temperature in a single step.^55^ Consistent with multicomponent reactions,^56–58^ numerous catalytic systems reported in the literature achieve the generation of **I** *in situ*.^8,15,29,30,59–67^ This strategy provides excellent atom economy and avoids the purification steps necessary to isolate the organic azide. Nevertheless, the reaction between benzyl bromide and NaN_3_ is not immediate, and competitive reactions could happen with this reactant in the presence of the catalyst, giving rise to different products.

**Scheme 2:**
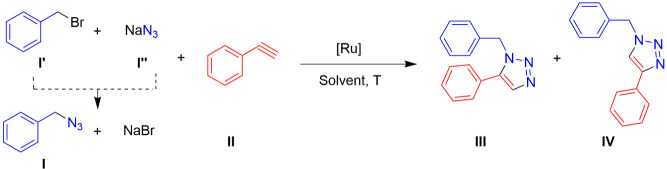
Catalytic Cycloaddition of Benzyl Bromide (**I’**), NaN_3_ (**I’’**), and Phenylacetylene (**II**) into 1-Benzyl-5-phenyl-1H-1,2,3-triazole (**III**) and 1-Benzyl-4-phenyl-1H-1,2,3-triazole (**IV**) Mediated by Ruthenium­(II) Complexes in Several Solvents

All cycloaddition reactions with benzyl bromide (**I’**), phenylacetylene (**II**), and NaN_3_ (**I’’**) were conducted with an **I’/I’’/II** ratio of 1:1:1.25, keeping a substrate concentration of 0.1 M for **I**’ and **I’’**, and 0.14 M for **II**. Typical reactions were catalyzed by 1 mol % of Ru­(II) complexes with respect to **I’** and **I’’**, corresponding to a catalyst concentration of 0.001 M. Solvents in which the complexes were sufficiently soluble to achieve this concentration were evaluated. So, complexes **2–6** were dissolved at a 0.001 M concentration in water, methanol, water/acetonitrile 1:1, dichloromethane (DCM), and dimethylformamide (DMF), while **1** was not sufficiently soluble in DCM (Table S1). In contrast, complexes **7–10** were adequately soluble in water and DMF, poorly soluble in methanol, and insoluble in acetonitrile and DCM (Table S2). Therefore, initial catalytic tests were carried out in common solvents for all complexes: methanol and water as representative examples of polar protic solvents, and DMF as a representative polar aprotic solvent. All experiments were performed under inert conditions for up to 6 h. Products were extracted with ethyl acetate, and conversions were obtained by ^1^H NMR. In all reactions, product conversions were calculated relative to theoretical benzyl azide (**I**) and the products **III** and **IV**. Benzyl bromide (**I’**) was not detected in any of the reactions under the tested conditions.

In methanol at 65 °C, the C-scorpionate–Ru­(II) catalysts **1**–**6** achieved a high substrate transformation into **III** and **IV** (conversion >99%), except for complex **1,** which only reached 32% conversion. Similar proportions of isomers **III** and **IV** were observed when **1**, **2,** and **3** were employed. However, complexes **4**–**6** gave rise to a slight increase in the level of formation of **IV**. The highest achieved **III**/**IV** ratio was 1:1.2 with complex **5** in MeOH. On the other hand, the Cp–Ru­(II) complexes containing PTA exhibited higher catalytic efficiency (**7**: 99%, **9**: 94%) than those with mPTA (**8**: 89%, **10**: 82%). Furthermore, complexes **7** and **8**, which contain a chloride ligand coordinated to the metal, exhibited higher catalytic conversions than their analogues with water, complexes **9** and **10**. Complex **7** was the most active (99%) with a **III/IV** ratio of 1:1.1, which is a similar isomer ratio to that obtained by Ru–C-scorpionate complexes (Table 1).

**Table 1:**
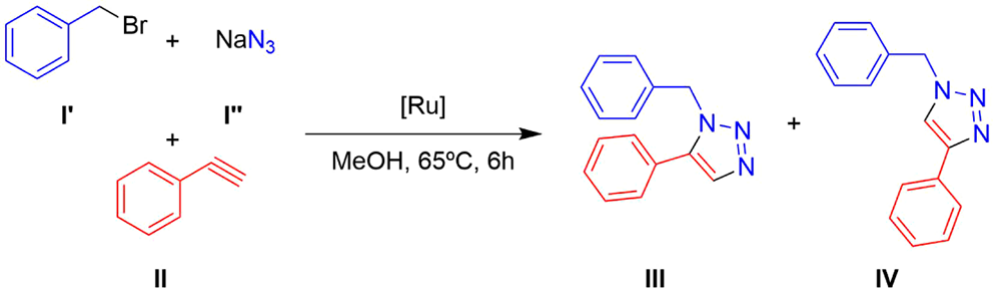
Catalytic Cycloaddition of Benzyl Bromide (**I’**), Phenylacetylene (**II**), and NaN_3_ (**I’’**) into 1-Benzyl-4phenyl-1H-1,2,3-triazole (**III**) and 1-Benzyl-4-phenyl-1H-1,2,3-triazole (**IV**) Mediated by Ruthenium­(II) Complexes in MeOH at 65 °C Under Inert Conditions^a^

Catalyst	III (%)^b^	IV (%)^b^	Conversion (%)^b^	Ratio III/IV
[Ru(κ^3^-*N*-Tpm)(NCMe)_3_](NO_3_)_2_ (**1**)	16	16	32	1:1
[RuCl(κ^3^-*N*-Tpm)(PPh_3_)(NCMe)]Cl (**2**)	49	49	98	1:1
[Ru(κ^3^-*N*-Tpm)(PPh_3_)(NCMe)_2_](NO_3_)_2_ (**3**)	48	52	99	1:1.1
[Ru(κ^3^-*N*-Tpm)(κ^2^ *-N*-C_5_H_4_NCH_2_NH)(PPh_3_)](NO_3_)_2_ (**4**)	48	52	99	1:1.1
[Ru(κ^3^-*N*-Tpm) (κ^2^ *-N*-NH_2_CH_2_CH_2_CH_2_NH_2_)(PPh_3_)](NO_3_)_2_ (**5**)	46	54	99	1:1.2
[RuCl(κ^3^-Tpm)(PPh_3_)(κ^1^-*N*-NH_2_(CH_2_)_2_OH)]Cl (**6**)	48	52	99	1:1.1
[RuClCp(PTA)_2_] (**7**)	47	53	99	1:1.1
[RuClCp(mPTA)_2_](CF_3_SO_3_)_2_ (**8**)	43	46	89	1:1.1
[RuCp(OH_2_)(PTA)_2_]CF_3_SO_3_ (**9**)	44	50	94	1:1.1
[RuCp(OH_2_)(mPTA)_2_](CF_3_SO_3_)_3_ (**10**)	39	43	82	1:1.1

a 1 mol % [Ru], 6 h, benzyl azide (**I**) was formed *in situ* by the reaction of phenyl bromide (**I’**) and NaN_3_ (**I’’**) in a ratio 1:1, MeOH, 65 °C, **I**/**II** = 1:1.25 (**II** = phenylacetylene), [**I**] = 0.11 M, [**II**] = 0.14 M.

b Conversion (%) obtained by ^1^H NMR.

Performance tests conducted with both cyclopentadienyl and C-scorpionate–ruthenium­(II) complexes in water and DMF at 100 °C also gave a mixture of **III** and **IV**, with **III** being predominantly obtained. In aqueous media, among the evaluated Ru­(II)–C-scorpionate complexes, only **1**, **2**, **5,** and **6** exhibited catalytic activity, achieving conversions of no more than 75%, with a **III/IV** ratio of 1.1:1, while all Ru–Cp complexes exhibited high catalytic activity, with conversions exceeding 80%. The most effective catalysts were those bearing the PTA ligand, **7** being the most active (conversion = 98%, **III/IV** = 1.1:1) (Table 2). The use of a polar aprotic solvent, such as DMF, provided higher conversion rates, with nearly all of the Ru–C-scorpionate complexes **2**–**6** tested exceeding 90% conversion but demonstrating enhanced selectivity toward the 1,5-disubstituted triazole **III**, except for complex **1** that exhibits a lower conversion (approximately 75%). Formation of **III** was achieved with rates ranging from 50% to 60%, and TOF values of 10 h^–1^. The highest TOF values for the synthesis of **III** were obtained with complexes **5** (TOF = 9.8 h^–1^, 56% conv.), **4** (TOF = 9.3 h^–1^, 59% conv.), and **6** (TOF = 9.7 h^–1^, 58% conv.), with **III**/**IV** ratios of 1.5:1, 1.6:1, and 1.7:1, respectively. On the other hand, for Ru–Cp complexes, the best catalyst in DMF was **7**, which achieved a 92% conversion with a ratio of **III**/**IV** of 1.2:1. All other Ru complexes yielded lower conversions (**8**: 82%, **9**: 65%, **10**: 65%) and showed slightly better selectivity for the formation of the 1,5-isomer, with ratios ranging from 1.7:1 to 1.8:1. In general, in DMF, Ru–Tpm complexes demonstrated superior conversions and selectivity compared to Ru–Cp complexes for the formation of 1,5-disubstituted triazole **III** (Table 2).

**Table 2:**
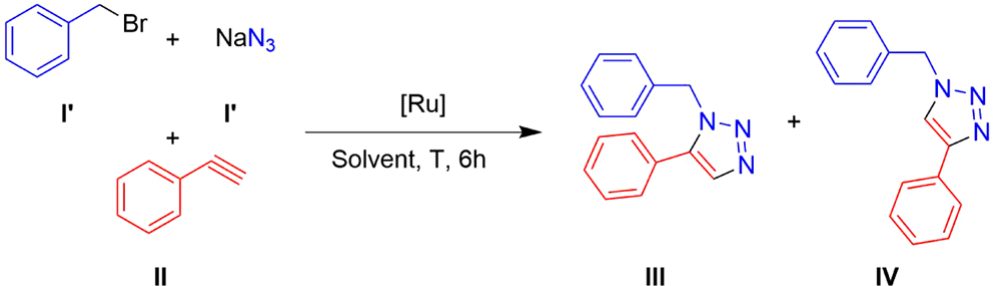
Catalytic Cycloaddition of Benzyl Bromide (**I’**), Phenylacetylene (**II**), and NaN_3_ (**I’’**) into 1-Benzyl-5-phenyl-1H-1,2,3-triazole (**III**) and 1-Benzyl-4-phenyl-1H-1,2,3-triazole (**IV**) Mediated by Ruthenium­(II) Complexes in DMF and H_2_O at 100 °C Under Inert Conditions^a^

	Conversion (%)^b^ in DMF			Conversion (%)^b^ in H_2_O		
Catalyst	III	IV	Ratio III/IV in DMF	III	IV	Ratio III/IV in H_2_O
[Ru(κ^3^-*N*-Tpm)(NCMe)_3_](NO_3_)_2_ (**1**)	49	28	1.8:1	33	24	1.4:1
[RuCl(κ^3^‑*N*-Tpm)(PPh_3_)(NCMe)]Cl (**2**)	52	44	1.2:1	29	27	1.1:1
[Ru(κ^3^-*N*-Tpm)(PPh_3_)(NCMe)_2_](NO_3_)_2_ (**3**)	55	39	1.4:1	–	–	–
[Ru(κ^3^-*N*-Tpm)(κ^2^‑*N*-C_5_H_4_NCH_2_NH)(PPh_3_)](NO_3_)_2_ (**4**)	59	38	1.6:1	30	30	1:1
[Ru(κ^3^-*N*-Tpm)(κ-^2^‑*N*-H_2_N CH_2_CH_2_NH_2_)(PPh_3_)](NO_3_)_2_ (**5**)	56	37	1.5:1	39	35	1.1:1
[RuCl(κ ^3^‑*N*-Tpm)(PPh_3_)(κ^1^ *N*-NH_2_(CH_2_)_2_OH)]Cl (**6**)	58	34	1.7:1	–	–	–
[RuClCp(PTA)_2_] (**7**)	51	41	1.2:1	52	46	1.1:1
[RuClCp(mPTA)_2_](CF_3_SO_3_)_2_ (**8**)	51	31	1.7:1	48	48	1:1
[RuCp(OH_2_)(PTA)_2_]CF_3_SO_3_ (**9**)	41	24	1.7:1	43	43	1:1
[RuCp(OH_2_)(mPTA)_2_](CF_3_SO_3_)_3_ (**10**)	23	42	1.8:1	39	36	1.1:1

a 1 mol % [Ru], 6 h, benzyl azide (**I**) was formed *in situ* by the reaction of phenyl bromide (**I’**) and NaN_3_ (**I’’**) in a ratio 1:1, DMF, H_2_O, 100 °C, **I**/**II** = 1:1.25 (**II** = phenylacetylene), [**I**] = 0.11 M, [**II**] = 0.14 M/

b conversion (%) obtained by ^1^H NMR.

Next, experiments were performed to evaluate the effects of other solvents and decreasing the reaction temperature on the conversion and selectivity of the product. Given that the choice of solvent significantly influences the reaction outcome, determining which compound, **III** or **IV**, is favored mainly. The study aimed to achieve an optimal balance between conversion and selectivity for the synthesis of 1,5-disubstituted triazole (**III**), as Ru complexes of similar structure reported in the literature have shown the ability to catalyze the regioselective formation of 1,5-disubstituted-1,2,3-triazoles.^23–27^

Complexes **2**–**6** were evaluated in a 1:1 mixture of water and acetonitrile at 85 °C. This solvent mixture was selected to optimize both solubility and reaction conditions. Under these conditions, complexes **2**–**6** showed improved conversions compared with the reactions performed in pure water. The formation of isomer **III** was slightly enhanced, with **III**/**IV** ratios ranging from 1.1:1 to 1.2:1. The highest conversions into **III** were 46% and 51%, which were obtained with complexes **5** and **6**, respectively. This solvent mixture was also used for reactions catalyzed by Ru–Cp complexes **7**–**10**. Their high solubility in water, combined with their insolubility in organic solvents, enables their potential recycling in the cycloaddition of **I** and **II** in water/acetonitrile 1:1. Although similar conversions to those in water were found using **8**–**10**, a slightly better selectivity and conversion into **III** were achieved with complex **7** (97%, ratio **III**/**IV** 1.4:1). After determining the time required to complete the cycloaddition of **I** and **II** catalyzed by **7** in water/acetonitrile 1:1 (6 h), the products were extracted with ethyl acetate, and the collected organic layers were dried over MgSO_4_, and vacuum-dried. Then, the solution with compound **7** in water was recycled for a second run. This second cycle yielded **III** + **IV** with a conversion of 78% (**III**/**IV** = 1.2:1). The total TON for **III** and **IV** after two cycles was 175 (TOF = 15 h^–1^), with the TON value for **III** being slightly higher (TON = 99, TOF = 8.25 h^–1^) than for **IV** (TON = 76, TOF = 6.33 h^–1^). Therefore, all Ru–Cp complexes are more active in water/acetonitrile than in pure water and slightly enhance the selectivity for the formation of 1,5-disubstituted triazole (**III**). However, for the Ru–Tpm complexes, DMF exhibited better performance in both conversion and selectivity (Table 3).

**Table 3:**
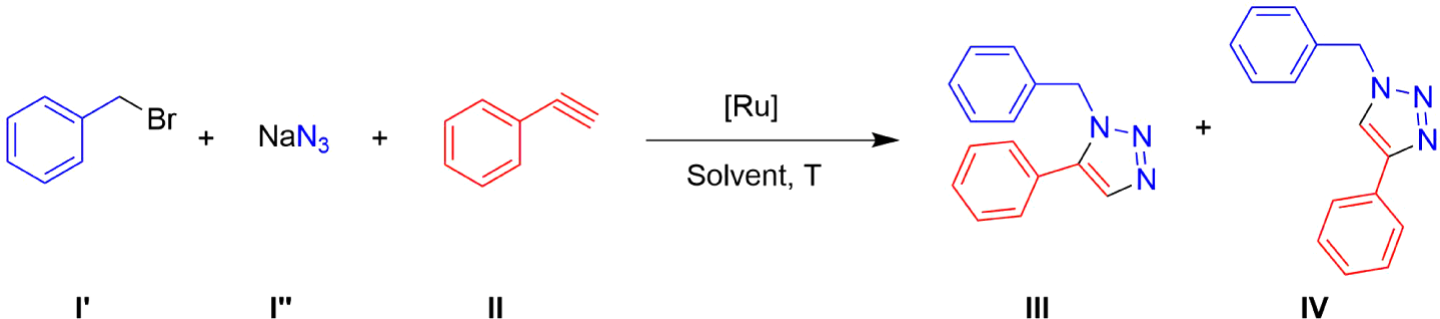
Catalytic Cycloaddition of Benzyl Bromide (**I’**), Phenylacetylene (**II**), and NaN_3_ (**I’’**) into 1-Benzyl-5phenyl-1H-1,2,3-triazole (**III**) and 1-Benzyl-4phenyl-1H-1,2,3-triazole (**IV**) Mediated by Selected Ruthenium­(II) Complexes in Several Solvents^a^

			Conversion (%)			
Catalyst	Solvent	*T* (°C)	III	IV	Ratio III/IV	Total Conversion^b^ (%)
[RuCl(κ^3^-*N*-Tpm)(PPh_3_)(NCMe)]Cl (**2**)	H_2_O	100	29	27	1.1:1	56
	H_2_O/CH_3_CN	85	54	46	1.2:1	99
	DMF	100	52	44	1.2:1	96
[Ru(κ^3^-N-Tpm)(PPh_3_)(NCMe)_2_](NO_3_)_2_ (**3**)	H_2_O	100	–	–	–	–
	H_2_O/CH_3_CN	85	51	49	1.04:1	99
	DMF	100	55	39	1.4:1	94
[Ru(κ^3^-*N*-Tpm)(κ^2^-*N*-C_5_H_4_NCH_2_NH)(PPh_3_)](NO_3_)_2_ (**4**)	H_2_O	100	30	30	1:1	60
	H_2_O/CH_3_CN	85	39	34	1.2:1	73
	DMF	100	59	38	1.6:1	96
[Ru(κ^3^-*N*-Tpm)(κ^2^-*N*-H_2_N CH_2_CH_2_NH_2_)(PPh_3_)](NO_3_)_2_ (**5**)	H_2_O	100	39	35	1.1:1	74
	H_2_O/CH_3_CN	85	46	44	1.2:1	90
	DMF	100	56	37	1.5:1	93
[RuCl(κ^3^-*N*-Tpm)(PPh_3_)(κ^1^-*N*-NH_2_(CH_2_)_2_OH)]Cl (**6**)	H_2_O	100	–	–	–	–
	H_2_O/CH_3_CN	85	51	44	1.2:1	95
	DMF	100	58	34	1.6:1	92
[RuClCp(PTA)_2_] (**7**)	H_2_O	100	52	46	1.1:1	98
	H_2_O/CH_3_CN	85	56	41	1.4:1	97
	DMF	100	51	41	1.2:1	92
[RuClCp(mPTA)_2_](CF_3_SO_3_)_2_ (**8**)	H_2_O	100	48	48	1:1	96
	H_2_O/CH_3_CN	85	49	47	1.1:1	95
	DMF	100	51	31	1.7:1	82

a 1 mol % [Ru], 6 h, proportion of H_2_O/acetonitrile 1:1, benzyl azide (**I**) was formed *in situ* by the reaction of benzyl bromide (**I’**) and NaN_3_ (**I’’**) in a ratio 1:1, **I**/**II** = 1:1.25 (**II** = phenylacetylene), [**I**] = 0.11 M, [**II**] = 0.14 M.

b Conversion (%) obtained by ^1^H NMR.

Reactions with complexes **3**–**6** in DMF and with **7** in acetonitrile/water were studied over time. Ru–C-scorpionate complexes catalyzed conversions exceeding 90% within 6 h (Figure 2a and 2b, Figure S7–S11). Complexes **4** and **5** exhibited the fastest catalytic activities, achieving a conversion of 69% (**4**: 32% for **III**; 37% for **IV**) and 64% (**5**: 37% for **III**; 27% for **IV; III**/**IV** = 1.4:1) in 1 h, and after 6 h, 96% conversion in those reactions catalyzed by **4** (59% of **III**; 38% of **IV**; **III**/**IV** = 1.6:1) and 93% substrate transformation in reactions catalyzed by **5** (59% of **III**; 33% of **IV**; **III**/**IV** = 1.8:1). Nevertheless, the Ru–Cp complex **7** was faster, giving rise to a conversion of 71% in only 1 h (TON = 71, TOF = 71 h^–1^), with a better isomerization ratio in favor of **IV** (27% of **III**; 44% of **IV**; **III**/**IV**: 1.6:1). But at the end of the reaction, the ratio between isomers was different (41% of **IV;** 56% of **III**; **III**/**IV**: 1.4:1). Given that the formation of benzyl azide (**I**) from benzyl bromide (**I’**) and NaN_3_ (**I’’**) occurs *in situ*, and benzyl bromide (**I’**) was not detected at any time, the rate-limiting step is considered to be the cycloaddition reaction between **I** and **II** to give **III** and **IV**. To determine a plausible kinetic model, the conversion data were compared to zero-, first-, and second-order rate laws. The zero- and first-order linear plots yielded poor fits. On the other hand, the fitting to a second-order rate law provided the best linear fit for the reactions catalyzed by the different complexes. This specific model, represented by the equation: $K_{\mathrm{obs}}\cdot \left(\left[\mathrm{II}\right]i-\left[I\right]i\right)\cdot T=\ln\left(\frac{\left[II\right]t\cdot \left[I\right]i}{\left[I\right]t\cdot \left[II\right]i}\right)$ provided the best fit across the entire series of reactions (Figure S12). The analysis of the apparent second-order rate constant (*K*_obs_) revealed a trend in reactivity, with the fastest catalyst being complex **7** with the highest rate constant (*K*_obs_ = 0.147 M^–1^·min^–1^), closely followed by complexes **4** and **5** (*K*_obs_ = 0.143 and 0.127 M^–1^·min^–1^, respectively). Additionally, the individual rate constants (*K*_**III**_ and *K*_**IV**_) confirm that the formation of *K*_**III**_ is kinetically favored consistently across all complexes (Figure 2c).

**Figure 2:**
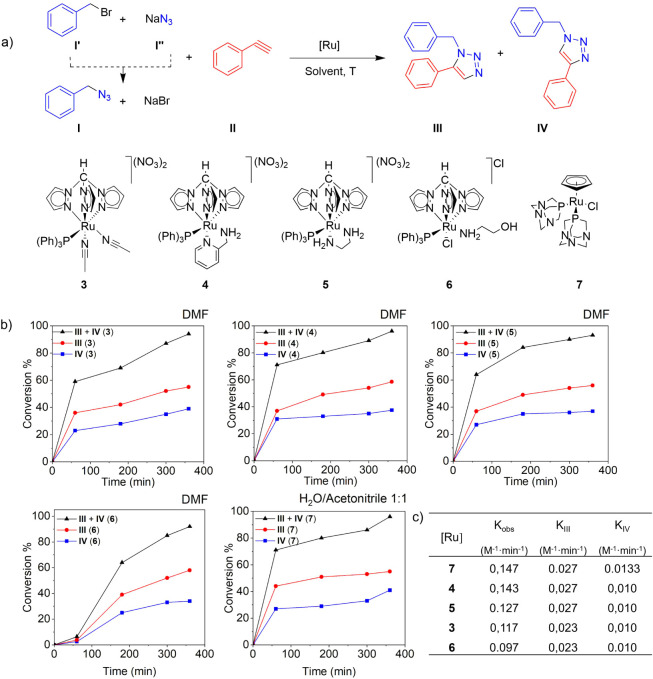
(a) Catalytic cycloaddition of benzyl bromide (**I’**), phenylacetylene (**II**), and NaN_3_ (**I’’**) into 1-benzyl-5-phenyl-1H-1,2,3-triazole (**III**) and 1-benzyl-4-phenyl-1H-1,2,3-triazole (**IV**) mediated by **3**–**7**, in DMF (**3**–**6**) and water/acetonitrile 1:1 (**7**). b) Dependence of conversion vs. time, in DMF (**3**–**6**) and water/acetonitrile 1:1 (**7**). c) Rate determination for a second-order catalytic cycloaddition reaction (*K*_obs_) and individual rate constants (*K* **_III_** and *K* **_IV_**). Conditions: 1 mol % [Ru], DMF (100 °C), and H_2_O/acetonitrile 1:1 (85 °C). **I** was formed *in situ* by the reaction of **I’** and **II’’** in a ratio 1:1, **I/II** = 1:1.25, [**I**] = 0.11 M, [**II**] = 0.14 M. Conversion (%) obtained by NMR, as an average of three experiments.

To understand why complexes containing a C-scorpionate ligand enhance the regioselectivity for the synthesis of 1,5-disubstituted triazole (**III)** in polar aprotic solvents and under lower reaction temperatures, reactions with complexes **2**–**6** in dichloromethane (DCM), which is less polar than DMF and acetonitrile, were carried out at 40 °C. Nonetheless, very low conversions were observed when complexes **2** (21%), **3** (23%), and **4** (4%) were used, and although higher substrate conversions were obtained using **5** and **6** (>90% conv), most of the products were byproducts, with low selectivity toward isomers **III** (**5**: 33%, **6**: 15%) and **IV** (**5**: 20%, **6**: 7%) (Table S1).

The last catalytic tests investigated the cycloaddition of **I’** and **I’’** and **II** catalyzed by **3**–**6**. The experiments were carried out under identical conditions to those discussed previously, but with increased concentrations of the azide and alkyne substrates to 0.55 M (**I**) and 0.70 M (**II**) in DMF and MeOH. With complexes **4**, **5**, and **6** in DMF, a higher substrate concentration did not significantly improve conversion and regioselectivity. Nevertheless, by using complex **3,** the selectivity for isomer **III** was markedly improved, achieving a total conversion of 89% with a **III**/**IV** ratio of 2.4:1 (Figure S13; more details in Table S3). In contrast, a higher concentration of substrates in MeOH resulted in less substrate transformation and lower selectivity, as shown by the observed **III**/**IV** ratios of 1:1 using complexes **3** and **4**, and 1:1.1 using complexes **5** and **6** (Table S3 and Figure S14).

To gain mechanistic insights, the reaction with **3** and **4** in DMF was monitored by ^1^H NMR and ^31^P­{^1^H} NMR spectroscopy under the best conversion conditions. Surprisingly, in 5 mm NMR tubes without stirring, the selectivity improved. Complex **3** catalyzed the cycloaddition to achieve 76% selectivity for **III** (TOF **=** 12.7 h^–1^; **III**/**IV** ratio = 4.5:1), and complex **4** achieved 72% selectivity for **III** (TOF **=** 12 h^–1^; **III**/**IV** ratio = 3.6:1) (Scheme 3).

**Scheme 3:**
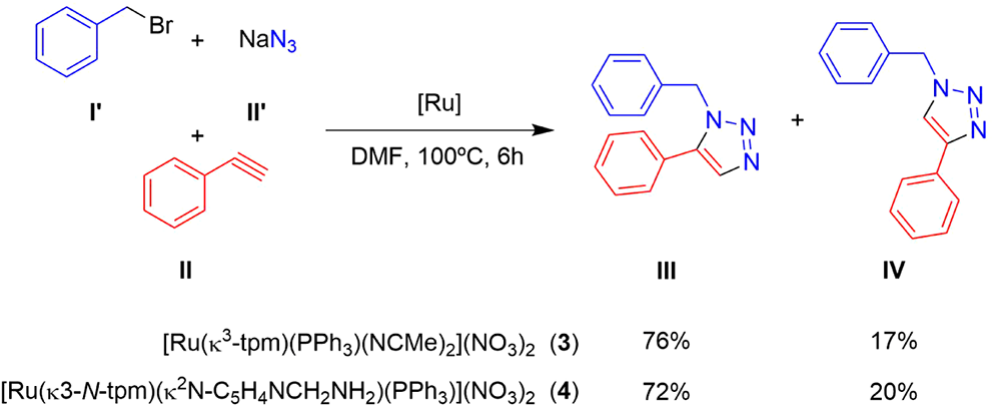
Catalytic Cycloaddition of Benzyl Bromide (**I’**), Phenylacetylene (**II**), and NaN_3_ (**I’’**) into 1-Benzyl-5-phenyl-1H-1,2,3-triazole (**III**) and 1-Benzyl-4-phenyl-1H-1,2,3-triazole (**IV**) Mediated by **3** and **4** Depending on the Concentration of **I** and **II** in DMF[Fn sch3-fn1]

i Conditions: 1 mol % [Ru], DMF, **I** was formed *in situ* by the reaction of **I’** and **II’’** in a ratio 1:1, **I/II** = 1:1.25, **I** = 0.55 M, and **II** = 0.70 M; conversion (%) was obtained by NMR, as an average of three experiments.

Complex **3**, the most selective catalyst, was further evaluated for the cycloaddition of the symmetric internal alkyne diphenylacetylene (**V**), which was transformed into 1-benzyl-4,5-diphenyl-1*H*-1,2,3-triazole (**VI**) (TON = 75; TOF = 12.5 h^–1^) with a conversion of 72% after 6 h in DMF (Scheme 4). In contrast, complex **7**, which was the most effective catalyst of the Ru–Cp family, achieved only 6% conversion in 6 h in 1:1 H_2_O/acetonitrile (Table S4). This result highlights the higher performance of the Ru–C-scorpionate complex **3** for catalyzing cycloadditions involving symmetrical internal alkynes and azides. This is a noteworthy result considering the typically reduced reactivity and increased steric hindrance associated with internal alkynes in AAC.

**Scheme 4:**
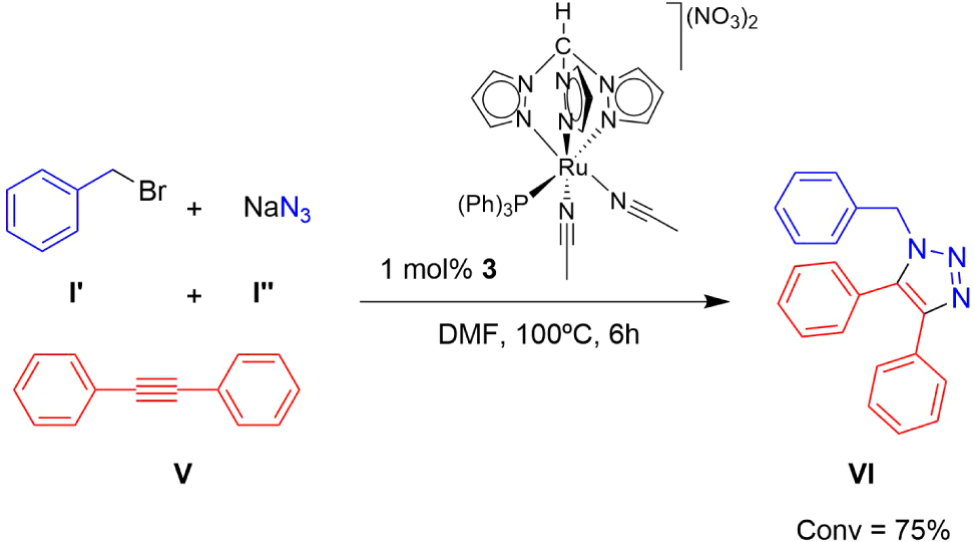
Catalytic Cycloaddition of Benzyl Bromide (**I’**), NaN_3_ (**I’’**), and Diphenylacetylene (**V**) into 1-Benzyl-4,5-diphenyl-1*H*-1,2,3-triazole (**VI**) Mediated by Complex **3** [Fn sch4-fn1]

i Conditions: 1 mol % **3**, DMF, **I** was formed *in situ* by the reaction of **I’** and **II’’** in a ratio of 1:1, **I/V** = 1:1.25, **I** = 0.55 M, and **V** = 0.70 M, without stirring. Conversion (%) was obtained by NMR, as an average of three experiments

The best results presented in this paper, which were obtained by using complex **3** and complex **7**, are summarized in Table 4, as well as compared with previously reported results obtained with catalysts containing the Cp, Cp*, Tp ligands, and other ruthenium complexes such as [RuH_2_(CO)­(PPh_3_)_3_]. Some conclusions can be drawn. First, the Ru­(II)–Tpm complex **3** exhibited higher regioselectivity in organic solvents compared to Ru­(II)–Cp complexes such as complex **7** and the previously reported [RuCpCl­(PPh_3_)_2_]. Also, complex **3** was shown to provide faster and higher conversions than related complexes containing Tp ligands. However, the catalytic activity of **3** was lower compared to complexes with Cp* such as [RuClCp*­(PPh_3_)_2_] and [RuClCp*]_4_. Additionally, complex **3** achieves a similar conversion for the synthesis of the trisubstituted triazole **VI** as related complexes, such as [RuTp­(Ph_3_)_2_N_3_], but does not achieve the conversions accomplished by [RuClCp*­(PPh_3_)_2_] for this reaction. Although efficient conversions were obtained with **7**, similar to other complexes containing Tp ligands, the selectivity was lower, with significant amounts of the 1,4-isomer being observed. On the other hand, the Ru­(II)–Cp complex **7** improved both the conversion rates from benzyl bromide (**I’**), NaN_3_ (**I’’**), and **II** and the selectivity of **III** in both aqueous and organic media compared to the previously published complex [RuCpCl­(PPh_3_)_2_]. Despite the clear good results obtained with the studied complexes, it was not possible to match the high regioselectivity reported in the literature for Cp*-containing complexes. Finally, complexes **3** and **7** also exhibited catalytic activity in methanol for the synthesis of the 1,4-isomer (**IV**), improving conversions and selectivity compared to complex [RuCl_2_(PPh_3_)_3_], but not comparable with that of [RuH_2_(CO)­(PPh_3_)_3_].

**Table 4:**
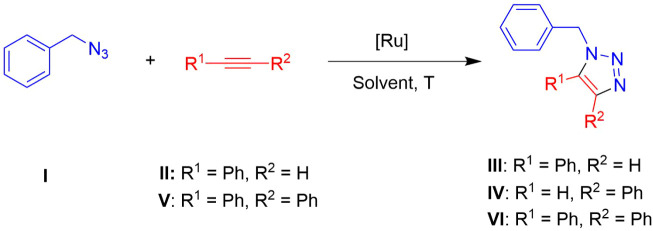
Preparation of 1,4,5-Trisubstituted-1,2,3-triazoles Mediated by Complexes Ru­(II)–C-Scorpionate (**3**) and Ru­(II) Cyclopentadienyl (**7**): Comparison with the Bibliographic Results^a^

						Conversion (%) /TOF (h^–1^)				
Catalyst	Substrate	[Ru] mol %	*t* (h)	*T* (°C)	Solvent	III	IV	VI	Conv. (%)	*ref*
[Ru(κ^3^-*N*-Tpm)(PPh_3_)(NCMe)_2_](NO_3_)_2_ (**3**)	**II**	1	6	100	DMF	76/13^c^	17/3 ^c^	–	>99 ^c^	this work^a^
	**II**	1	6	85	H_2_O/AcN	51/9^b^	49/8 ^b^	–	>99^b^	
	**II**	1	6	65	MeOH	48/8^b^	52/9 ^b^	–	>99^b^	
	**V**	1	6	100	DMF	–	–	75/1^c^	75^c^	
[RuClCp(PTA)_2_] (**7**)	**II**	1	6	65	MeOH	47/8^b^	53/9^b^	–	>99^b^	this work^a^
	**II**	1	6	100	H_2_O	52/9^b^	46/8^b^	–	98^b^	
	**II**	1	6	85	H_2_O/AcN	56/9^b^	41/7^b^	–	97^b^	
	**II**	1	1	85	H_2_O/AcN	37/37^b^	27/27^b^	–	64^b^	
	**II**	1	12	85	H_2_O/AcN	50^d^/8^e^	38^d^ /6^e^	–	8a8 ^d^	
	**II**	1	6	100	DMF	51/9^b^	41/7^b^	–	82	
	**V**	1	6	100	H_2_O/AcN	–	–	6/1^b^	6^d^	
[RuClCp(PPh_3_)_2_]	**II**	2	4	75	THF	13/7	1/0.1	–	14	^32^
[RuClCp(PPh_3_)_2_]	**II**	2	4	80.1	Benzene	43/5	71	–	50	^32^
[RuClCp*(PPh_3_)_2_]	**II**	2	0.5	65	THF	93/93	–	–	93	^22^
[RuClCp*(PPh_3_)_2_]	**V**	1	2	80.1	Benzene	–	–	99/45	>99	^22^
[RuCl(COD)Cp*]	**II**	2	4	75	THF	99/12	–	–	>99	^32^
[RuClCp*]_4_	**II**	2	0.3	RT	DMF	90/180	–	–	90	^31^
[RuCl_2_(PPh_3_)_3_]	**II**	2	4	75	THF	–	5/1	–	5	^32^
[RuH_2_(CO)(PPh_3_)_3_]^f^	**II**	2	2	80	H_2_O	–	84/21	–	84	^59^
[RuH_2_(CO)(PPh_3_)_3_]^f^	**II**	0.1	2	80	H_2_O	–	22/110	–	22	^59^
[RuTp(Ph_3_)_2_N_3_]	**II**	2	24	80	toluene	74/2	–	–	74	^39^
[RuTp(Ph_3_)_2_N_3_]	**II**	2	24	80	H_2_O	73/2	–	–	73	^39^
[RuTp(Ph_3_)_2_N_3_]	**V**	2	24	80	toluene	–	–	80/2	80	^39^
[RuTp(Ph_3_)(NH_2_Et)N_3_]	**II**	2	24	80	toluene	80/2	–	–	80	^38^
[RuTp(Ph_3_)(NH_2_Et)N_3_]	**II**	2	24	80	H_2_O	80/2	–	–	80	^38^
[RuTp(Ph_3_)(NCPh)N_3_]	**II**	2	24	80	toluene	50/1	–	–	74	^38^
[RuTp(Ph_3_)(NC(Ph)_2_)N_3_]	**II**	2	24	80	toluene	62/1	–	–	62	^38^

a TOF = turnover frequency, AcN = acetonitrile; ^a^ *this work*: conversion (%) obtained by ^1^H NMR, benzyl azide (**I**) was formed *in situ* by the reaction of phenyl bromide (**I’**) and NaN_3_ (**I’’**) in a ratio 1:1, **I**/**II** = 1:1.25, [**I**] = ^b^ 0.11 M and ^c^ 0.55 M, [**II**] = ^b^ 0.14 M and ^c^ 0.70 M. ^d^ Average total conversions, ^e^ TON and TOF after 2 cycles under N_2_ (H_2_O/AcN), 1 mol % [Ru], 6 h/run. *Comparison with bibliographic results:* [Ru] mol %, conversion (%), and TOF (h^–1^) calculated from references 52,53,29,36 , and^35^. ^f^ [RuH_2_(CO)­(PPh_3_)_3_] was used with an additive.

## Mechanism Insights

### DFT Studies and NMR Studies

Several computational works in the literature provide a theoretical mechanistic investigation of the RuAAC systems. A similar mechanism is proposed for [RuClCp*],^32,68^ [RuClCp],^34^ and [RuTp] complexes.^38^ In all cases, the formation of a Ru–azide–alkyne complex is suggested as the first step, leading to the formation of a 6-membered ruthenacycle intermediate, which subsequently undergoes reductive elimination and the formation of the triazole product. DFT studies highlight the importance of electronic and steric hindrance conferred by the Cp* ligand, compared to the Cp ligand, in positively influencing the selective formation of 1,5-disubstituted triazole.^34^ While the mechanisms for {RuClCp} and {RuClCp*} complex species are relatively well-established, those for {RuTp} remain less elucidated, and no works have been published for {RuTpm} complex species.

First, it has to be noted that the Cp ligand has a larger donor ability than Tpm. This can be confirmed and quantified by Energy Decomposition Analysis (EDA),^69^ coupled with the Natural Orbitals for Chemical Valence (NOCV) theory.^70^ Indeed, comparing (Cp)^−^···[RuCl­(NCCMe)­(PH_3_)]^+^ and (κ^3^-Tpm)···[RuCl­(NCCMe)­(PH_3_)]^+^ bonds, the former shows a σ-donation component of −155 kcal/mol and a π back-donation of −31 kcal/mol, while, for the latter, the σ-donation component amounts to −112 kcal/mol and π back-donation to −28 kcal/mol (see Supporting Information for details).

To shed more light on the reaction, different mechanism paths were modeled and compared. First, the speciation in solution of complex [Ru­(κ^3^-*N*-Tpm)­(PH_3_)­(NCMe)_2_]^2+^ (**3′**), obtained by simplifying complex **3** to optimize computational resources, was investigated.^71^ Considering the presence in solution of the N_3_^–^ anion, which has a remarkable coordinating ability, the substitution of one or more ligands with azide was evaluated (Table 5). All DFT-optimized geometries for the speciation of **3′** are in Figure S15.

**Table 5:** Free Gibbs Energies (Δ*G*, in Kcal/Mol) for [Ru­(κ^*n*^-*N*-Tpm)­(L1)­(L2)­(L3)­(L4)]^*m*+^ ^a^

Label	L1	L2	L3	L4	*n*	*m*	Δ*G*
**3′**	PH_3_	NCMe	NCMe	-	3	2	0.0
**3′a**	PH_3_	N_3_	N_3_	-	3	0	–12.5
**3′b**	N_3_	NCMe	NCMe	-	3	1	0.5
**3′c**	N_3_	N_3_	NCMe	-	3	0	2.0
**3′d**	PH_3_	N_3_	NCMe	-	3	1	–7.2
**3′e**	PH_3_	N_3_	NCMe	NCMe	2	1	6.4
**3′f**	PH_3_	N_3_	N_3_	NCMe	2	0	0.9
**3′g**	PH_3_	BnN_3_	PhCCH	-	3	2	12.2
**3′h**	N_3_	BnN_3_	PhCCH	-	3	1	12.0
**3′i**	PH_3_	N_3_	BnN_3_	PhCCH	2	1	20.4

a The energy of **3′** was taken as reference.

The coordination of an azide is beneficial for the stability of the complex, with **3′a** ([Ru­(κ^3^-*N*-Tpm)­(PH_3_)­(N_3_)_2_] being the most stable one, bearing the phosphine and two azides, giving a neutral complex. Anyway, two of these ligands must be displaced to coordinate the reaction substrates, and three different intermediates can be compared. One bears the phosphine, [Ru­(κ^3^-*N*-Tpm)­(PH_3_)­(BnN_3_)­(PhCCH)]^2+^ (**3′g**), another bears the azide, [Ru­(κ^3^-*N*-Tpm)­(N_3_)­(BnN_3_)­(PhCCH)]^+^ (**3′h**) and, considering that the Tpm ligand can also assume a κ^2^ coordination mode, the last one bears both the phosphine and the azide, [Ru­(κ^2^-*N*-Tpm)­(N_3_)­(PH_3_)­(BnN_3_)­(PhCCH)]^+^ (**3′i**). Evidently, the coordination of the alkyne and the benzyl azide produces a less stable intermediate species. This is particularly true for **3′i**, for which the entropic gain in bearing an additional ligand is not counterbalanced by the entropic loss. The reaction between benzyl bromide and the azide to give benzyl azide possesses a low activation barrier (Δ*G* ^⧧^ = 4.1 kcal mol, see Supporting Information) and a negative Δ*G*_rxn_ (−16.5 kcal/mol).

Starting from **3′g–i**, two reaction paths were computed by DFT for each intermediate: one for the attack of the benzyl azide on the terminal alkyne carbon (path “a”) and one for the attack on the internal alkyne carbon (path “b”), which eventually lead to products **III** and **IV**, respectively (Figure S16, S22, and 3). The solvent effect was simulated through the continuum-like polarizable continuum model (C-PCM, DMF). In all cases, the reaction proceeds with a two-step mechanism: after the attack of the azide on the alkyne (TS1), an intermediate is formed (INT1), which evolves with ring-closure (TS2) and leads to the coordinated product (INT2). Energies for all the species involved in the mechanism are listed in Table 6, and their structures are shown in Figure S17–S20 and Figure S23–S24.

**Table 6:** Free Gibbs energies^a^ (Δ*G*, in kcal/mol) and, in Parentheses, Enthalpies (Δ*H*, in kcal/mol) for the Species Involved in the Mechanism^b^

Starting intermediate	TS1	INT1	TS2	INT2
**3′g** (path a)	44.1 (37.8)	33.3 (26.7)	39.7 (31.8)	–4.3 (−11.3)
**3′g** (path b)	50.8 (44.5)	44.8 (38.6)	47.8 (40.0)	–17.7 (−27.4)
**3′h** (path a)	40.3 (34.3)	25.9 (20.2)	32.9 (25.1)	–9.8 (−15.9)
**3′h** (path b)	44.6 (39.1)	24.4 (19.0)	31.5 (24.1)	–20.3 (−28.8)
**3′i** (path a)	54.4 (37.3)	31.7 (15.2)	65.7 (47.4)	–27.0 (−44.5)
**3′i** (path b)	59.2 (42.5)	38.8 (21.2)	35.7 (17.3)	–36.7 (−53.9)

a All the energy values are referred to **3′**.

b The energy of **3′** was taken as reference.

**Figure 3:**
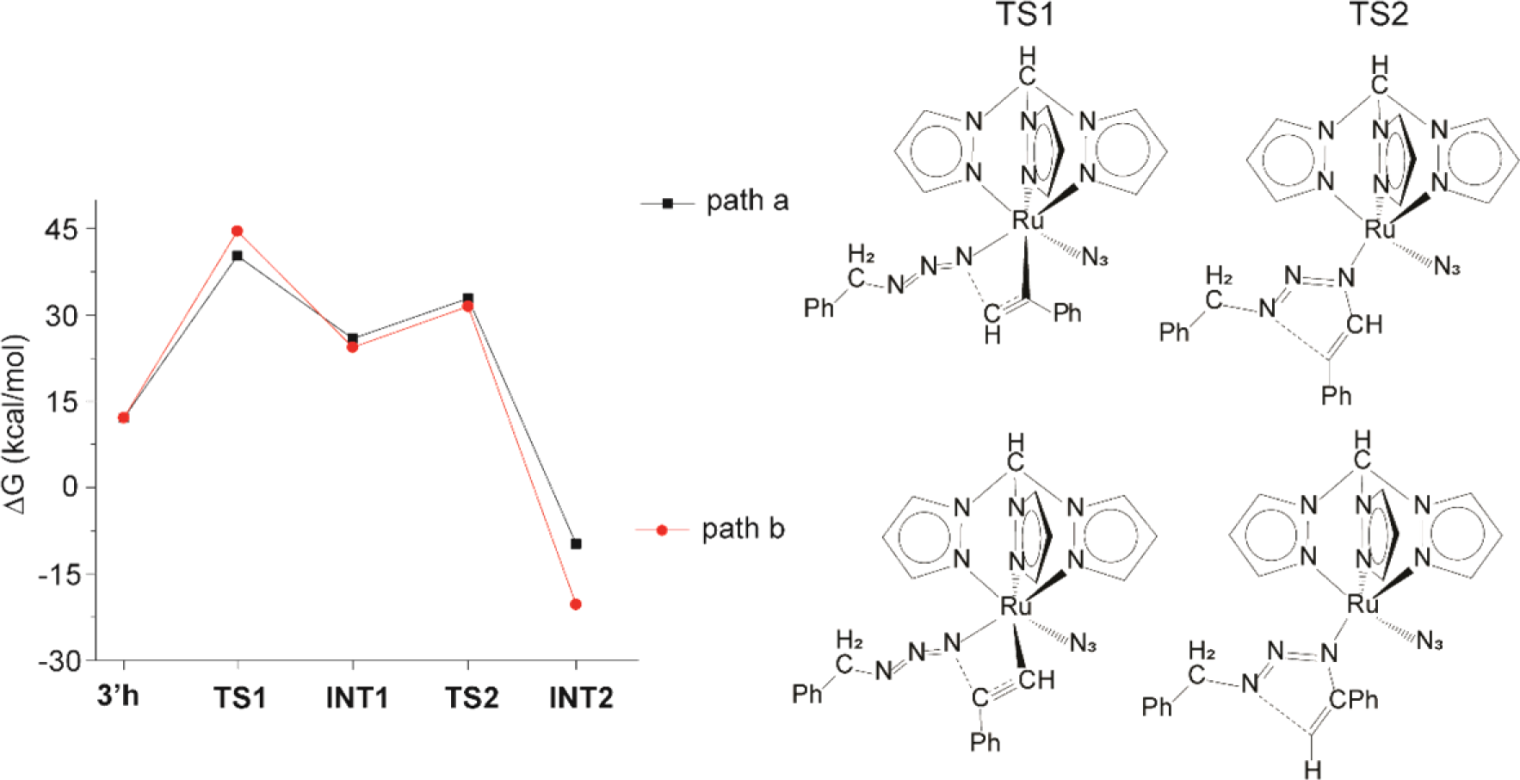
Energy paths and transition state structures for the reaction between **3′h**, PhCCH, and BnN_3_. The energy of **3′** was taken as reference.

DFT results clearly indicate that the shift of the coordination mode of the Tpm ligand from κ^3^ to κ^2^ does not lead to a lower activation barrier. The main obstacle is the entropic loss associated with coordinating an additional ligand, and this is evident from the difference between Δ*G* and Δ*H* values. On the other hand, the substitution of the phosphine with the azide makes the reaction slightly more favorable. About the regioselectivity of the reaction, path “a” is always kinetically favored but thermodynamically unfavored compared to path “b”. The observed regioselectivity can be explained by the fact that path “a” is kinetically preferred, although thermodynamically less favorable than path “b”. This can be attributed to subtle geometrical differences in the corresponding TS1 structures (Figure S21). In both transition states, the phenyl group of BnN_3_ engages in a π–π interaction with a pyrazolyl ring of Tpm, but in **3′h_TS1_a,** the interaromatic distance is slightly shorter (3.516 Å) than in **3′h_TS1_b** (3.566 Å). Moreover, only in **3′h_TS1_a,** does the phenyl group of the alkyne orient one of its C–H bonds toward the center of a pyrazolyl ring, suggesting a weak CH/π stabilizing interaction. Such an interaction is absent in **3′h_TS1_b** and could be favored in aprotic solvents such as DMF. These combined effects result in a slightly more stabilized TS1 for path “a” relative to path “b”.

When the products detach from the metal center, **IV** is more stable than **III** by about 1.6 kcal/mol. This result is consistent with the experimental observation, where compound **III** is the major product in DMF. As previously discussed, the formation of transition states **3′h_TS1_a** or **3′h_TS1_b** could depend on the solvent in which the reaction was carried out. Thus, in aprotic solvents, the preferred route should involve the formation of **3′h_TS1_a**. It is important to report that varying the dielectric of the solvent (treated as a continuum) has a negligible effect on the Δ*G* between the rate-determining steps of path “a” and “b”.

The most stable species found by DFT is the intermediate **3′a**, in which, after the prior release of one PH_3_ and N_3_^–^, compounds **I** and **II** coordinate to the metal, affording complex **3′h**. This species is the most stable, as it requires the lowest Gibbs energy (12.0 kcal/mol) compared to other possible intermediates **3′g–i**. From **3′h**, the reaction can proceed by two different pathways (Scheme 5). The most energetically favored pathway, path “a” (Δ*G* = 40.3 kcal/mol, Figure 3), leads to the formation of the intermediate TS1 by reaction of the coordinated azide–N atom with the alkyne of molecule **II**, giving rise to the intermediate INT1. This species suffers the attack of the nitrogen atom Bn-N on the remaining double C=C bond, which evolves toward ring-closure, providing intermediate INT2 via TS2. Finally, compound **III** is released, regenerating the specie **3′a**. When the reaction progresses by path “b,” the compound obtained is **IV**.

**Scheme 5:**
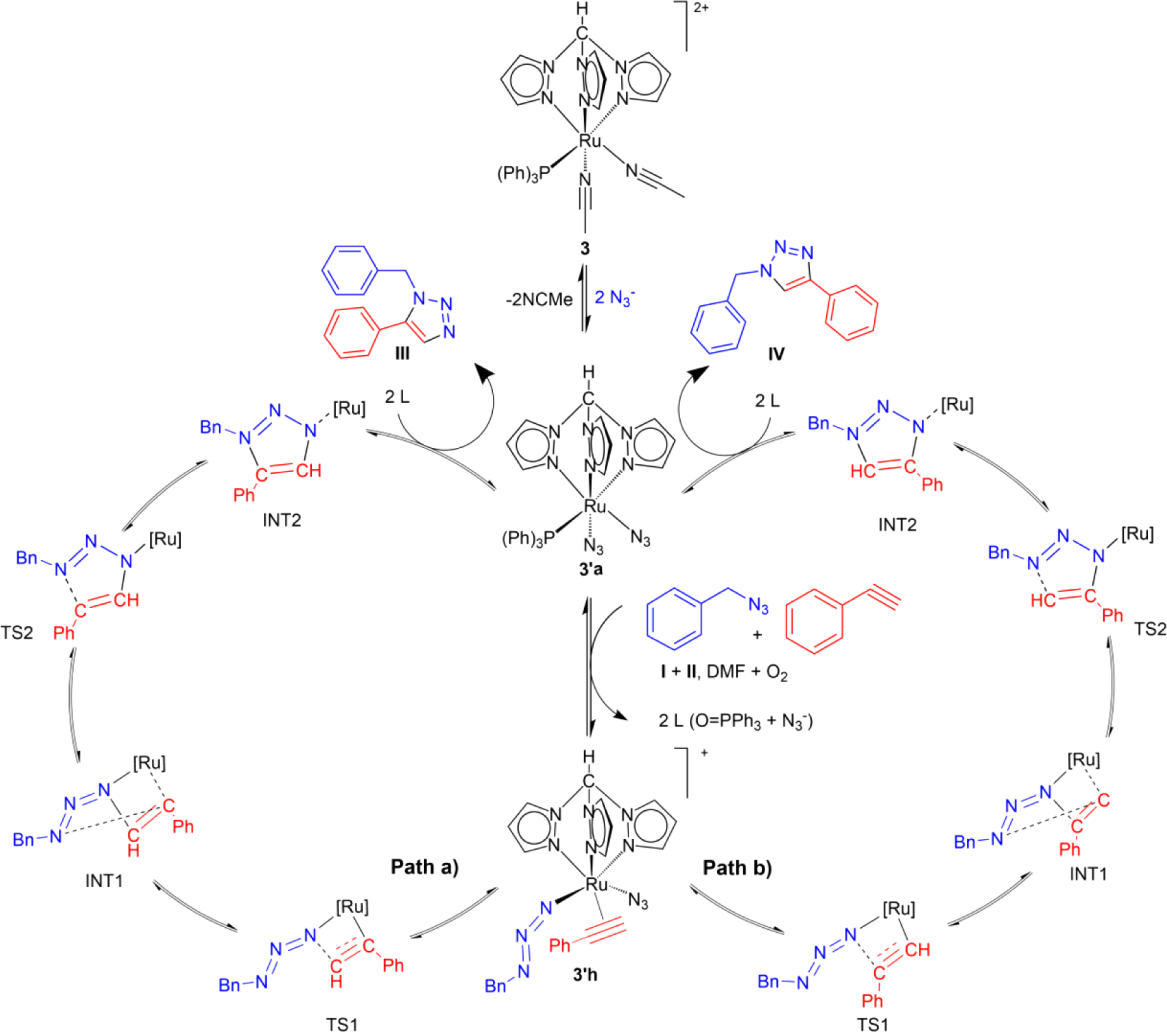
Proposed Mechanism (DFT Calculation) for the Cycloaddition of Benzyl Azide (**I**) and Phenylacetylene (**II**) into 1-Benzyl-5-phenyl-1H-1,2,3-triazole (**III**) (Path “a”) and 1-Benzyl-4-phenyl-1H-1,2,3-triazole (**IV**) (Path “b”) Catalyzed by Complex **3** in DMF

To experimentally locate the proposed intermediates of the reaction, a study using ^31^P­{^1^H} NMR spectroscopy was carried out in DMF (D_2_O capillary). The compound **I** (0.55 M) with **II** (0.70 M) were reacted in the presence of complex **3** (1 mol %), and after 1 h, only a signal at 47.9 ppm was observed, which can be ascribed to the starting complex, but after 5 h (Figure S25), four singlets arose at 23.7 ppm (8%), 26.4 ppm (9%), 41.5 ppm (21%), and 51.3 ppm (62%). The signal at 26.4 ppm was assigned to O=PPh_3_, indicating that PPh_3_ was released during the reaction. The species **3′h** has not constituted with phosphine and therefore does not display any signal in the ^31^P­{^1^H} NMR. Nevertheless, the other possible species that could be afforded in the reaction, **3′g** and **3′i**, as suggested by the DFT calculation, should certainly display signals around the starting complex. As the calculation indicates that these intermediates are less stable than **3′h**, with **3′i** being the most stable, it is reasonable to assign the signal at 23.7 ppm to this intermediate and the signal at 41.5 ppm to **3′g**. Finally, the most intense peak at 51.3 ppm was unambiguously assigned to species **3′a**; additional experimental evidence was obtained to support this assignment. The reaction between complex **3** and an excess of NaN_3_ (100 equiv) was monitored by ^31^P­{^1^H} NMR in DMF-d7. At the initial time, it displayed only two signals: one at 51.3 ppm, corresponding to **3′a**, and another at 48.5 ppm, corresponding to complex **3** (Figure S26).

The resulting mixture was then heated at 100 °C for 1 h, subsequently evaporated to dryness, redissolved in MeOH, and analyzed by TOF-MS (Figure S27). Two major ions were observed at *m*/*z* 602.16 and 726.15. These ions are consistent with the adducts [Ru­(κ^3^-*N*-Tpm)­(PPh_3_)]­H·Na^+^ (Figure S28) and [Ru­(κ^3^-*N*-Tpm)­(PPh_3_)­(N_3_)_2_]·(CH_3_OH)_2_^+^ (Figure S29), respectively. The latter corresponds to the adduct of two methanol molecules with intermediate **3′a**, which further supports the proposed mechanism.

## Experimental Section

### Materials and Methods

All chemicals were of reagent grade and, unless otherwise stated, were used as received from commercial suppliers. Similarly, all reactions were carried out under a nitrogen (N_2_) atmosphere using standard Schlenk-tube techniques. Likewise solvents were dried and deoxygenated before use and through standard procedures. Hydrotris­(1-pyrazolyl)­methane (Tpm) was prepared according to a published procedure.^72^ The synthesis and characterization of [Ru­(κ^3^-*N*-Tpm)­(NCMe)_3_]­(NO_3_)_2_ (**1**) are described in Supporting Information (Section 2, Synthesis and Characterization of Ru–C-Scorpionate Complexes). The ruthenium­(II)–C-scorpionate complexes [RuCl­(κ^3^‑*N*-Tpm)­(PPh_3_)­(NCMe)]­Cl (**2**),^44^ [Ru­(κ^3^ *-N*-Tpm)­(κ^2^ *-N*-C_5_H_4_NCH_2_NH)­(PPh_3_)]­(NO_3_)_2_, (**4**) and [Ru­(κ^3^ *-N*-Tpm)­(κ^2^ *N*-H_2_NCH_2_CH_2_NH_2_)­(PPh_3_)]­(NO_3_)_2_ (**5**)^45^ were prepared according to the literature. Procedures for complexes [Ru­(κ^3^-*N*-Tpm)­(PPh_3_)­(NCMe)_2_]­(NO_3_)_2_ (**3**) and [RuCl­(κ^3^-*N*-Tpm)­(PPh_3_)­(κ*N*-NH_2_CH_2_CH_2_OH)]Cl (**6**),^46^ and full details of their synthesis and characterization are also provided in Section 2 of Supporting Information. Ruthenium–cyclopentadienyl complexes containing adamantane-like ligands, [RuClCp­(PTA)_2_] (**7**), [RuCp­(OH_2_)­(PTA)_2_]­CF_3_SO_3_ (**8**),^50,51^ [RuClCp­(mPTA)_2_]­(CF_3_SO_3_)_2_ (**9**),^52^ [RuCp­(OH_2_)­(mPTA)_2_]­(CF_3_SO_3_)_3_ (**10**)^53,54^ (PTA = 1,3,5-triaza-7-phosphaadamantane, mPTA = *N*-methyl-1,3,5-triaza-7-phosphaadamantane), were prepared according to the literature. ^1^H, ^13^C­{^1^H}, and ^31^P­{^1^H} spectra were recorded on a Bruker Avance III HD 300 spectrometer operating at 300.13 MHz (^1^H), 75.47 MHz (^13^C), and 121.49 MHz (^31^P), and on a Bruker Nanobay Avance III HD 400 MHz spectrometer operating at 400.13 MHz (^1^H), 100.61 MHz (^13^C), and 161.975 MHz (^31^P). Peak positions are relative to tetramethylsilane and were calibrated against the residual solvent resonance (^1^H), relative to the deuterated solvent multiplet (^13^C­{^1^H}) and to external 85% H_3_PO_4_ (^31^P­{^1^H}). All NMR spectra were recorded at 25 °C. IR spectra of solid samples were recorded on an Agilent Cary630 FTIR spectrometer. Elemental analyses were performed on a Vario MICRO cube instrument (Elementar). The mass spectrum was recorded on a dual-TIMS analyzer timsTOF Pro 2, Bruker (INBIO, University of Cadiz).

### Synthesis and Characterization of Ruthenium C-Complexes: [Ru­(κ^3^-*N*-Tpm)­(NCMe)_3_]­(NO_3_)_2_ (1)

A mixture of RuCl_3_(κ^3^-*N*-Tpm)·1.5H_2_O (200 mg, 0.464 mmol) and AgNO_3_ (238 mg, 1.40 mmol) in 40 mL of an acetonitrile/water mixture (v/v 3:1) was heated at reflux for 8 h under light protection. After cooling to room temperature, the mixture was filtered through Celite, and the solvent was evaporated under reduced pressure. The crude product was redissolved in the minimum volume of water and filtered through Celite. The volatiles were evaporated under reduced pressure. The obtained powder was dissolved in the minimum volume of acetone/MeOH and precipitated with diethyl ether. The resulting mixture was filtered, and the isolated solid was dried under vacuum. Purple solid, yield: 188 mg (72%). Anal. Calcd for C_16_H_19_N_11_O_6_Ru: C, 34.17; H, 3.40; N, 27.39. Found: C, 34.15; H, 3.32; N, 27.42. IR (solid state): υ̃/cm^—1^ = 3131w, 3109w, 2990w, 2933w, 1909m, 1661m, 1514m, 1406–1250s (υ̃_NO3_), 1091m, 1057m, 1036m, 993m, 859m, 822w, 779s, 757s. ^1^H NMR (CD_3_OD): δ/ppm = 9.66 (s, 1H, C^δ^H); 8.46 (d, 3H, ^3^ *J*_HH_ = 2.8 Hz, C^γ^H); 8.24 (d-br, 3H, C^a^H); 6.68 (t-br, 3H, ^3^ *J*_HH_ = 2.6 Hz, C^β^H); 2.50 (s, 9H, C^2^H). ^13^C NMR (CD_3_OD): 148.6 (C^a^); 136.2 (C^γ^); 127.7 (C^1^); 110.3 (C^β^); 3.3 (C^2^). ^1^H NMR (D_2_O): δ/ppm = 9.44 (s, 1H, C^δ^H); 8.32 (d, 3H, ^3^ *J*_HH_ = 3.0 Hz, C^γ^H); 8.11 (d, 3H, ^3^ *J*_HH_ = 2.3 Hz, C^a^H); 6.59 (t, 3H, ^3^ *J*_HH_ = 2.3 Hz, C^β^H); 2.50 (s, 9H, C^2^H).

### [Ru­(κ^3^-*N*-Tpm)­(PPh_3_)­(NCMe)_2_]­(NO_3_)_2_ (3)^46^

A mixture of [RuCl­(κ-^3^ *N*-Tpm)­(PPh_3_)_2_] (100 mg, 0.110 mmol) and AgNO_3_ (37 mg, 0.22 mmol) in 10 mL of acetonitrile (MeCN) was heated at reflux for 16 h. After cooling to room temperature, the mixture was filtered through Celite, and the solvent was evaporated under reduced pressure. The crude product was redissolved in the minimum volume of dichloromethane, filtered on Celite, and the volatiles were evaporated under reduced pressure. The obtained powder was dispersed in THF and left stirring for 1 h. The solid was eventually filtered, washed with diethyl ether, and dried under vacuum. White solid, 85 mg (98%). Anal. Calcd for C_32_H_31_N_10_O_6_PRu: C, 49.04; H, 3.99; N, 17.87. Found: C, 49.11; H, 3.95; N, 17.93. IR (solid state): υ̃/cm^–1^ = 3153w, 3128w, 3108w, 2980w, 2955w, 2924w, 1740w, 1513w, 1479w, 1457w, 1431ww, 1400m, 1374m (NO_3_), 1315s, 1250m, 1227m, 1182w, 1088m, 1051m, 994w, 861m, 827m, 779s, 747s, 696s. ^1^H NMR (CD_3_OD): δ/ppm = 9.88 (s, 1H, C^δ^H); 8.55 (d, 2H, ^3^ *J*_HH_ = 2.9 Hz, C^γ^H); 8.50 (d, 1H, ^3^ *J*_HH_ = 2.8 Hz, C^γ^H); 8.46 (d, 1H, ^3^ *J*_HH_ = 2.2 Hz, C^α^H); 7.04 (d, 2H, ^3^ *J*_HH_ = 2.3 Hz, C^α^H); 7.57 (m, 3H, C^6^H); 7.47 (m, 6H, C^4^H or C^5^H); 7.17 (m, 6H, C^4^H or C^5^H); 6.72 (t-br, 1H, C^β^H); 6.39 (t, 2H, ^3^ *J*_HH_ = 2.6 Hz, C^β^H); 2.51 (s, 6H, C^2^H). ^13^C NMR (CD_3_OD): δ/ppm = 149.7 (2C^α^); 146.8 (C^α^H); 137.3 (2C^γ^); 135.4 (C^γ^); 134.9 (d, ^2^ *J*_CP_ = 9.8 Hz, C^4^); 132.3 (d, ^4^ *J*_CP_ = 2.4 Hz, C^6^); 131.1 (d, ^1^ *J*_CP_ = 44.6 Hz, C^3^); 130.1 (d, ^3^ *J*_CP_ = 9.7 Hz, C^5^); 129.3 (C^1^); 110.35 (3C^β^); 3.76 (C^2^). ^31^P­{^1^H} NMR (CD_3_OD): δ/ppm = 46.9. ^1^H NMR (CDCl_3_): δ/ppm = 11.31 (s, 1H, C^δ^H); 8.83 (d-br, 1H, C^α^H); 6.75 (d, 2H, ^3^ *J*_HH_ = 2.3 Hz, C^α^H); 8.50 (d, 2H, ^3^ *J*_HH_ = 2.3 Hz, C^γ^H); 8.47 (d, 1H, ^3^ *J*_HH_ = 2.8 Hz, C^γ^H); 7.51 (t-br, 3H, C^6^H); 7.40 (t, 6H, ^3^ *J*_HH_ = 7.9 Hz, C^4^H or C^5^H); 7.10 (t, 6H, ^3^ *J*_HH_ = 7.8 Hz, C^4^H or C^5^H); 6.59 (t, 1H, ^3^ *J*_HH_ = 2.6 Hz, C^β^H); 6.18 (t, 2H, ^3^ *J*_HH_ = 2.6 Hz, C^β^H); 2.56 (s, 6H, C^2^H). ^31^P­{^1^H} NMR (CDCl_3_): δ/ppm = 47.9.

### [RuCl­(κ^3^-*N*-Tpm)­(PPh_3_)­(k*N*-NH_2_CH_2_CH_2_OH)]­Cl (6)^46^

A solution of [RuCl­(κ^3^-*N*-Tpm)­(PPh_3_)_2_] (120 mg, 0,132 mmol) and ethanolamine (17 μL, 0.27 mmol) in THF (8 mL) was heated at reflux for 16 h. The product precipitated from the reaction medium as a fine yellow powder. After cooling to room temperature, the solid was filtered, washed with THF and diethyl ether, and dried under vacuum. Yellow solid, yield: 92 mg (98%). Anal. Calcd for C_30_H_32_Cl_2_N_7_OPRu: C, 50.78; H, 4.55; Cl, 9.99; N, 13.82. Found: C, 50.65; H, 4.44; Cl, 9.94; N, 13.85. IR (solid state): υ̃/cm^–1^ = 3405w (OH), 3295w (NH), 3106w, 3070w, 2984w, 2969w, 2899w, 2872w, 1508w, 1481w, 1453w, 1434m, 1405w, 1289m, 1273w, 1254m, 1227w, 1187w, 1088s, 1068m, 1050m, 854m, 794m, 768s, 761s, 743s, 694s, 683m, 613w, 607w, 530s, 511s, 501s, 452m, 421w. ^1^H NMR (CD_3_OD): δ/ppm = 9.63 (s, 1H, C^δ^H); 8.46, 8.44, 8.42 (d, 3H, ^3^ *J*_HH_ = 2.9 Hz, C^γ^H); 8.34, 7.28, 7.05 (d, 3H, ^3^ *J*_HH_ = 2.2 Hz, C^α^H); 7.48 (m, 3H, C^6^H); 7.40–7.32 (m, 12H, C^4^H + C^5^H); 6.68, 6.31, 6.21 (t, 1H, ^3^ *J*_HH_ = 2.6 Hz, C^β^H); 3.37 (t-br, 1H, NH_2_); 3.28 (m, 2H, C^2^H); 2.70 (t-br, 1H, NH_2_); 2.39 (m, 1H, C^1^H); 1.99 (m, 1H, C^1^H). *OH not observed.* ^31^P NMR (CD_3_OD): δ/ppm = 53.0. ^13^C­{^1^H} NMR (CD_3_OD): δ/ppm = 151.5, 150.1, 146.4 (C^α^); 136.1, 135.9, 134.7 (C^δ^); 134.9 (d, ^2^ *J*_CP_ = 9.4 Hz, C^4^); 133.5 (d, ^1^ *J*_CP_ = 40.1 Hz, C^3^); 131.4 (d, ^4^ *J*_CP_ = 1.8 Hz, C^6^); 129.8 (d, ^3^ *J*_CP_ = 9.2 Hz, C^5^); 109.8, 109.7, 109.6 (C^β^H); 62.43 (C^2^H); 48.3 (C^1^H).

### General Procedure for the Catalytic Cycloaddition of Benzyl Azide and Alkynes (Phenylacetylene and Diphenylacetylene) Catalyzed by the Half-Sandwich Ruthenium­(II) Complexes Containing Hydrotris­(pyrazolyl)­methane and Cyclopentadienyl Ligands under Several Conditions

The catalytic activity of ruthenium complexes was evaluated for the catalytic cycloaddition of benzyl azide (**I**) and phenylacetylene (**II**) into 1-benzyl-5-phenyl-1H-1,2,3-triazole (**III**) and 1-benzyl-4-phenyl-1H-1,2,3-triazole (**IV**) in H_2_O, H_2_O/acetonitrile (1:1), MeOH, dichloromethane (DCM), and dimethylformamide (DMF), depending on the solubility of each complex, under N_2_. In several reactions, varying substrate concentrations in solvents were assessed. In a typical reaction, to a mixture of benzyl bromide (**I’**) (0.33 mmol), NaN_3_ (**I’’**) (0.33 mmol), the required amount of phenylacetylene (**II**) (0.4125 mmol), and 0.6 or 3 mL of solvent was added with 1 mol % of the required catalyst (0.0033 mmol). The solution was stirred regularly with a magnetic bar at the required temperature. Experiments were conducted at the reflux temperatures of CH_2_Cl_2_ (40 °C), MeOH (65 °C), H_2_O (100 °C), H_2_O/acetonitrile 1:1 (approximately 85 °C), and at 100 °C when DMF was used. The catalytic activity of [Ru­(κ^3^-*N*-Tpm)­(PPh_3_)­(NCMe)_2_]­(NO_3_)_2_ (**3**) and [RuClCp­(PTA)_2_] (**7**) was also evaluated for the catalytic cycloaddition of benzyl azide (**I**) and diphenylacetylene (**V**) into 1-benzyl-4,5-diphenyl-1*H*-1,2,3-triazole (**VI**) in DMF. At the chosen time (1, 3, 5, or 6 h), each reaction evaluated was cooled to room temperature. After, 3 mL of H_2_O was added to the solution, extracted with ethyl acetate (3 × 3 mL), and the collected organic layers were dried over MgSO_4_, and vacuum-dried. A single series of reactions was performed in each of the solvents, using each complex (**1**–**10**) as a catalyst, to identify the complexes with the best catalytic activity under optimal conditions. Once the best results were selected, three replicates of each reaction were performed. The standard deviations of these reactions are included in Table S3 and S4. The product conversions were obtained by ^1^H NMR. Conversions (%) presented are the average of three experiments. The identity of the resulting triazole compounds was assessed by comparison with commercially available pure samples. In all reactions, product conversions were calculated relative to benzyl azide (**I**) and the triazole products, **III** and **IV**.

### Computational Details

All geometries were optimized with ORCA 6.0.0 (cit. 10.1002/wcms.1606) using the B97–3c functional with the zero-order regular approximation (ZORA) (cit. 10.1063/1.476576) to take relativistic effects into account, and in conjunction with a triple-ζ quality basis set (ZORA-TZVP). For ruthenium, the basis set “old-ZORA-TZVP” was used. The dispersion corrections were introduced using the Grimme D3-parametrized correction and the Becke–Johnson damping to the DFT energy (cit. 10.1063/1.3382344), All the structures were confirmed to be local energy minima (no imaginary frequencies) or saddle points (one imaginary frequency corresponding to the reaction coordinate). The solvent was considered through the continuum-like polarizable continuum model (C-PCM, DMF). EDA-NOCV analysis was performed using ORCA 6.1.0, as described in the literature.^73^ The solvent effect was explored by varying the solvent in the C-PCM model (water, methanol, dichloromethane) and performing a single-point calculation on the optimized geometries of **3′g**, **TS1,** and **Int1** (paths “a” and “b”).

## Conclusions

A series of Ru­(II) complexes containing hydrotris­(pyrazolyl)­methane ligands (Tpm), namely ([Ru­(κ^3^-*N*-Tpm)­(NCMe)_3_]­(NO_3_)_2_ (**1**), [RuCl­(κ^3^-*N*-Tpm)­(PPh_3_)­(NCMe)]Cl (**2**), [Ru­(κ^3^-*N*-Tpm)­(PPh_3_)­(NCMe)_2_]­(NO_3_)_2_ (**3**), [Ru­(κ^3^-*N*-Tpm)­(κ^2^-*N*-C_5_H_4_NCH_2_NH_2_)­(PPh_3_)]­(NO_3_)_2_ (**4**), [Ru­(κ^3^-*N*-Tpm)­(κ^2^-*N*-H_2_N CH_2_CH_2_NH_2_)­(PPh_3_)]­(NO_3_)_2_ (**5**) and [RuCl­(κ^3^-*N*-Tpm)­(PPh_3_)­(κ^1^ *-N*-NH_2_(CH_2_)_2_OH)]Cl (**6**)) and Cp ligand [RuClCp­(PTA)_2_] (**7**), [RuCp­(OH_2_)­(PTA)_2_]­CF_3_SO_3_ (**8**), ([RuClCp­(mPTA)_2_]­(CF_3_SO_3_)_2_ (**9**), and [RuCp­(OH_2_)­(mPTA)_2_]­(CF_3_SO_3_)_3_ (**10**) (PTA = 1,3,5-triaza-7-phosphaadamantane; mPTA = *N*-methyl-1,3,5-triaza-7-phosphaadamantane), were evaluated as catalysts for the azide/alkyne cycloaddition click in aqueous and organic media. The highest conversion rates and selectivity for the synthesis of 1,5-disubstituted triazoles were achieved in water, acetonitrile/water, and dimethylformamide, while 1,4-disubstituted products were slightly favored in methanol.

Among them, the Ru­(II)–C-scorpionate complexes **3** and **4** exhibited superior regioselectivity and conversion rates in DMF during the catalytic cycloaddition of benzyl bromide (**I’**), NaN_3_ (**I’’**), and phenylacetylene (**II**), leading to a larger formation of 1,5-disubstituted triazole (**3**: 93%, **III**/**IV** = 4.5:1 ratio, **4**: 92%, **III**/**IV** = 3.6:1 ratio). Notably, complex **3** was also active in the cycloaddition reaction between **I’** and **I’’** with an internal alkyne (diphenylacetylene), reaching a TON of 75 (TOF = 12.5 h^–1^). Computational and NMR spectroscopic studies provide insight into the reaction mechanism, indicating the formation of [Ru­(κ^3^-*N*-Tpm)­(PPh_3_)­(N_3_)_2_] (**3′a**), which acts as a precursor of the active intermediate necessary for the 1,5-triazole synthesis. The formation intermediate (**3′a**) was elucidated by both ^31^P­{^1^H} NMR and MS spectroscopy. Among the remaining potential intermediates, [Ru­(κ^3^-*N*-Tpm)­(PH_3_)­(BnN_3_)­(PhCCH)]^2+^ (**3′g**), [Ru­(κ^3^-*N*-Tpm)­(N_3_)­(BnN_3_)­(PhCCH)]^+^ (**3′h**), and [Ru­(κ^2^-*N*-Tpm)­(N_3_)­(PH_3_)­(BnN_3_)­(PhCCH)]^+^ (**3′i**), the most likely active species is **3′h**, based on ^31^P­{^1^H} NMR spectroscopy evidence. The hypothesis suggests that the reaction proceeds through a two-step process involving an azide attack on the coordinated terminal alkynyl fragment, followed by ring closure. The regioselectivity analysis indicates that the kinetic pathway favors the formation of the 1,5-isomer (**III**), in line with experimental findings. The proposed mechanism is consistent with those of other previously reported RuAAC systems.

Overall, this study highlights the potential of Ru­(II)–piano-stool and Ru­(II)–C-scorpionate complexes in AAC reactions, particularly in aqueous and biphasic systems, and provides valuable mechanistic insights into their catalytic behavior.

## Supplementary Material



## Notes

Material and methods; Synthesis and characterization of Ru–C-scorpionate complexes; General procedure for the catalytic cycloaddition of benzyl azide and the alkynes, phenylacetylene and diphenylacetylene catalyzed by the half-sandwich ruthenium­(II) complexes containing hydrotris­(pyrazolyl)­methane and cyclopentadienyl ligands in several conditions; Reactivity of complex **3** along the cycloaddition of phenyl azide and phenylacetylene in dimethylformamide, followed by ^31^P­{^1^H} NMR spectroscopy; Computational details (PDF)
