# Supplementary material for: Catalytic Activity of Ruthenium Complexes Containing Hydrotris(pyrazolyl)methane or Cyclopentadienyl Ligands in the Azide–Alkyne Click Cycloaddition Reaction
Source: ACS Omega. 2026 Mar 18;11(12):18825–39. doi: 10.1021/acsomega.5c10216 (PMC13044660; doi:10.1021/acsomega.5c10216)
Supplement: Supplementary file 1 [file ao5c10216_si_006.pdf]

Catalytic activity of ruthenium complexes containing hydrotris(pyrazolyl)methane or cyclopentadienyl ligands in the azide-alkyne click cycloaddition reaction

Supporting Information

Belén López-Sánchez[1], Alberto Gobbo,[2] Gianluca Ciancaleoni,[2] Massimo Guelfi,[2] Fabio Marchetti,[2] Franco Scalambra[1], Luísa M. D. R. S. Martins[3]* and Antonio Romerosa[1]*

[1]Área de Química Inorgánica-CIESOL, Universidad de Almería, Almería, Spain.

[2] Department of Chemistry and Industrial Chemistry, University of Pisa, Via Giuseppe Moruzzi 13, I-56124 Pisa, Italy.

[3] Centro de Química Estrutural, Departamento de Engenharia Química, Instituto Superior Técnico, Universidade de Lisboa, Av. Rovisco Pais 1, 1000-049 Lisboa, Portugal.

Table of Contents

1. Synthesis and characterization of Ru-C-scorpionate complexes
    - **1.1.**[Ru(κ^3-Tpm)(NCMe)$_3$(NO$_3$)$_2$ (**1**)
    - **1.2.**[Ru(κ^3-Tpm)(PPh$_3$)(NCMe)$_2$](NO$_3$)$_2$ (**3**)
    - **1.3.**[RuCl(κ^3-Tpm)(PPh$_3$)(κ*N*-NH$_2$CH$_2$CH$_2$OH)]Cl (**6**)
2. Catalytic cycloaddition of benzyl azide and the alkynes, phenylacetylene and diphenylacetlyne, mediated by ruthenium complexes
3. Computational studies
4. Reactivity of complex [Ru(κ^3-Tpm)(PPh$_3$)(NCMe)$_2$](NO$_3$)$_2$ (**3**) along cycloaddition of phenyl azide and phenylacetylene in dimethylformamide
5. References

1. Synthesis and characterization of ruthenium C-complexes

1.1. [Ru(κ^3-Tpm)(NCMe)$_3$](NO$_3$)$_2$ (1)

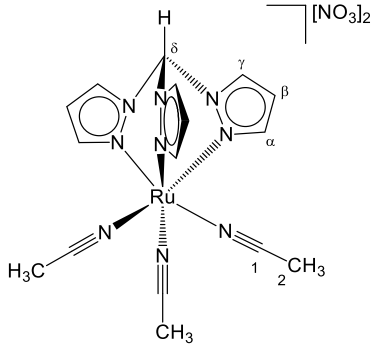

FigureS1. Structure of **1** (labelling refers to carbon atoms).

A mixture of RuCl$_3$(κ^3-Tpm)·1.5H$_2$O (200 mg, 0.464 mmol) and AgNO$_3$ (238 mg, 1.40 mmol) in 40 mL of acetonitrile/water mixture (v/v 3:1) was heated at reflux for 8h under light protection. After cooling to room temperature, the mixture was filtered through celite, and the solvent evaporated under reduced pressure. The crude product was redissolved in the minimum volume of water and filtered through celite. The volatiles were evaporated under reduced pressure. The obtained powder was dissolved in the minimum volume of acetone/MeOH and precipitated with diethyl ether. The resulting mixture was filtered, and the isolated solid was dried under vacuum. Purple solid, yield 188 mg (72 %). Anal. calcd. for C$_{16}$H$_{19}$N$_{11}$O$_6$Ru: C, 34.17; H, 3.40; N, 27.39. Found: C, 34.15; H, 3.32; N, 27.42. IR (solid state): $\tilde{\upsilon}$/cm^{-1} = 3131w, 3109w, 2990w, 2933w, 1909m, 1661m, 1514m, 1406-1250s ($\tilde{\upsilon}_{NO3}$), 1091m, 1057m, 1036m, 993m, 859m, 822w, 779s, 757s. ^{1}H NMR (CD$_3$OD): δ/ppm = 9.66 (s, 1H, C^{δ}H); 8.46 (d, 3H, $^3J_{HH}$ = 2.8 Hz, C^{γ}H); 8.24 (d-br, 3H, C^{a}H); 6.68 (t-br, 3H, $^3J_{HH}$ = 2.6 Hz C^{β}H); 2.50 (s, 9H, C^{2}H). ^{13}C NMR (CD$_3$OD): 148.6 (C^a); 136.2 (C^γ); 127.7 (C^1); 110.3 (C^β); 3.3 (C^2). ^{1}H NMR (D$_2$O): δ/ppm = 9.44 (s, 1H, C^{δ}H); 8.32 (d, 3H, $^3J_{HH}$ = 3.0 Hz, C^{γ}H); 8.11 (d, 3H, $^3J_{HH}$ = 2.3 Hz, C^{a}H); 6.59 (t, 3H, $^3J_{HH}$ = 2.3 Hz, C^{β}H); 2.50 (s, 9H, C^{2}H).

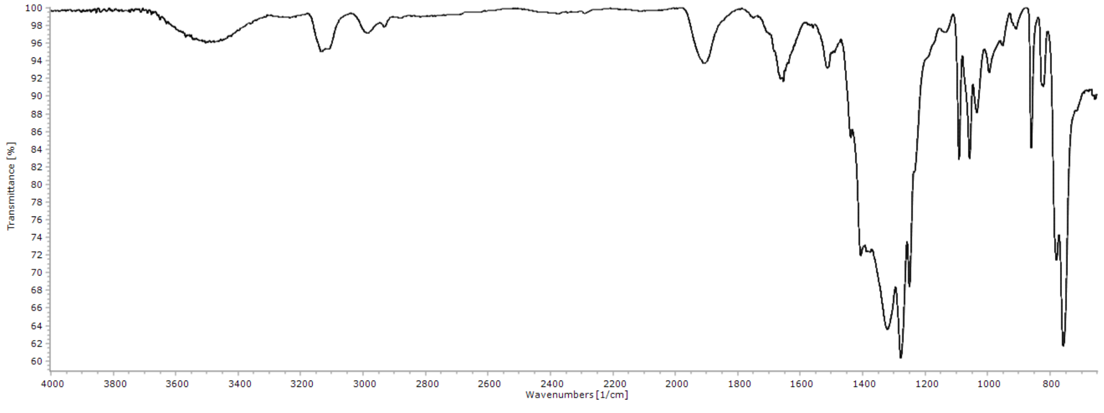

FigureS2. Solid-state IR spectrum (650-4000 cm^{-1}) of **1**.

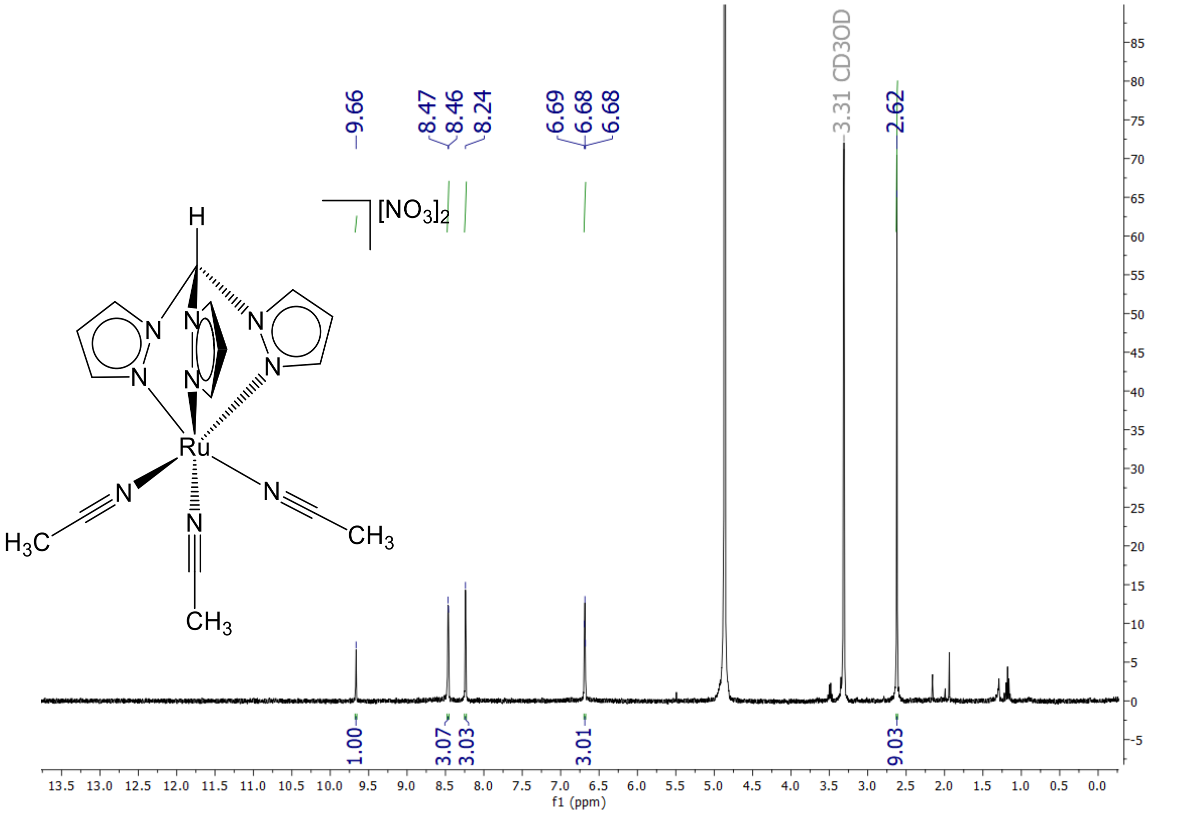

FigureS3. ^{1}H NMR spectrum (401 MHz, CD_3OD) of **1**.

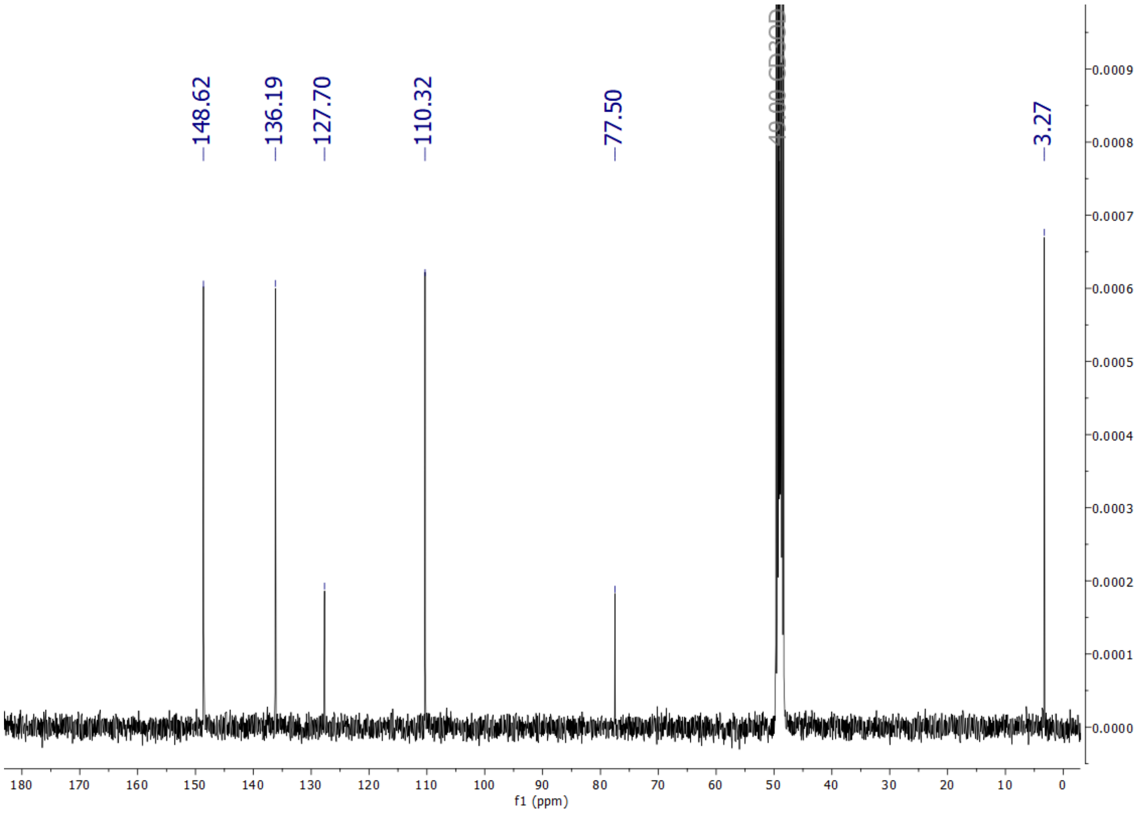

FigureS4. ^{13}C NMR spectrum (101 MHz, CD_3OD) of **1**.

1.2. $[Ru(\kappa^3\text{-}Tpm)(PPh_3)(NCMe)_2](NO_3)_2$ (3)[1]

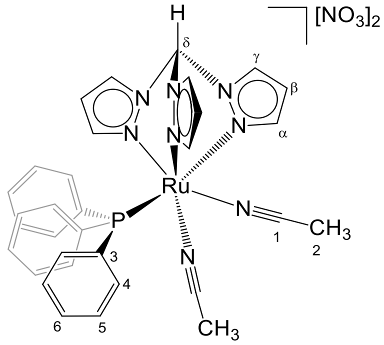

FigureS5. Structure of **3** (labelling refers to carbon atoms).

A mixture of $[RuCl(\kappa^3\text{-}Tpm)(PPh_3)_2]$ (100 mg, 0.110 mmol) and $AgNO_3$ (37 mg, 0.22 mmol) in 10 mL of acetonitrile (MeCN) was heated at reflux for 16 h. After cooling to room temperature, the mixture was filtered through celite, and the solvent evaporated under reduced pressure. The crude product was redissolved in the minimum volume of dichloromethane, filtered on celite and the volatiles evaporated under reduced pressure. The obtained powder was dispersed in THF and left stirring for 1h. The solid was eventually filtered, washed with diethyl ether, and dried under vacuum. White solid, yield 85 mg (98%). Anal. calcd. for $C_{32}H_{31}N_{10}O_6PRu$: C, 49.04; H, 3.99; N, 17.87. Found: C, 49.11; H, 3.95; N, 17.93. IR (solid state): $\tilde{\upsilon}/cm^{-1}$ = 3153w, 3128w, 3108w, 2980w, 2955w, 2924w, 1740w, 1513w, 1479w, 1457w, 1431ww, 1400m, 1374m (NO_3), 1315s, 1250m, 1227m, 1182w, 1088m, 1051m, 994w, 861m, 827m, 779s, 747s, 696s. ^1H NMR (CD_3OD): δ/ppm = 9.88 (s, 1H, $C^{\delta}H$); 8.55 (d, 2H, $^3J_{HH}$ = 2.9 Hz, $C^{\gamma}H$); 8.50 (d, 1H, $^3J_{HH}$ = 2.8 Hz, $C^{\gamma}H$); 8.46 (d, 1H, $^3J_{HH}$ = 2.2 Hz, $C^{\alpha}H$); 7.04 (d, 2H, $^3J_{HH}$ = 2.3 Hz, $C^{\alpha}H$); 7.57 (m, 3H, C^6H); 7.47 (m, 6H, C^4H or C^5H); 7.17 (m, 6H, C^4H or C^5H); 6.72 (t-br, 1H, $C^{\beta}H$); 6.39 (t, 2H, $^3J_{HH}$ = 2.6 Hz, $C^{\beta}H$); 2.51 (s, 6H, C^2H). ^{13}C NMR (CD_3OD): δ/ppm = 149.7 ($2C^{\alpha}$); 146.8 ($C^{\alpha}H$); 137.3 ($2C^{\gamma}$); 135.4 (C^{γ}); 134.9 (d, $^2J_{CP}$ = 9.8 Hz, C^4); 132.3 (d, $^4J_{CP}$ = 2.4 Hz, C^6); 131.1 (d, $^1J_{CP}$ = 44.6 Hz, C^3); 130.1 (d, $^3J_{CP}$ = 9.7 Hz, C^5); 129.3 (C^1); 110.35 ($3C^{\beta}$); 3.76 (C^2). $^{31}P\{^1H\}$ NMR (CD_3OD): δ/ppm = 46.9. ^1H NMR ($CDCl_3$): δ/ppm = 11.31 (s, 1H, $C^{\delta}H$); 8.83 (d-br, 1H, $C^{\alpha}H$); 6.75 (d, 2H, $^3J_{HH}$ = 2.3 Hz, $C^{\alpha}H$); 8.50 (d, 2H, $^3J_{HH}$ = 2.3 Hz, $C^{\gamma}H$); 8.47 (d, 1H, $^3J_{HH}$ = 2.8 Hz, $C^{\gamma}H$); 7.51 (t-br, 3H, C^6H); 7.40 (t, 6H, $^3J_{HH}$ = 7.9 Hz, C^4H or C^5H); 7.10 (t, 6H, $^3J_{HH}$ = 7.8 Hz, C^4H or C^5H); 6.59 (t, 1H, $^3J_{HH}$ = 2.6 Hz, $C^{\beta}H$); 6.18 (t, 2H, $^3J_{HH}$ = 2.6 Hz, $C^{\beta}H$); 2.56 (s, 6H, C^2H). $^{31}P\{^1H\}$ NMR ($CDCl_3$): δ/ppm = 47.9.

1.3. [RuCl(κ^3-Tpm)(PPh_3)(k*N*-$NH_2CH_2CH_2OH$)]Cl (6)[1]

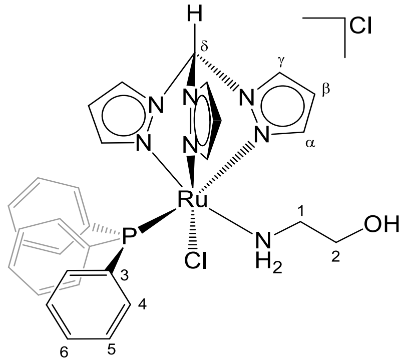

FigureS6. Structure of **6** (labelling refers to carbon atoms).

A solution of [RuCl(κ^3-Tpm)(PPh_3)$_2$] (120 mg, 0,132 mmol) and ethanolamine (17 μL, 0.27 mmol) in THF (8 mL) was heated at reflux for 16 h. The product precipitated from the reaction medium as a fine yellow powder. After cooling to room temperature, the solid was filtered, washed with THF and diethyl ether, and dried under vacuum. Yellow solid, yield 92 mg (98%). Anal. calcd. for $C_{30}H_{32}Cl_2N_7OPRu$: C, 50.78; H, 4.55; Cl, 9.99; N, 13.82. Found: C, 50.65; H, 4.44; Cl, 9.94; N, 13.85. IR (solid state): $\tilde{\upsilon}$/cm^{-1} = 3405w (OH), 3295w (NH), 3106w, 3070w, 2984w, 2969w, 2899w, 2872w, 1508w, 1481w, 1453w, 1434m, 1405w, 1289m, 1273w, 1254m, 1227w, 1187w, 1088s, 1068m, 1050m, 854m, 794m, 768s, 761s, 743s, 694s, 683m, 613w, 607w, 530s, 511s, 501s, 452m, 421w. ^{1}H NMR (CD_3OD): δ/ppm = 9.63 (s, 1H, $C^{\delta}H$); 8.46, 8.44, 8.42 (d, 3H, $^3J_{HH}$ = 2.9 Hz, $C^{\gamma}H$); 8.34, 7.28, 7.05 (d, 3H, $^3J_{HH}$ = 2.2 Hz, $C^{\alpha}H$); 7.48 (m, 3H, C^6H); 7.40-7.32 (m, 12H, $C^4H + C^5H$); 6.68, 6.31, 6.21 (t, 1H, $^3J_{HH}$ = 2.6 Hz, $C^{\beta}H$); 3.37 (t-br, 1H, NH_2); 3.28 (m, 2H, C^2H); 2.70 (t-br, 1H, NH_2); 2.39 (m, 1H, C^1H); 1.99 (m, 1H, C^1H). *OH not observed.* ^{31}P NMR (CD_3OD): δ/ppm = 53.0. ^{13}C{^{1}H} NMR (CD_3OD): δ/ppm = 151.5, 150.1, 146.4 (C^{α}); 136.1, 135.9, 134.7 (C^{δ}); 134.9 (d, $^2J_{CP}$ = 9.4 Hz, C^4); 133.5 (d, $^1J_{CP}$ = 40.1 Hz, C^3); 131.4 (d, $^4J_{CP}$ = 1.8 Hz, C^6); 129.8 (d, $^3J_{CP}$ = 9.2 Hz, C^5); 109.8, 109.7, 109.6 ($C^{\beta}H$); 62.43 (C^2H); 48.3 (C^1H).

2. Catalytic cycloaddition of benzyl azide and the alkynes, phenylacetylene and diphenylacetlyne, mediated by ruthenium complexes

TableS1. Catalytic cycloaddition of benzyl azide (**I**) and phenylacetylene (**II**) into 1-benzyl-5-phenyl-1H-1,2,3-triazole (**III**) and 1-benzyl-4-phenyl-1H-1,2,3-triazole (**IV**) mediated by C-scorpionate ruthenium(II) complexes in several solvents.

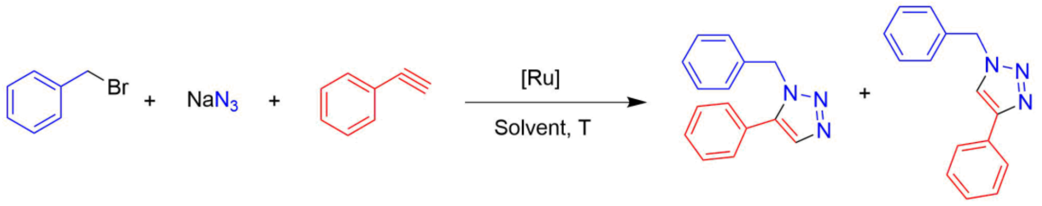

I' I" II III IV

Catalyst	Solvent	T (°C)	Conversion [a] (%) Isomer 1,5 III	Isomer 1,4 IV	Ratio III/IV	Conv. [a] (%)
**1**	H_2O	100	33	24	1.4:1	57
**1**	H_2O/acetonitrile	85	26	23	1.1:1	49
**1**	MeOH	65	16	16	1:1	32
**1**	DCM	--	--	--	--	--
**1**	DMF	100	49	28	1.8:1	77
**2**	H_2O	100	29	27	1.1:1	56
**2**	H_2O/acetonitrile	85	54	46	1.2:1	99
**2**	MeOH	65	49	49	1:1	98
**2**	DCM	40	10	11	1.1:1	21
**2**	DMF	100	52	44	1.2:1	96
**3**	H_2O	100	--	---	--	--
**3**	H_2O/acetonitrile	85	51	49	1.04:1	99
**3**	MeOH	65	48	52	1:1	99
**3**	DCM	40	10	13	1.3:1	23
**3**	DMF	100	55	39	1.4:1	94
**4**	H_2O	100	30	30	1:1	60
**4**	H_2O/acetonitrile	85	39	34	1.2:1	73

**4**	MeOH	65	48	52	1:1.1	99
**4**	DCM	40	2	2	1:1	4
**4**	DMF	100	59	38	1.6:1	96
**5**	H_2O	85	39	35	1.1:1	74
**5**	H_2O/acetonitrile	100	46	44	1.2:1	90
**5**	MeOH	65	46	54	1:1.2	99
**5**	DCM	40	33	20	1.7:1	80[b]
**5**	DMF	100	56	37	1.5:1	93
**6**	H_2O	100	--	--	--	--
**6**	H_2O/acetonitrile	85	51	44	1.2:1	95
**6**	MeOH	65	48	52	1:1.1	99
**6**	DCM	40	7	18	2.5:1	93 [b]
**6**	DMF	100	58	34	1.7:1	92

1 mol% [Ru], proportion of H_2O/acetonitrile 1:1, 3 mL solvent, 6 h, benzyl azide (**I**) was formed *in situ* by reaction of phenyl bromide (**I'**) and NaN_3 (**II''**) in a ratio 1:1, azide/alkyne (**I/II**) = 1:1.25, [**I**] = 0.11 M, [**II**] = 0.14 M, [a] conversion (%) obtained by ^{1}H NMR; average of 3 experiments (standard deviation). [b] Total conversion (%) includes other byproducts not elucidated.

TableS2. Catalytic cycloaddition of for the cycloaddition of benzyl azide (**I**) and phenyl acetylene (**II**) into 1-benzyl-5-phenyl-1H-1,2,3-triazole (**III**) and 1-benzyl-4-phenyl-1H-1,2,3-triazole (**IV**) mediated by piano-stool ruthenium(II) complexes (**7-10**) in several solvents.

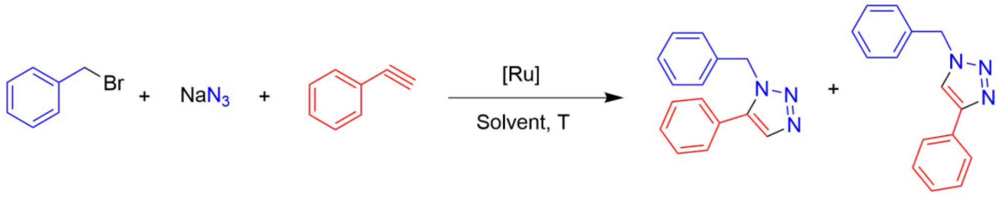

I' I'' II III IV

**Catalyst**	**Ratio Azide/alkyne I/II**	**T (°C)**	**Conversion [a] (%)**			**Conv. [a] (%)**
			**Isomer 1,5 (III)**	**Isomer 1,4 (IV)**	**Ratio III/IV**	
**7**	H_2O	100	52	46	1.1:1	98
**7**	H_2O/acetonitrile	85	56	41	1.4:1	97
**7**	MeOH	65	47	53	1:1.1	99
**7**	DMF	100	51	41	1.2:1	92

**8**	H_2O	100	48	48	1:1	96
**8**	H_2O/acetonitrile	85	48	47	1.02:1	95
**8**	MeOH	65	43	46	1:1.07	89
**8**	DMF	100	51	31	1.7:1	82
**9**	H_2O	100	43	43	1:1	86
**9**	H_2O/acetonitrile	85	47	43	1.1:1	90
**9**	MeOH	65	44	50	1:1.1	94
**9**	DMF	100	41	24	1.7:1	65
**10**	H_2O	100	39	36	1.1:1	75
**10**	H_2O/acetonitrile	85	39	34	1.2:1	73
**10**	MeOH	65	39	43	1:1.1	82
**10**	DMF	100	23	42	1.8:1	65

1 mol% [Ru], 3 mL solvent, 6 h, benzyl azide (**I**) was formed *in situ* by reaction of phenyl bromide (**I'**) and NaN_3 (**I''**) in a ratio 1:1, azide/alkyne (**I**/**II**) = 1:1.25, [**I**] = 0.11 M, [**II**] = 0.14 M, [a] conversion (%) obtained by ^{1}H NMR; average of 3 experiments (standard deviation)..

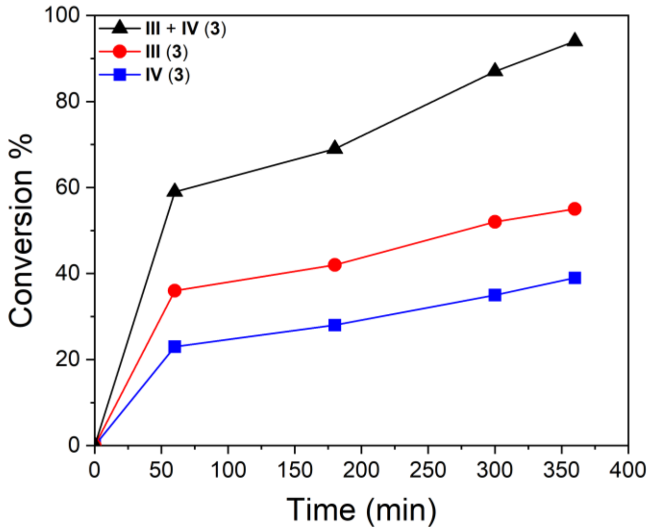

FigureS7. Catalytic cycloaddition of benzyl azide (**I**) and phenylacetylene (**II**) into 1-benzyl-5-phenyl-1H-1,2,3-triazole (**III**) and 1-benzyl-4-phenyl-1H-1,2,3-triazole (**IV**) mediated by **3** *vs.* time in DMF. Conditions: 1 mol% **3**, 3 mL DMF, **I** was formed *in situ* by reaction of **I'** and **I''** in a ratio 1:1, **I**/**II** = 1:1.25, [**I**] = 0.11 M, [**II**] = 0.14 M, 3 mL DMF, conversion (%) obtained by ^{1}H NMR, average of 3 experiments.

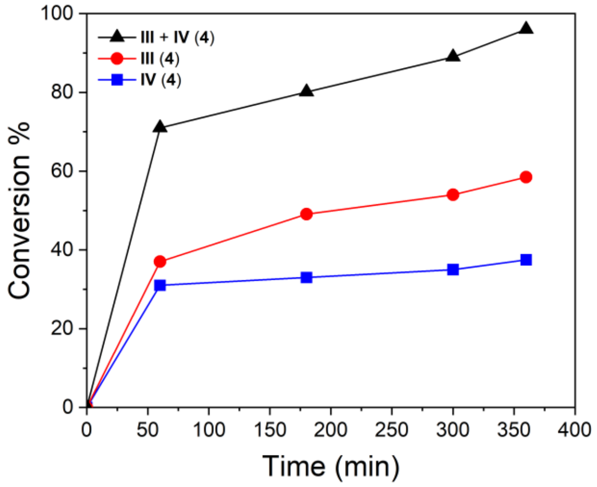

FigureS8. Catalytic cycloaddition of benzyl azide (**I**) and phenylacetylene (**II**) into 1-benzyl-5-phenyl-1H-1,2,3-triazole (**III**) and 1-benzyl-4-phenyl-1H-1,2,3-triazole (**IV**) mediated by **4** *vs.* time in DMF. Conditions: 1 mol% **4**, 3 mL DMF, **I** was formed *in situ* by reaction of **I'** and **I''** in a ratio 1:1, **I/II** = 1:1.25, [**I**] = 0.11 M, [**II**] = 0.14 M, conversion (%) obtained by ^{1}H NMR, average of 3 experiments (standard deviation).

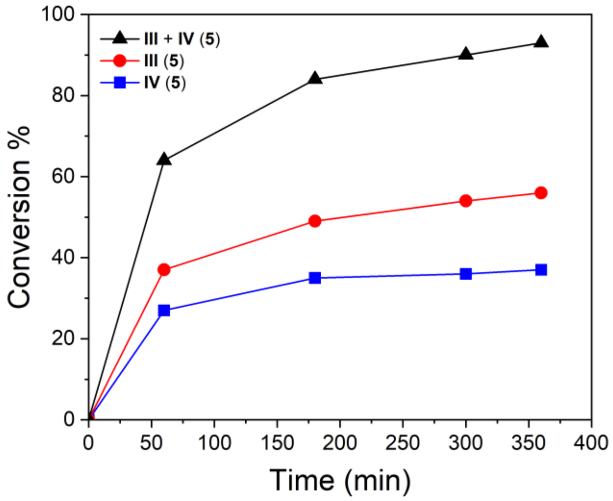

FigureS9. Catalytic cycloaddition of benzyl azide (**I**) and phenylacetylene (**II**) into 1-benzyl-5-phenyl-1H-1,2,3-triazole (**III**) and 1-benzyl-4-phenyl-1H-1,2,3-triazole (**IV**) mediated by **5** *vs.* time in DMF. Conditions: 1 mol% **5**, 3 mL DMF, **I** was formed *in situ* by reaction of **I'** and **I''** in a ratio 1:1, **I/II** = 1:1.25, [**I**] = 0.11 M, [**II**] = 0.14 M, conversion (%) obtained by ^{1}H NMR, average of 3 experiments (standard deviation).

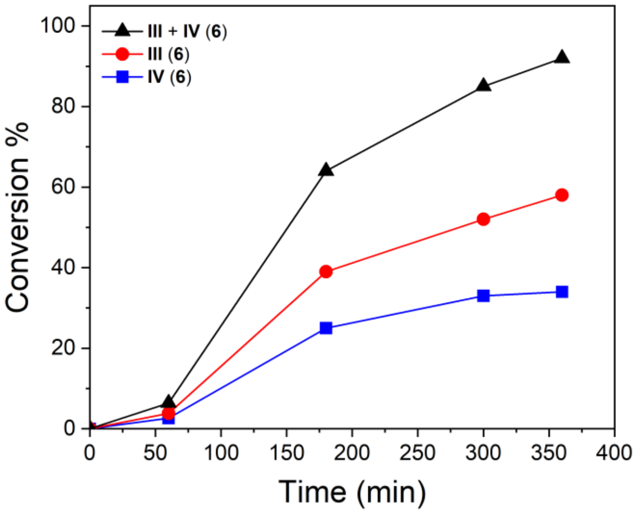

FigureS10. Catalytic cycloaddition of benzyl azide (**I**) and phenylacetylene (**II**) into 1-benzyl-5-phenyl-1H-1,2,3-triazole (**III**) and 1-benzyl-4-phenyl-1H-1,2,3-triazole (**IV**) mediated by **6** *vs.* time in DMF. Conditions: 1 mol% **6**, 3 mL DMF, **I** was formed *in situ* by reaction of **I'** and **I''** in a ratio 1:1, **I/II** = 1:1.25, [**I**] = 0.11 M, [**II**] = 0.14 M, conversion (%) obtained by ^{1}H NMR, average of 3 experiments (standard deviation).

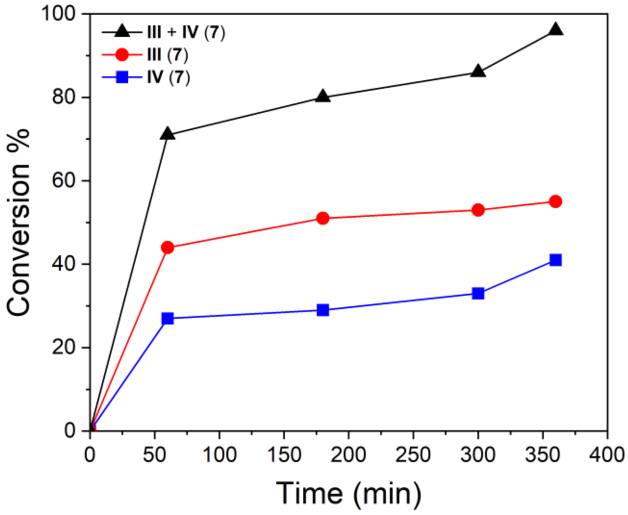

FigureS11. Catalytic cycloaddition of benzyl azide (**I**) and phenylacetylene (**II**) into 1-benzyl-5-phenyl-1H-1,2,3-triazole (**III**) and 1-benzyl-4-phenyl-1H-1,2,3-triazole (**IV**) mediated by **7** *vs.* time in of H_2O/acetonitrile 1:1. Conditions: 1 mol% of **7**, 3 mL of H_2O/acetonitrile 1:1, **I** was formed *in situ* by reaction of **I'** and **I''** in a ratio 1:1, **I/II** = 1:1.25, [**I**] = 0.11 M, [**II**] = 0.14 M, conversion (%) obtained by ^{1}H NMR, average of 3 experiments (standard deviation).

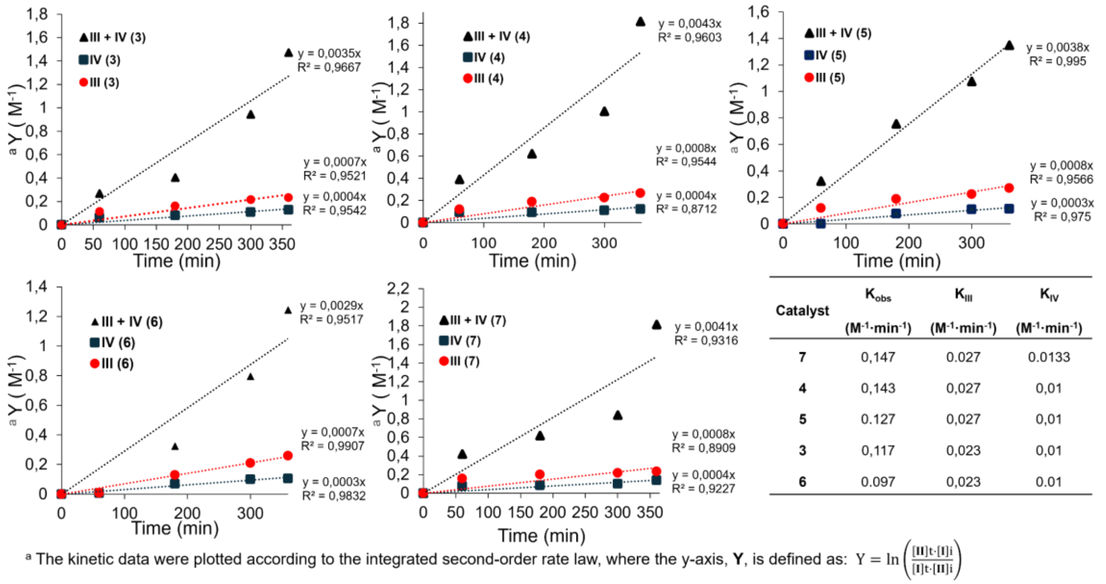

Catalyst	K_{obs} ($M^{-1}\cdot min^{-1}$)	K_{III} ($M^{-1}\cdot min^{-1}$)	K_{IV} ($M^{-1}\cdot min^{-1}$)
7	0,147	0.027	0.0133
4	0,143	0,027	0,01
5	0.127	0,027	0,01
3	0,117	0,023	0,01
6	0.097	0,023	0.01

[a] The kinetic data were plotted according to the integrated second-order rate law, where the y-axis, **Y**, is defined as: $Y = \ln\left(\frac{[II]t\cdot[I]i}{[I]t\cdot[II]i}\right)$

Figure S12. Data fitting and rate determination for a second-order catalytic cycloaddition reaction (K_{obs}) of benzyl azide (**I**) and phenylacetylene (**II**) into 1-benzyl-5-phenyl-1H-1,2,3-triazole (**III**) and 1-benzyl-4-phenyl-1H-1,2,3-triazole (**IV**) mediated by **3-6** (in DMF) and **7** (in water/acetonitrile 1:1) and individual rate constants (K_{III} and K_{IV}).

TableS3. Catalytic cycloaddition of benzyl azide (**I**) and phenylacetylene (**II**) into 1-benzyl-5-phenyl-1H-1,2,3-triazole (**III**) and 1-benzyl-4-phenyl-1H-1,2,3-triazole (**IV**) mediated by C-scorpionate ruthenium(II) complexes varying substrate concentrations in DMF or MeOH.

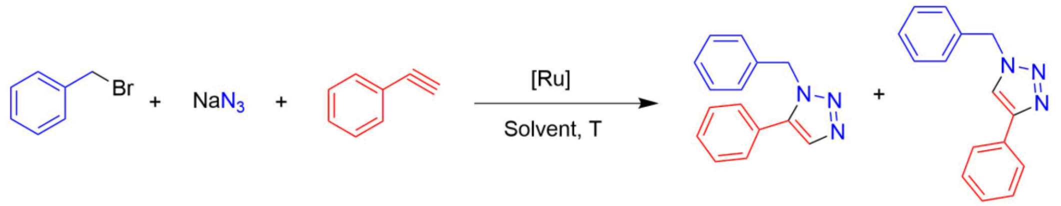

I' I'' II III IV

Catalyst	Solvent	[I] M	[II] M	T (°C)	Conversion [a] %			Conv. [a] (%)
					Isomer 1,5 (III)	Isomer 1,4 (IV)	Ratio III/IV	
**3**	MeOH	0.11	0.14	65	48 ± 0.5	52 ± 0.1	1:1	99 ± 0.4
**3**	MeOH	0.55	0.7	65	39 ± 1.6	39 ± 2.3	1:1	78 ± 3.9
**3**	DMF	0.11	0.14	100	55 ± 2.3	39 ± 3.2	1.4:1	94 ± 1.0
**3**	DMF	0.55	0.7	100	63 ± 1.7	26 ± 1.1	2.4:1	89 ± 2.7
**3**	DMF[b]	0.55	0.7	100	76 ± 1.5	17 ± 1.1	4.5:1	93 ± 2.6
**4**	MeOH	0.11	0.14	65	48 ± 0.4	52 ± 1.0	1:1.1	99 ± 0.6

**4**	MeOH	0.55	0.7	65	6 ± 5.4	6 ± 4.9	1:1	12±10.3
**4**	DMF	0.11	0.14	100	59 ± 1.5	38 ± 2.7	1.6:1	96 ± 4.0
**4**	DMF	0.55	0.7	100	59 ± 0.5	41 ± 1.4	1.5:1	99 ± 1.1
**4**	DMF[b]	0.55	0.7	100	72 ± 1.3	20 ± 1.3	3.6:1	92 ± 2.6
**5**	MeOH	0.11	0.14	65	46 ± 0.6	54 ± 0.6	1:1.2	99 ± 1.0
**5**	MeOH	0.55	0.7	65	22 ± 4.2	24 ± 2.7	1:1.1	46 ± 6.6
**5**	DMF	0.11	0.14	100	56 ± 1.0	37 ± 3.3	1.5:1	93 ± 3.5
**5**	DMF	0.55	56	100	49 ± 1.4	31 ± 0.8	1.6:1	80 ± 2.2
**6**	MeOH	0.11	0.14	65	48 ± 0.8	52 ± 0.6	1:1.1	99 ± 0.5
**6**	MeOH	0.55	0.7	65	42 ± 1.6	46 ± 0.9	1:1.1	88 ± 1.1
**6**	DMF	0.11	0.14	100	58 ± 1.8	34 ± 1.9	1.7:1	92 ± 3.5
**6**	DMF	0.55	0.7	100	38 ± 1.4	24 ± 1.1	1.6:1	62 ± 2.4

1 mol% [Ru], 6 h, **I** was formed *in situ* by reaction of **I’** and **II’** in a ratio 1:1, [a] conversion (%) obtained by ^{1}H NMR, **I/II** = 1:1.25, average of 3 experiments (standard deviation). [b] Without stirring.

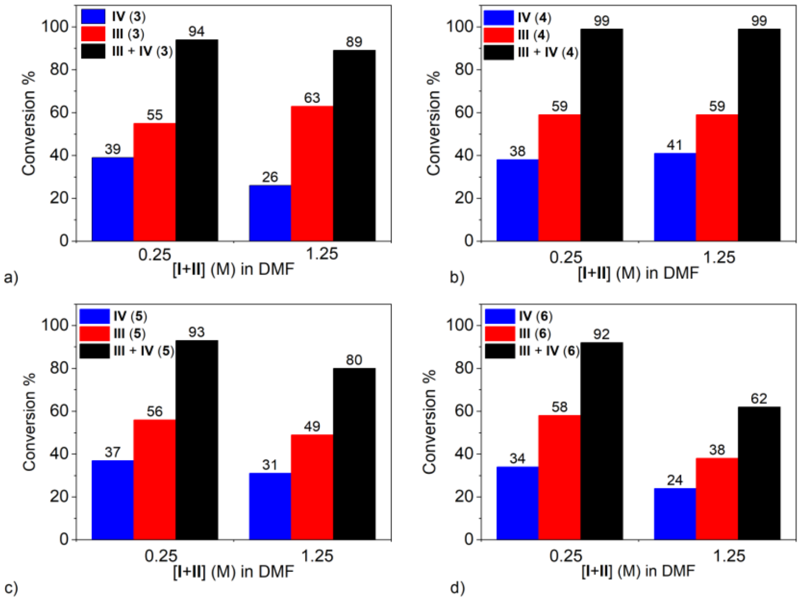

Figure S13. Catalytic cycloaddition of benzyl azide (**I**) and phenylacetylene (**II**) into 1-benzyl-5-phenyl-1H-1,2,3-triazole (**III**) and 1-benzyl-4-phenyl-1H-1,2,3-triazole (**IV**) mediated by a) **3**, b) **4**, c) **5** and d) **6**, depending on the concentration of **I** and **II** in DMF. Conditions: 1 mol% [Ru], 6 h, 3 or 0.6 mL DMF, **I** was formed *in situ* by reaction of **I’** and **II’** in a ratio 1:1, **I/II** = 1:1.25, [**I+II**] = 0.25 M ([**I**] = 0.11 M, [**II**] = 0.14 M); and [**I+II**] = 1.25 M ([**I**] = 0.55 M, [**II**] = 0.7 M), conversion (%) obtained by ^{1}H NMR, average of 3 experiments.

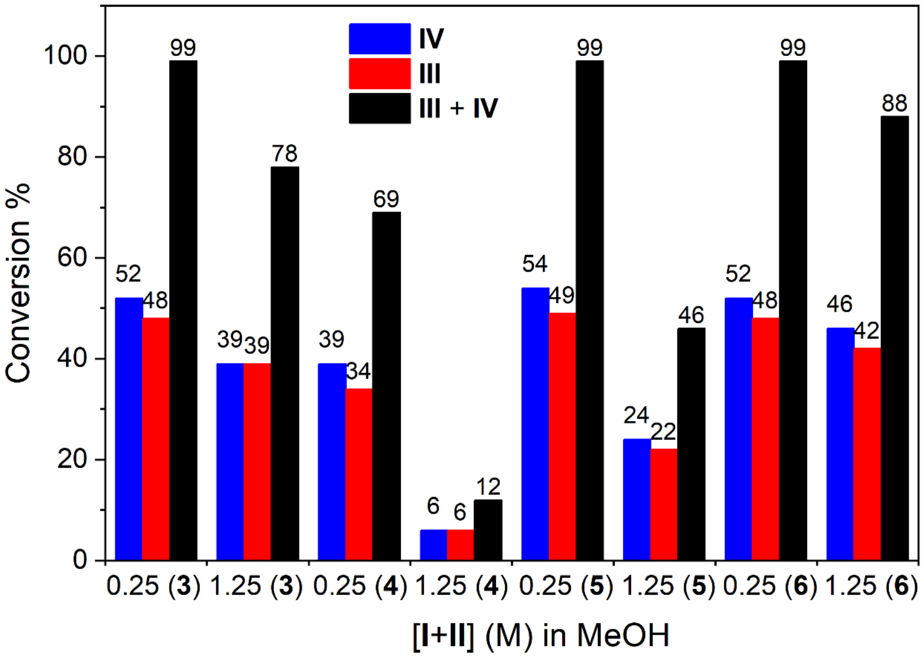

Figure S14. Catalytic cycloaddition of benzyl azide (**I**) and phenylacetylene (**II**) into 1-benzyl-5-phenyl-1H-1,2,3-triazole (**III**) and 1-benzyl-4-phenyl-1H-1,2,3-triazole (**IV**) mediated by complexes **3-6** depending on the concentration of **II** in MeOH. Conditions: 1 mol% [Ru], 6 h, 3 or 0.6 mL MeOH, **I** was formed *in situ* by reaction of **I'** and **I''** in a ratio 1:1, **I/II** = 1:1.25, [**I+II**] = 0.25 M ([**I**] = 0.11 M, [**II**] = 0.14 M); and [**I+II**] = 1.25 M ([**I**] = 0.55 M, [**II**] = 0.7 M), conversion (%) obtained by NMR, average of 3 experiments (standard deviation).

Table S4. Catalytic cycloaddition of benzyl azide (**I**) and diphenylacetylene (**V**) into 1-benzyl-4,5-diphenyl-1*H*-1,2,3-triazole (**VI**) mediated by complexes **3** in DMF and **7** in H_2O/acetonitrile (1:1).

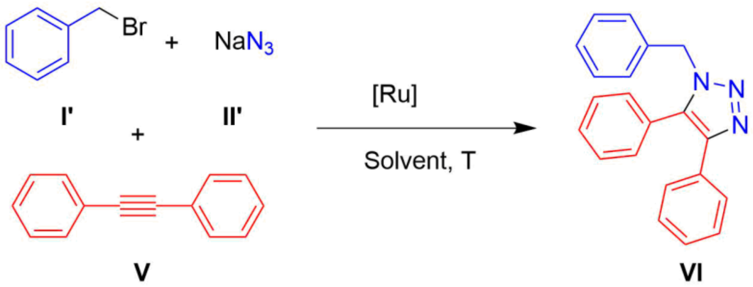

Catalyst	Solvent	[I] M	[V] M	T (ºC)	Conversion VI % [a]
**3**	DMF	0.55	0.7	100	75 ± 2.5
**7**	H_2O/acetonitrile	0.11	0.14	85	6 ± 1.6

1 mol% **1**, 6 h, 3 and 0.6 mL solvent, benzyl azide (**I**) was formed *in situ* by reaction of phenyl bromide (**I'**) and NaN_3 (**I''**) in a ratio 1:1, azide/alkyne (**I/V**) = 1:1.25, [a] conversion (%) obtained by NMR, average of 3 experiments (standard deviation).

3. Computational studies

Table S5. EDA results (in kcal/mol).

Fragmentation	$Cp^{-}\cdots[RuCl(NCCMe)(PH_3)]^{+}$	$Tpm\cdots[RuCl(NCCMe)(PH_3)]^{+}$
**E_{int}**	-215	-107
**E_o**	-230	-180
**E_{el}**	-244	-158
**E_{Pauli}**	400	370
**E_{disp}**	-9	-20
**$\Delta\rho_0$ (σ donation)**	-68	-48
**$\Delta\rho_1$ (σ donation)**	-69	-41
**$\Delta\rho_2$ (π back-donation)**	-17	-15
**$\Delta\rho_3$ (π back-donation)**	-20	-13
**$\Delta\rho_4$ (σ donation)**	-16	-23

E_{int} = interaction energy; E_o = orbital energy; E_{el} = electrostatic energy; E_{Pauli} = Pauli energy; E_{disp} = dispersion contribution; $\Delta\rho_i$ = i-th NOCV component of the orbital energy.

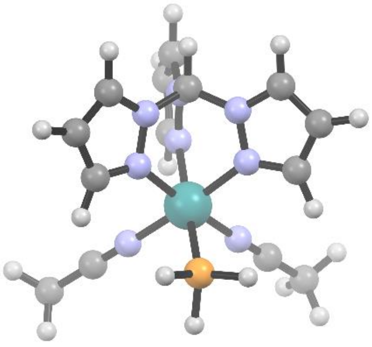

3'

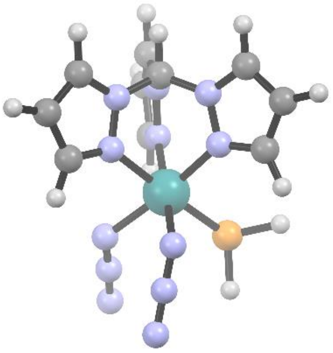

3'a

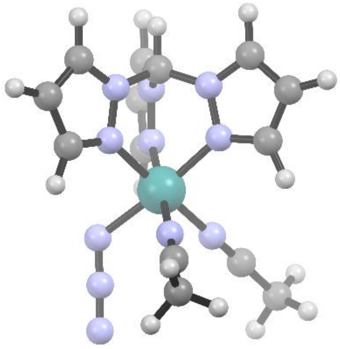

3'b

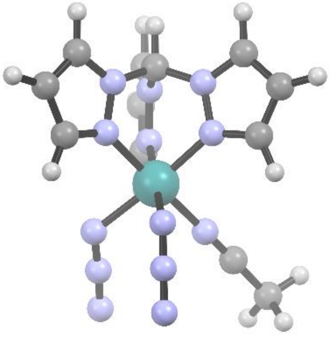

3’c

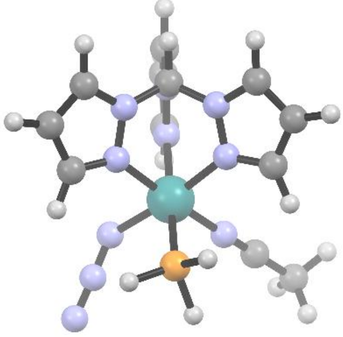

3’d

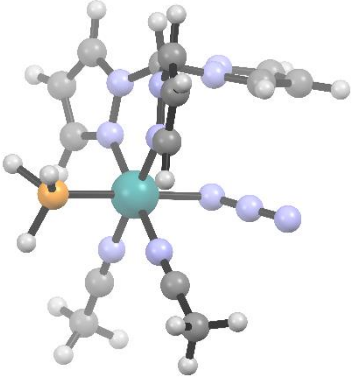

3’e

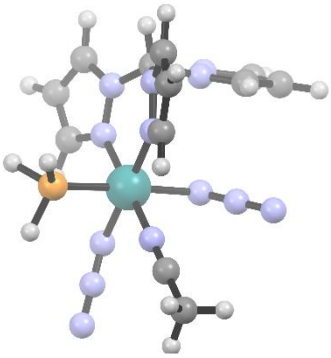

3’f

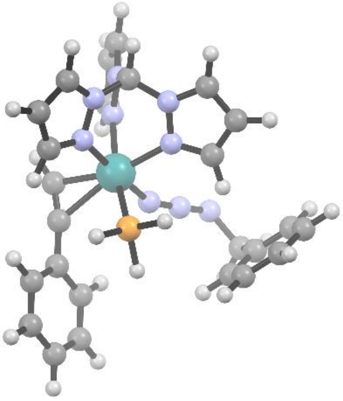

3’g

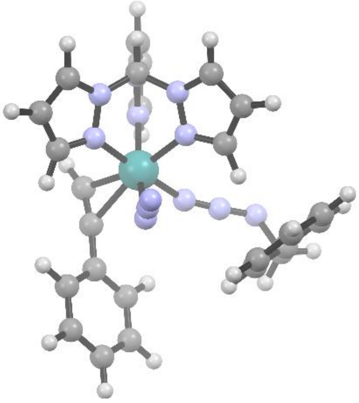

3’h

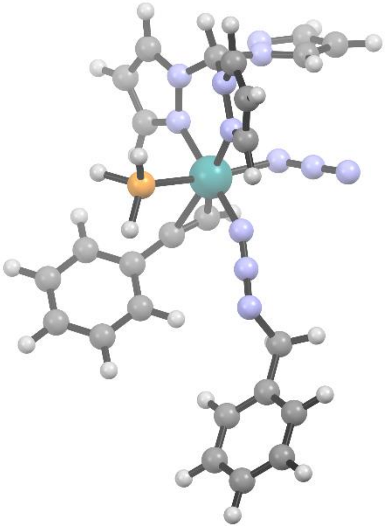

3’i

Figure S15. DFT optimized geometries for speciation of **3’**.

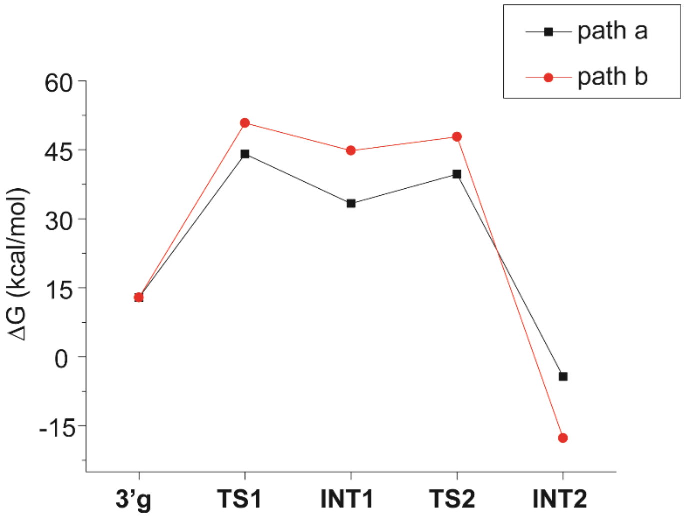

Figure S16. Energy path for the reaction starting from **3'g**.

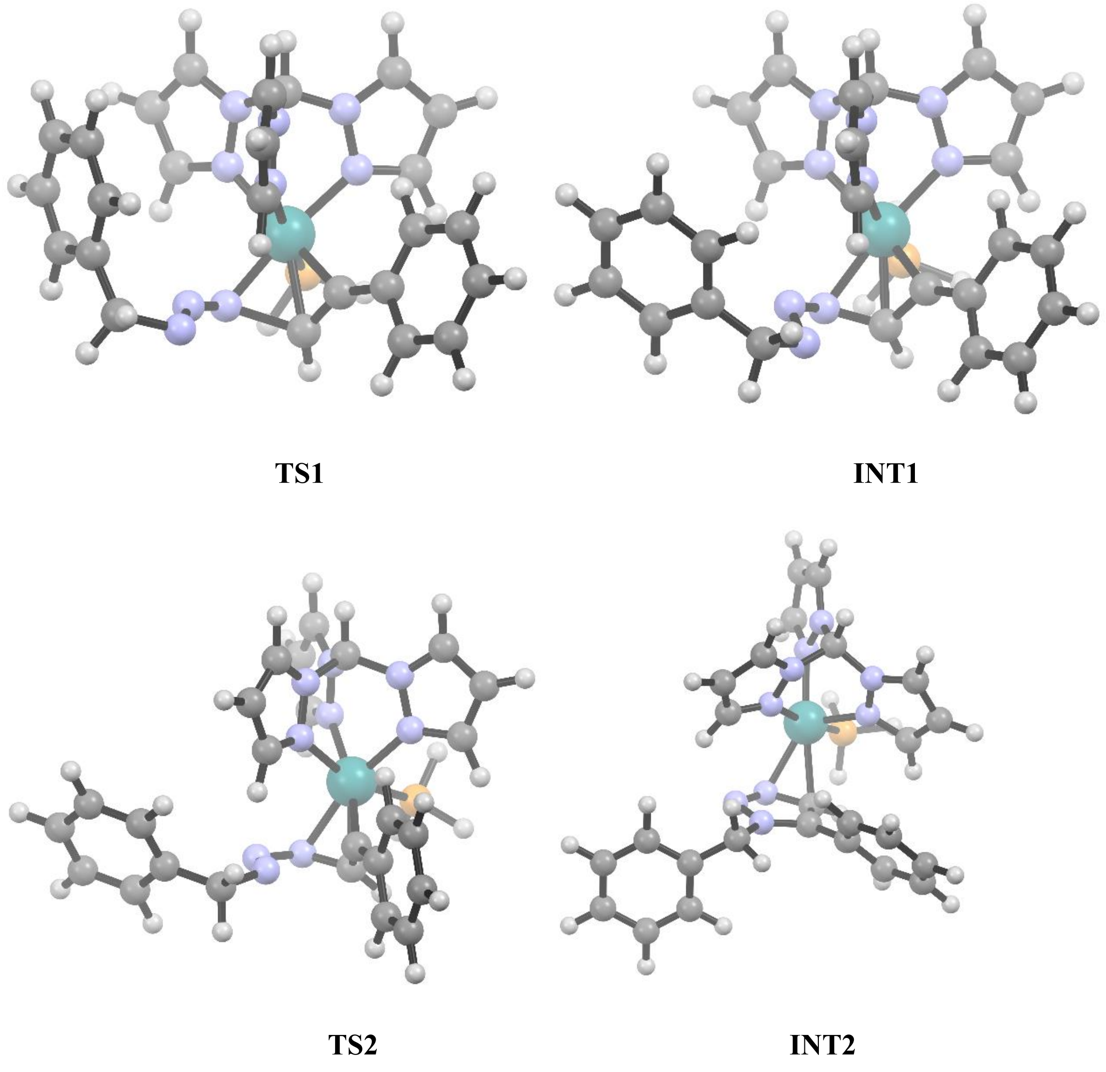

Figure S17. DFT-optimized geometries for the reaction starting from **3'g** (path a).

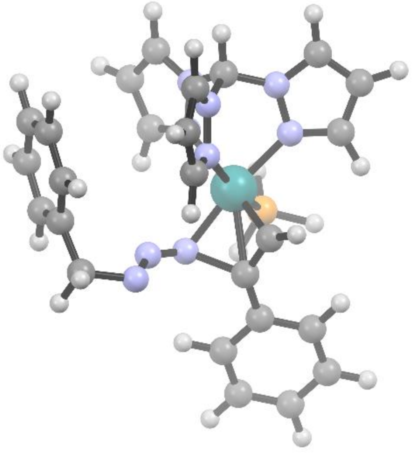

TS1

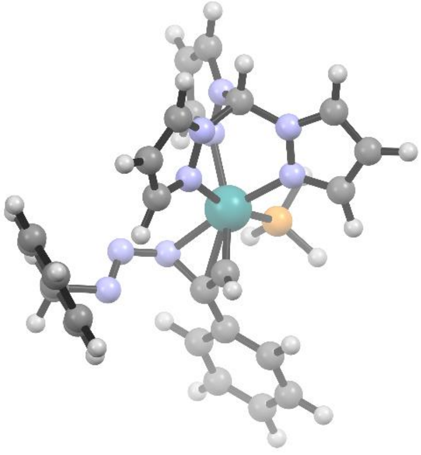

INT1

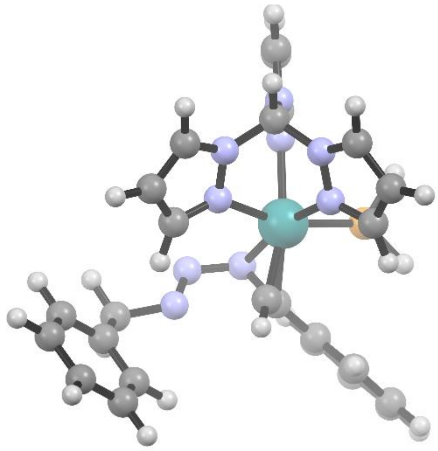

TS2

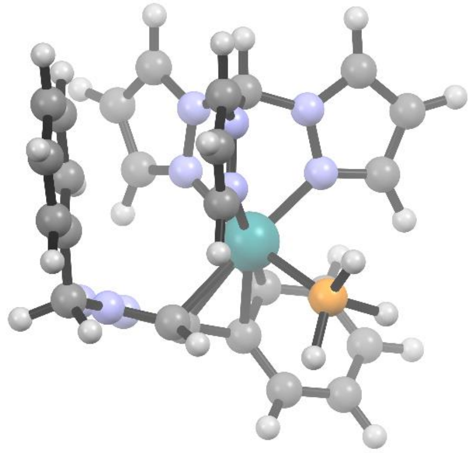

INT2

Figure S18. DFT-optimized geometries for the reaction starting from **3'g** (path b).

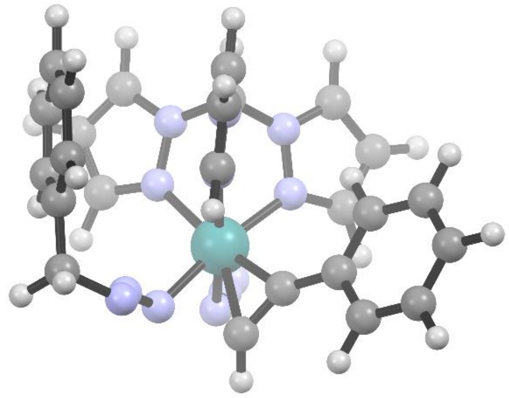

TS1

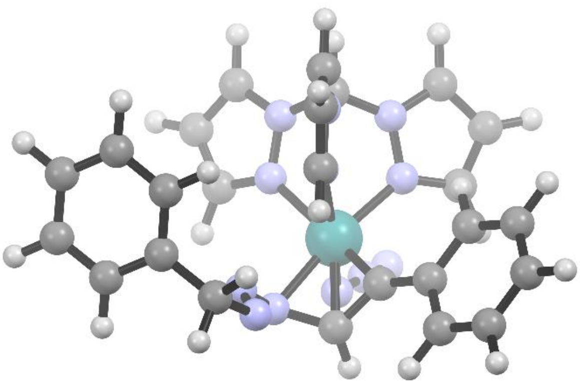

INT1

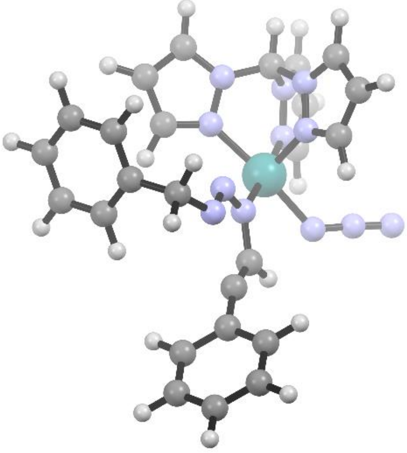

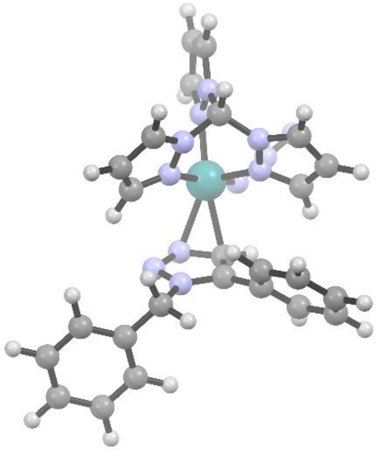

TS2 **INT2**

Figure S19. DFT-optimized geometries for the reaction starting from **3’h** (path a).

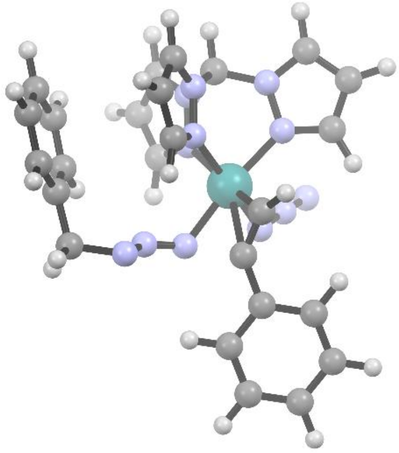

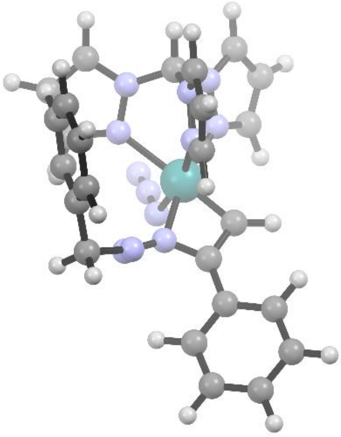

TS1 **INT1**

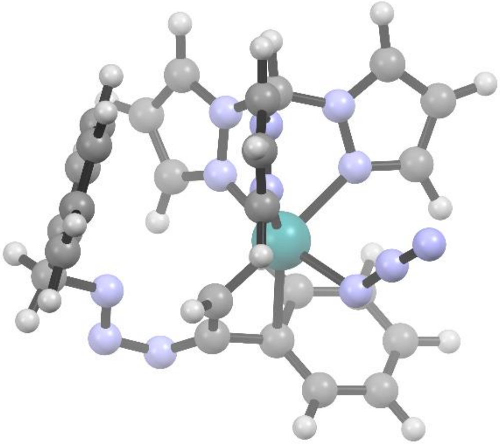

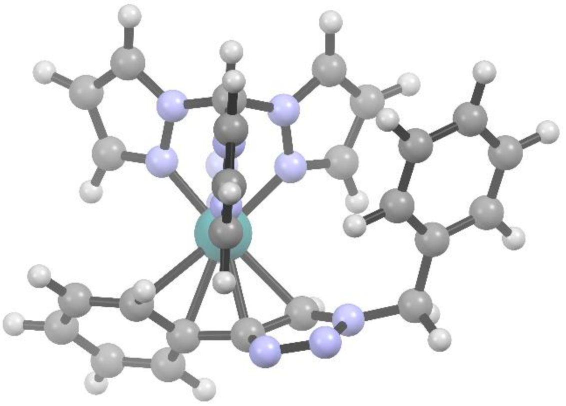

TS2 **INT2**

Figure S20. DFT-optimized geometries for the reaction starting from **3’h** (path b).

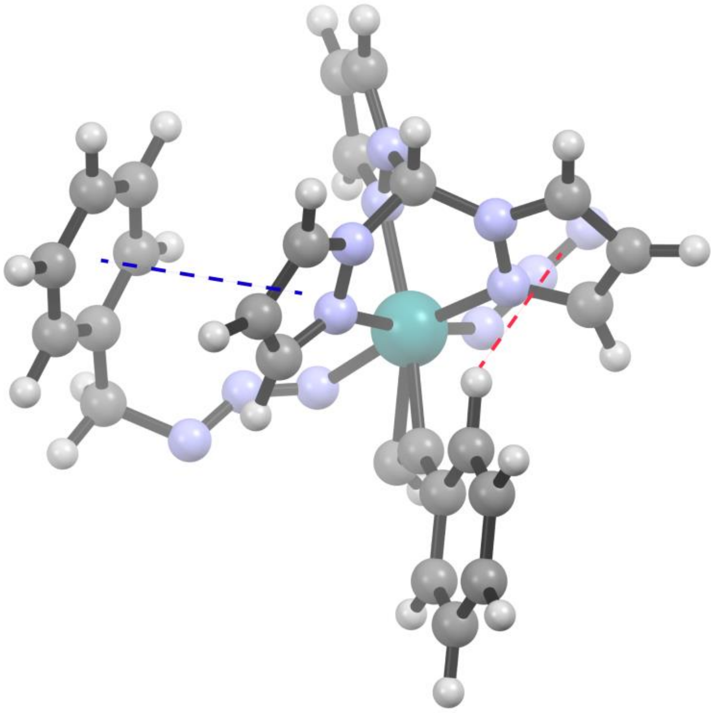

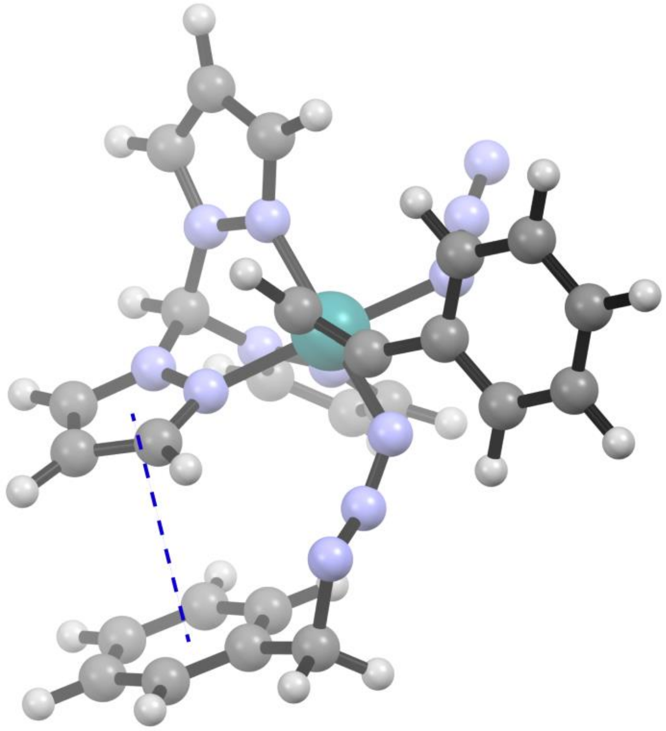

Figure S21. Comparison between the weak interactions present in **3’h_TS1_a** (top) and **3’h_TS1_b** (bottom). π/π interactions are in blue, CH/π interactions in red.

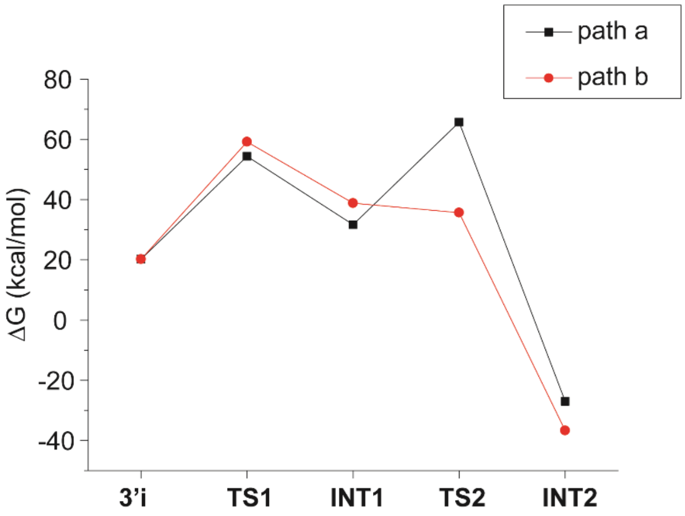

Figure S22. Energy path for the reaction starting from **3’i**.

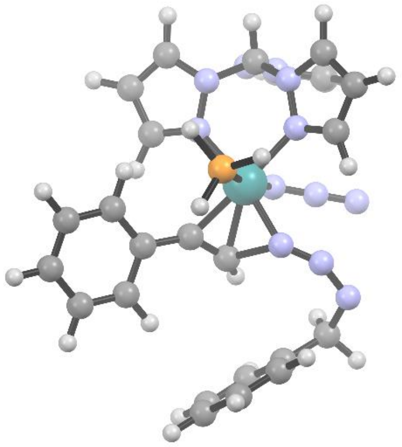

TS1

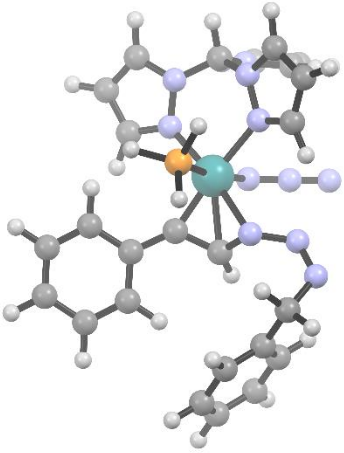

INT1

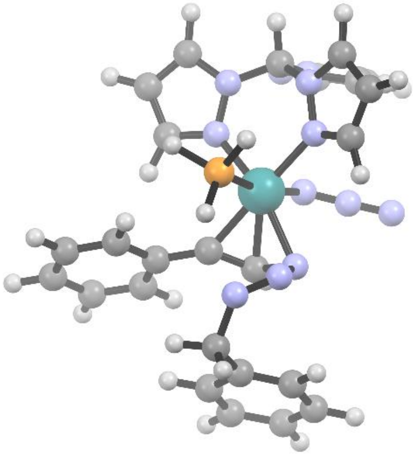

TS2

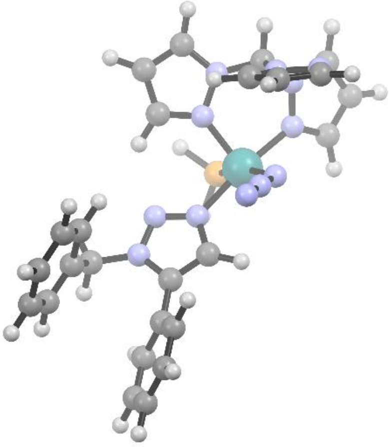

INT2

Figure S23. DFT-optimized geometries for the reaction starting from **3’i** (path a).

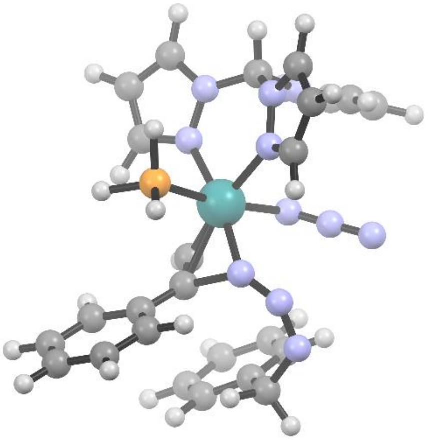

TS1

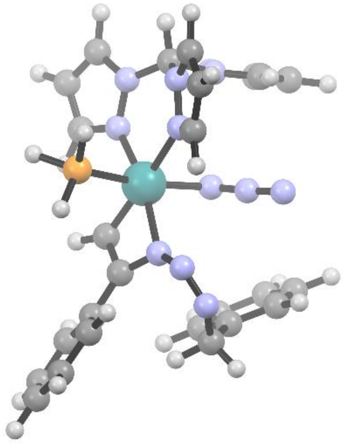

INT1

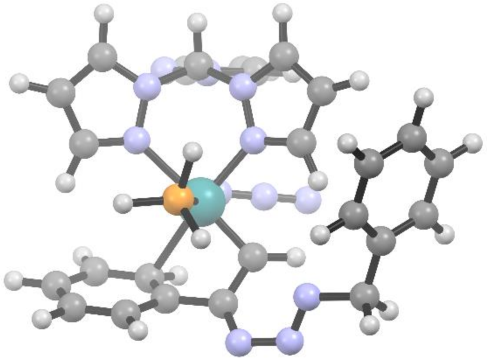

TS2

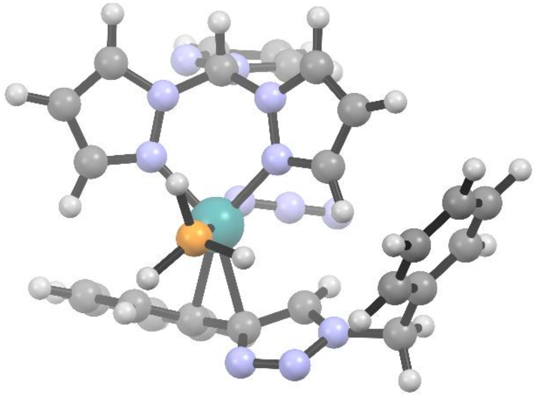

INT2

Figure S24. DFT-optimized geometries for the reaction starting from **3’i** (path b).

XYZ Coordinates

15
BnBr

H	-0.02454448395543	0.00368939856778	-0.00412325748584
C	-0.01438906457373	0.00680229764370	1.07692008419522
C	0.01174155554743	0.00399971186505	3.85311496902342
C	-0.02814442066508	-1.20613659784104	1.76537537042654
C	0.01191878956611	1.20687544573650	1.76964592917214
C	0.02657169429006	1.20793848163304	3.15922903022432
C	-0.01458732053454	-1.19590275940478	3.16006521509070
H	0.01810881164691	2.14124939855817	1.22724327597137
H	0.04666086276367	2.14346115712360	3.69936517513842
H	-0.02462801239692	-2.13382112628661	3.69765488420499
H	0.01777347183818	0.00149522738996	4.93348106148278
C	-0.06056702100877	-2.48194930484384	1.02901637834982
H	-0.49726463264773	-2.40399996237610	0.04279564414578

H	-0.49702681158212	-3.29671120981429	1.59031385517515
Br	1.81637658171198	-3.14299015795113	0.64690238488518

17
BnN3

H	1.81620295337930	1.74892567479641	-0.21810986169289
C	0.84115228988721	2.10885300418482	-0.51647381123519
C	-1.65927081986861	3.02413612417266	-1.29598060412602
C	-0.25801772093328	1.25779042960800	-0.43658426803417
C	0.69328341617848	3.40786705834196	-0.98353473904430
C	-0.55721557073404	3.86756635820083	-1.37340565097422
C	-1.50970225526947	1.72737152353595	-0.82840464311435
H	1.55385102499409	4.05826551614273	-1.04592624606539
H	-0.67297234807848	4.87725498343442	-1.74026129674868
H	-2.36654820059468	1.07050710475798	-0.76881045711768
H	-2.63331777632240	3.37670021828784	-1.60345838707326
C	-0.10367906847897	-0.12777880287040	0.10940980595302
H	0.90955117568843	-0.49612301923375	-0.04560907365500
H	-0.79871137200585	-0.81535636414729	-0.35829993381703
N	-0.42292629567883	-0.20798232574857	1.56637935032623
N	0.25289590271548	0.47111944915111	2.31392900163901
N	0.81507466512166	1.06584306738528	3.10435081477993

31
III

H	1.25356890149351	-0.67403213260158	0.38581975797531
C	0.84367900506799	-0.39358458273619	1.34602242209369
C	-0.19241865159871	0.32236666968432	3.81770220622917
C	0.02023599916589	-1.28218289676593	2.03131721084483
C	1.15097926394421	0.84155757468327	1.89489356242540
C	0.63411244941732	1.20266700579000	3.13371868670288
C	-0.49530676669381	-0.91602910662260	3.26804215952246
H	1.79393683334949	1.52327483877546	1.35691379260764
H	0.87706307325713	2.16391198041528	3.56308346748656
H	-1.12222704855480	-1.60958308172923	3.80987292720809
H	-0.59391367960083	0.59363144395574	4.78345766009735
C	-0.29506237811939	-2.63015735338609	1.44676832857504
H	-0.86231981835601	-2.54380149318632	0.52396407830840
H	-0.87627009635118	-3.22615311450279	2.14306483459005
N	0.91712670314844	-3.35506988575319	1.08716920265898
C	1.97989580601269	-3.65682833460920	1.87535426282761
C	2.89300304711817	-4.19212722371111	0.99572940093393
N	2.35901470799648	-4.19179584423705	-0.24772489032237
N	1.16321390605749	-3.67340663294131	-0.19410319098031
H	3.87362901927265	-4.58041942244665	1.19979153666116
C	2.03731063906238	-3.40837982938796	3.30977164204219
C	2.15079386089007	-2.87946964708481	6.04298785258521
C	3.06174996888844	-2.61965134606704	3.83214704263907
C	1.07919683823943	-3.94260275509963	4.17087811629815
C	1.13618316191043	-3.67677908773578	5.52938621539303
C	3.11512786819546	-2.35562899421530	5.19188493077905
H	3.79971053497946	-2.19844719409341	3.16561340584552
H	0.29345210360983	-4.56964406853358	3.77665156007218
H	0.38856408792120	-4.09419170103806	6.18802427471075

H	3.90707853437089	-1.73547825241685	5.58596182928923
H	2.19089212590561	-2.66896553240231	7.10183571389963

31
IV

H	0.94747312215412	-0.42603513584976	-0.02938545837761
C	0.77011251251104	-0.13608128974104	0.99712200452066
C	0.31238142822948	0.60718796467569	3.63141854482185
C	0.15178723163809	-1.02987449980004	1.86809715902524
C	1.15832111792844	1.11849745393787	1.44044796628964
C	0.92908559899995	1.49324042719397	2.75963010264707
C	-0.07224010516435	-0.65129270794160	3.18654823964875
H	1.63513488228517	1.80642732593474	0.75711434901429
H	1.22979681884054	2.47218197733210	3.10418415187236
H	-0.54933002380992	-1.34380517762452	3.86606506532543
H	0.13184839586607	0.89266140793581	4.65777305058062
C	-0.25572030644396	-2.39335477976795	1.38883745057853
H	-0.83688855097344	-2.33690986565352	0.47313715321561
H	-0.84700323334458	-2.90759311784597	2.14128777804142
N	0.89799886119492	-3.22636281503196	1.06844202781913
C	1.85937471435773	-3.66641078502432	1.89928281750073
C	2.72877649428233	-4.36862595263573	1.09089201140484
N	2.24210486146854	-4.30752795496315	-0.18254247152567
N	1.14030293793745	-3.61504528745266	-0.19249325827369
C	3.95053333426440	-5.07934964618137	1.43652691154315
C	6.29952699954928	-6.45021457580152	2.09800399608378
C	4.61309494538195	-5.85875474876841	0.48476567787233
C	4.48539866662038	-4.99782832837111	2.72517097891362
C	5.64723396768757	-5.67836458161723	3.05157898452655
C	5.77658600272345	-6.53558603719104	0.81381203989737
H	4.20847853924367	-5.93138730964488	-0.51302235068019
H	3.99400332581182	-4.39684400259637	3.47619828152017
H	6.04602600436690	-5.60330614900670	4.05306415162441
H	6.27554253274132	-7.13419681891494	0.06510501383750
H	7.20589434543684	-6.97967694350737	2.35360630859428
H	1.84336457821475	-3.45476804607694	2.95033132213781

6
MeCN

N	2.00717730206967	-0.00333481064742	0.00059981586333
C	3.16248519783996	-0.02613138524096	-0.00986934370141
C	4.60329229279407	-0.05327347637948	-0.02575870201374
H	4.96931354261521	-0.80095440950123	0.67401466621060
H	4.99642731193667	0.91980774621945	0.25958645836802
H	4.95930435274438	-0.29911366445037	-1.02357289472680

3
N3-

N	-0.00000000048964	0.00000000622504	0.02142602215565
N	0.00000000097929	-0.00000001245007	1.19999989180589
N	-0.00000000048965	0.00000000622503	2.37857408603846

4

PH3

P	-0.00114983883221	0.00128475642995	2.20704627998522
H	0.17263662322584	1.18478916995240	2.97357281828756
H	-1.11068732990165	-0.44613961106049	2.97311014466634
H	0.94354054550802	-0.74206431532186	2.96415075706087

14
PhCCH

H	-0.00994013309711	-0.01546150155855	-0.02013083429436
C	-0.00330340938106	-0.01227592467016	1.05946608906052
C	0.01350865707136	-0.01887405686324	3.83945405235667
C	-0.01193403762624	-1.23088448448345	1.75093141436500
C	0.01349798910819	1.18372415396954	1.75682960115595
C	0.02193616998949	1.18550296681335	3.14638139135488
C	-0.00330102986706	-1.22061330696423	3.15203069321190
H	0.02003995541538	2.11763897333340	1.21383194431718
H	0.03504905100717	2.12089736536891	3.68654267918786
H	-0.00994923468369	-2.15738800261958	3.68872999090498
H	0.02003869298200	-0.02234653157541	4.91971511225316
C	-0.02945124807502	-2.45742531977286	1.04292157349179
H	-0.05477347768521	-4.42342615844964	-0.09080557825992
C	-0.04341794515819	-3.50306817252809	0.43910187089438

43
3’

Ru	-0.00863266545745	-0.02763092297328	0.02146940927149
N	0.01339889862276	0.04484958594740	2.01324106868491
C	0.01480376880931	0.06756015742437	3.16551506501688
C	0.01428733444974	0.10115276879216	4.60060512778697
H	-0.87121236619139	-0.40252591265524	4.98268709819444
H	0.01216440583301	1.13255999091223	4.94700674685148
H	0.90005360241145	-0.40022797059986	4.98505597878567
N	0.28091594872663	-2.11592157922689	0.02712763735985
N	-0.48996826954060	-2.84723959265450	-0.80029143494329
N	-2.00755035805317	-0.49612348622994	0.01384672667146
N	-2.42301938942863	-1.47979376474181	-0.81343270637949
N	-0.08247058153539	-0.23316162830889	-2.02086733847142
N	-0.80286909578220	-1.25741156546756	-2.52640515963382
C	-1.45932403094005	-2.17326383217098	-1.62918874756038
H	-1.98145063830062	-2.91474569669831	-2.22112596460581
C	1.06615252462151	-2.97933246825126	0.66024764445870
H	1.77795094094388	-2.62432870709487	1.38200601213524
C	0.79397970697755	-4.28347252368399	0.23196879417551
H	1.26579826976706	-5.18917100234919	0.56185663282864
C	-0.20613612628036	-4.16777731965041	-0.70242885055292
H	-0.73211420430987	-4.89203090997373	-1.29573731915194
C	-3.08601320451985	-0.09913610531613	0.68302699283264
H	-3.00538631071779	0.68606567153504	1.41078781345236
C	-4.20356448164949	-0.83660153489174	0.28104976035841
H	-5.21005595484280	-0.73875814965804	0.63995088487796
C	-3.74954106920499	-1.71340025067237	-0.67237665396001
H	-4.24070304734784	-2.46924478923370	-1.25586013306665

C	0.46202147533810	0.38398856511829	-3.06545766684134
H	1.08766612710412	1.24299133438264	-2.91287030593155
C	0.08780384560730	-0.25283988951658	-4.25238239056721
H	0.36750561056028	0.01918093613019	-5.25199828189924
C	-0.71610154859211	-1.30024204376211	-3.87712802183627
H	-1.22801924499665	-2.05948314154195	-4.43797199345307
P	-0.40517779301706	2.22382435866892	-0.06902701166909
H	-0.12769789331398	2.89401448241871	-1.27822303763099
H	-1.72304745563672	2.67009894634073	0.15966696312743
H	0.29823822307560	3.04310612846809	0.83654673889757
N	1.95729957527855	0.29957612198877	-0.03816552887218
C	3.09875154409696	0.45069552182970	-0.08812113176680
C	4.52025758632054	0.63977400850749	-0.15255287222055
H	4.99178725740874	0.21463858467781	0.73109084769269
H	4.75270695171298	1.70151047452578	-0.20225431034922
H	4.91913213199296	0.14793714965500	-1.03731708209707

37
3’a

Ru	0.08199088219853	-0.03299175730725	-0.01673944511311
N	-2.02098235464135	-0.27088732440402	0.06100937808711
N	-2.69411566797997	0.14074882924989	0.95192991711248
N	-3.40130100239097	0.51630997532991	1.79209825396075
N	0.33217514236988	-2.12862223230830	-0.07274803304320
N	1.20908646367629	-2.61184729230319	-0.97385528458969
N	0.04915911061866	-0.16407819633090	-2.05628050485423
N	0.97423545903819	-0.95365666511475	-2.64942524541440
N	2.11827147522970	0.03451958266532	-0.15806558532044
N	2.71588386819395	-0.78654890068575	-1.05172842679639
C	1.90576017966234	-1.68443962420873	-1.83196569646730
H	2.55982791075304	-2.24811559648713	-2.48474788582149
C	-0.14338696288437	-3.18551939387957	0.57584697231766
H	-0.86896052737551	-3.04837852893222	1.35547249505473
C	0.43745457756396	-4.36143000838501	0.08312796875489
H	0.25120636214052	-5.36728330116905	0.40831281510922
C	1.30011318895865	-3.96267386588965	-0.90866268566747
H	1.95700485133487	-4.50583931751687	-1.56170920097674
C	-0.68853496096001	0.33282030752024	-3.04752475214864
H	-1.50189553162491	0.99652193490410	-2.82397060716960
C	-0.23365683907545	-0.14536156374712	-4.28250912275691
H	-0.63078630812801	0.08128174818426	-5.25355775019774
C	0.82694384807110	-0.96839055132168	-3.99864123680127
H	1.47958184963623	-1.55282768088160	-4.61936210468002
C	3.10759554550802	0.68502639179454	0.45205918721731
H	2.87957538396417	1.40634886675032	1.21375076505614
C	4.34665899401038	0.27426305164606	-0.05432602060640
H	5.31752836703410	0.62426097575717	0.24059985582862
C	4.06764751359602	-0.67035478594434	-1.00999828055223
H	4.69334170502471	-1.25878908116887	-1.65423527094171
P	-0.13785609200821	2.21033433089126	-0.03703776228580
H	0.93292248343463	3.01764834597301	0.41790209744010
H	-0.36841496073330	2.85852511961934	-1.27460017901882
H	-1.17997571295393	2.80701401302910	0.71116703607501
N	0.17007326462252	-0.12909679935786	2.09162528058177

N	0.18838661306871	0.79671100611554	2.83795269407334
N	0.20927188104684	1.66132798791382	3.61323636455448

42
3’b

Ru	-0.04455218965072	-0.05392446408992	-0.08759010036108
N	1.93835627736850	-0.04059801375545	-0.02772720181007
C	3.09138936712774	-0.04111647258304	0.01893932008689
C	4.52586128962379	-0.04331256673131	0.09457259390935
H	4.92272657542003	-0.94607307144218	-0.36523477192716
H	4.84187828227506	-0.00921202758792	1.13541287010679
H	4.92954116757764	0.82331590675031	-0.42503621594149
N	-0.09617911117501	-0.08703773279645	-2.13952928286527
N	-0.94761988290630	-0.95321988985821	-2.73064706218158
N	-0.09725199953620	-2.09830934146934	-0.20026581898304
N	-0.94460792001008	-2.65809805810897	-1.08955392848174
N	-2.08797404126052	-0.15309159073003	-0.18229579915012
N	-2.63661732729537	-1.00522243361666	-1.07393575669081
C	-1.76803222668364	-1.80069335980104	-1.90375120229536
H	-2.37867327407588	-2.42006708131828	-2.54749208188550
C	0.51914161065439	0.55432247289126	-3.12929335871393
H	1.25324795766698	1.30421750042148	-2.90181238649026
C	0.05552491786395	0.09292185747861	-4.36688696943542
H	0.36538791148219	0.42101888472708	-5.34068157331813
C	-0.88103222575212	-0.86986464774082	-4.08231424250877
H	-1.49506128271097	-1.49242274735522	-4.70541899455727
C	0.52488784128314	-3.10734238925069	0.40092145851601
H	1.25670498289463	-2.90375020504631	1.15955419875038
C	0.06949285159491	-4.32837501646376	-0.10987598724069
H	0.38514801177052	-5.31249092670068	0.17939912253279
C	-0.86974538283937	-4.01064523132702	-1.06009243226177
H	-1.48144934021708	-4.61168328441787	-1.70620770092951
C	-3.10510999110303	0.43739119473069	0.43681169894412
H	-2.91109692890250	1.16646168516909	1.20058375968581
C	-4.32023506309898	-0.04353351887005	-0.06465982770160
H	-5.30858910099571	0.24415090372745	0.23896878944394
C	-3.99017609004338	-0.96506966832864	-1.02793257480108
H	-4.58310851150827	-1.58531802213399	-1.67337980532974
N	-0.12655139367302	-0.16001549014042	2.01728883087689
N	0.54939793876170	0.54828652126894	2.69817508023965
N	1.17795531388673	1.20709325544462	3.41395692453317
N	-0.08146071230423	1.92779424501992	-0.01125309945958
C	-0.12392612608748	3.07984757789049	0.04066095421542
C	-0.17836000222531	4.51243679489940	0.13123248417534
H	0.67200232089422	4.95322822065037	-0.38507671006706
H	-0.15428502011527	4.81806474665371	1.17546857489626
H	-1.09572947397566	4.88090548394090	-0.32326177552467

39
3’c

Ru	0.06521910630900	-0.02630606206837	-0.04359055024591
N	0.14311565955886	-0.08217744472964	2.06954026906149
N	0.20242146985895	0.88636105366641	2.75791291869618

N	0.26464645425619	1.79321297440150	3.48017911661397
N	2.09598692697211	-0.13844952310422	-0.18977529997843
N	2.61617855958748	-1.02500337224231	-1.06823120251472
N	0.09206284291206	-2.07188559837339	-0.09390866594322
N	0.91297629191975	-2.66572483489312	-0.98672565098833
N	0.04842691824063	-0.12052439644556	-2.08135544577571
N	0.87870783232500	-1.00779009723862	-2.67470054913408
C	1.71746781572709	-1.83646489742829	-1.84768255582411
H	2.30342467818220	-2.47956743885915	-2.49126161748213
C	3.13785377154296	0.46691810310055	0.37649259965785
H	2.97244578621285	1.22344118244694	1.12024211201242
C	4.33522554902330	-0.03860955910595	-0.14578800999102
H	5.33396455591632	0.25368428776302	0.11741954985664
C	3.97282618403302	-0.99070622505981	-1.06621820801482
H	4.54327962348515	-1.63632620701228	-1.70690032833623
C	-0.51519738305217	-3.05994201219005	0.55689804322182
H	-1.22589787296893	-2.82922656931667	1.32748608317532
C	-0.07717681587630	-4.29957704076777	0.07445473037305
H	-0.38811607639050	-5.27260336705690	0.40403682846323
C	0.83565602693587	-4.01728457143488	-0.91200094533457
H	1.42577567437762	-4.64172265842714	-1.55590067127072
C	-0.57632059012987	0.51128028043951	-3.07264290145729
H	-1.29822962026690	1.27240308194995	-2.84447117900291
C	-0.14108255928922	0.02238672713016	-4.31092517849316
H	-0.46668593592298	0.33587385367797	-5.28453091377938
C	0.78872711911535	-0.94709436931757	-4.02734438859772
H	1.38170959071776	-1.58950578941002	-4.65058443075817
N	-2.04999404130389	-0.03495456932412	0.02900074310251
N	-2.64701890724803	0.70724909574241	0.74348600721574
N	-3.28050987436930	1.40725548590163	1.41807817296120
N	0.07929144977517	1.94150100981659	-0.02477105855253
C	0.09089145456706	3.09717886565741	0.00729003039691
C	0.10588616003059	4.53344251540955	0.05933464769340
H	0.55931877232988	4.86834157669300	0.99045704567123
H	-0.90971601831800	4.92109212817610	0.00552246940677
H	0.67888942122381	4.93464438183314	-0.77429161610448

40
3’d

Ru	0.00279836068357	-0.11367464730142	0.03114407861596
N	-0.03331269820205	-0.30441556778748	2.13332995827990
N	-0.04155666922197	0.60551176918220	2.90230644278836
N	-0.04332123257224	1.44429368142716	3.70267922430007
N	0.09418221597072	-2.21758770826805	-0.10443918737177
N	-0.72159448931400	-2.80793118432897	-0.99870379557580
N	-2.03187296874030	-0.38908236738063	-0.03355441321971
N	-2.52074266584164	-1.26930767339124	-0.93422978409588
N	-0.04940342553309	-0.16323366101294	-2.02218924928910
N	-0.85593766359829	-1.07547432670705	-2.60846598738029
C	-1.60770501775595	-1.98681693543278	-1.78649905342467
H	-2.18246338691518	-2.63428659838599	-2.43597812064443
C	0.78706011147765	-3.19803014059831	0.46283408582690
H	1.51255941040459	-2.96811186710491	1.22052539053318
C	0.40875516207489	-4.43405860939346	-0.07550209180455

H	0.79123091226297	-5.40278983266552	0.18377942303176
C	-0.55917385926695	-4.15290072000166	-1.00866224245185
H	-1.13664756689452	-4.77544995878876	-1.66600930170951
C	-3.08268274003300	0.05628645265222	0.64920717904271
H	-2.94327158411663	0.77688573828717	1.43274997963430
C	-4.25600890890480	-0.54503783427447	0.18131013182089
H	-5.25605935582246	-0.38111542927813	0.53442604988630
C	-3.86616370758811	-1.39107625896556	-0.82699220543024
H	-4.41278308119251	-2.05614178196606	-1.46871523125627
C	0.54204953236974	0.49179397501826	-3.01829428759748
H	1.23890871499528	1.27853025137255	-2.79852856270939
C	0.11261102306461	-0.00844733504409	-4.25259937022205
H	0.41387980351288	0.32168378181675	-5.22832505859598
C	-0.77794396319492	-1.01101511194361	-3.96067517574042
H	-1.35473385437309	-1.67287405176899	-4.57890654845350
P	-0.18530161527346	2.14913468414863	0.08885906575691
H	-0.37484050849603	2.82838892366645	-1.13561485976845
H	-1.24132693910167	2.72468389295851	0.83048895226403
H	0.89273121149512	2.90025836622072	0.60614429234073
N	1.97938518116436	0.03289528952218	0.05992860898623
C	3.13182000440076	0.09672892578350	0.06929000047117
C	4.56627839884926	0.17787382682637	0.07579388163853
H	4.96464889155294	-0.30837578758357	0.96405129464347
H	4.88048456747605	1.21963083617038	0.07391862578511
H	4.97185440019751	-0.31380500567936	-0.80620213890512

46
3’e

N	-1.63670087127039	-1.19377097823740	0.08052562964245
C	-2.59905059865188	-1.82867924544878	0.04906574362650
C	-3.80261128787176	-2.61069964408488	0.01290819509074
H	-3.55668007832583	-3.66824571763247	-0.05763099886000
H	-4.40517049401946	-2.32982192268191	-0.84841515697019
H	-4.37961760645608	-2.44196445286099	0.91985578970130
N	-0.03727046382383	-0.42057750440082	2.36832335276676
N	-1.09327122799836	-0.51811792306541	2.90710563457861
N	-2.09717153621086	-0.61413073024362	3.48001203974150
Ru	0.03432052752582	-0.12771451456592	0.22452326167855
N	1.07982235773190	-1.81913892362224	0.11881466048235
C	1.65352976902004	-2.81862744356938	0.07575661947372
C	2.38077982575841	-4.05576441772088	0.01679292656730
H	3.42777818158397	-3.85995387832911	-0.20590245861176
H	1.96794516493724	-4.69395795575675	-0.76170729294466
H	2.31363161822817	-4.57371474821742	0.97121499104186
N	1.75002501570494	2.37454200300987	3.44010089685579
N	0.62192004885748	2.56769248152127	2.73837619935460
N	1.77134241570602	0.98051667068883	0.39763250036356
N	1.79813630282952	2.30290018283350	0.68079575444405
N	-1.05995630450893	1.61963555396734	0.35115972154878
N	-0.53800004630373	2.82534595259652	0.68065713456223
C	0.72272598197835	2.97840699661306	1.34391890549054
H	0.96152913461719	4.03703689610493	1.29677270754388
C	1.33216541069787	2.18730495407013	4.68919402111160
H	2.05192531538059	1.98862715651830	5.46434001555270

C	-0.06299859415164	2.28347039490351	4.78990247769890
H	-0.67691035472229	2.16502853717425	5.66301572920349
C	-0.49139056606225	2.53964835534289	3.51112077765195
H	-1.47505890765576	2.67267638720463	3.10454243268373
C	3.00745245707502	0.67226729985817	0.01049186277734
H	3.22979208164300	-0.32530317766605	-0.31556935531198
C	3.84047802112824	1.79207252522808	0.06944081269140
H	4.88265298611917	1.84280270708138	-0.18063701876121
C	3.04320695352918	2.81448231298203	0.50867803140739
H	3.24427737723157	3.84615394370769	0.72732828408896
C	-2.29786658179198	1.87008382774396	-0.07125224254389
H	-2.91060706723143	1.06593908677887	-0.42946908812673
C	-2.58227670293394	3.23561790449235	0.00239906518773
H	-3.49699680124893	3.72610317244966	-0.26935329537074
C	-1.44310164758378	3.81948987102669	0.48723775256970
H	-1.19441140672082	4.83720171901138	0.72395787655840
P	0.13868956938046	0.12302668333407	-2.03609247398242
H	-0.83883423233787	0.93388973809005	-2.65407861314248
H	1.30839044618744	0.68625207294801	-2.59384068932925
H	0.02908641503015	-1.03513220917749	-2.83484311978313

43
3’f

N	-1.87354872784060	-0.72146132464536	0.02341082585712
C	-2.93854390067100	-1.16230757389445	0.10417819641363
C	-4.26324471830432	-1.70856450094213	0.20825017587826
H	-4.49884829948606	-1.91723641589703	1.25020445818937
H	-4.33450784127035	-2.63379887790231	-0.36025047067685
H	-4.99094135836706	-0.99921928255233	-0.18113721381854
N	-1.02607403201503	2.04382031512733	0.03256160234043
N	-2.07859847120538	2.20814468212224	-0.49206148837725
N	-3.10669992043021	2.41388335112157	-0.99401400880276
Ru	-0.07581430890628	0.08759050774455	-0.02971202218589
N	-0.03972684044522	0.14996134949668	2.09122460472205
N	-0.70210322572846	-0.55587610738226	2.78401614466189
N	-1.32142040417610	-1.21508508559454	3.51077255872063
N	1.54097702032694	3.95656459357309	-1.50267386597737
N	0.99658334339016	2.93867688025118	-2.18813917923230
N	1.80124574718902	0.94698602764094	-0.02429184217290
N	2.44793303847775	1.36338047847673	-1.13700693097487
N	-0.03683366702422	0.06064847844367	-2.08680986774849
N	0.91936259001349	0.65794194413247	-2.84240297199804
C	1.76055573950779	1.70541884745442	-2.34695255280665
H	2.53276834366643	1.85619592454535	-3.09621151369121
C	0.69023896674938	4.96462598570596	-1.67851749615281
H	0.88738020170173	5.91230537355773	-1.20801187023162
C	-0.38814021796876	4.59687494751632	-2.49521313354707
H	-1.22885013380806	5.19335378312445	-2.79626527075561
C	-0.15473587999256	3.28204287820708	-2.81474992197120
H	-0.71900810932294	2.58399279754480	-3.40191443438018
C	2.71152114834286	0.95732831334249	0.94605646421014
H	2.42875737770621	0.61987367884295	1.92503226602736
C	3.94515445285810	1.40595068619870	0.46399073781624
H	4.85902249011969	1.51941459626119	1.01499283409619

C	3.74384120080897	1.66610420949371	-0.86511298081806
H	4.38966349466330	2.04788116082551	-1.63304348932500
C	-0.73163858565242	-0.69937172527836	-2.93287041827281
H	-1.53193061372410	-1.30888603499504	-2.56027669436336
C	-0.23759559467549	-0.57908435162038	-4.23539099236700
H	-0.59922522320232	-1.07248031407066	-5.11706699333293
C	0.81165368143789	0.29488588106227	-4.14964659629737
H	1.48005297100677	0.69958997554555	-4.88619114335782
P	0.88861750645744	-1.95018528282345	0.02677719636740
H	0.86917422422651	-2.74982925274947	-1.14140426833749
H	2.26486598713306	-2.04224532278212	0.34291738876184
H	0.38223054843343	-2.89458619422912	0.94863417791095

62
3’g

Ru	-0.08913453371148	-0.88382543510097	0.27938094285438
N	-2.04673725192940	-1.50520691494316	-0.25925124689771
N	-2.86710158195865	-0.57521174306793	-0.78445679460021
N	-0.98399128473515	0.81557374369314	0.96741912201269
N	-1.96286627148406	1.37145645954064	0.22129047906715
N	-0.16380100357788	-0.03299027532692	-1.60846271443054
N	-1.25057588211772	0.69676140421085	-1.93474128577379
C	-2.35875831713285	0.75375847352656	-1.01694360061060
H	-3.14370407941073	1.35180231529742	-1.46166664834868
C	-2.72680067800891	-2.64613283018738	-0.26739417379566
H	-2.27480084098767	-3.54185311097244	0.11627716327148
C	-3.99908361337614	-2.44497556525412	-0.81069322947722
H	-4.77282971108917	-3.17648752171779	-0.94356256417639
C	-4.05729035179690	-1.11275620579843	-1.13892549956360
H	-4.82474752706963	-0.50777785209279	-1.58406575732134
C	-0.88395337894303	1.56202731988656	2.06414260628570
H	-0.16254418117105	1.31226265742585	2.81857858716998
C	-1.80937893385492	2.60721104046624	2.01970795372515
H	-1.96047354163182	3.37169133123025	2.75726404851425
C	-2.48717633600877	2.45757146453321	0.83549483926952
H	-3.28323629976003	3.01569883603051	0.37948725912749
C	0.62978958465150	-0.01240602501098	-2.67522901040578
H	1.57550340147050	-0.51864184492205	-2.65522057857342
C	0.03981588378646	0.73071820431487	-3.70026230732680
H	0.44477810840140	0.92393167895659	-4.67483957948613
C	-1.16475991903650	1.16217576993799	-3.20351226537922
H	-1.95735241475247	1.74921435858078	-3.62814174781080
P	1.98344703303311	0.10615400683320	0.53166684254828
H	3.12333008082645	-0.69451758045956	0.70495542342678
H	2.36865858791986	0.90573199613402	-0.55997017347297
H	2.12375397153784	1.02009197254073	1.59194178792470
H	3.99155408792768	-2.56412945206882	-1.04775262698249
C	3.86348160221429	-2.14835971600059	-2.03745965767723
C	3.54472406073101	-1.08572154981303	-4.58108723834130
C	3.19165395952201	-2.88363555627127	-3.01159940551284
C	4.37000377162240	-0.89035927859616	-2.33160074990982
C	4.21146071789764	-0.35713867952529	-3.60381661785483

C	3.03458032935905	-2.34101813999942	-4.28478631682130
H	4.88896575204839	-0.32894364378704	-1.56881806038053
H	4.60549376206600	0.62208070115759	-3.83316857974364
H	2.51060891452909	-2.90581030104967	-5.04297947235193
H	3.41930727513189	-0.67454131405816	-5.57203375924624
C	2.59851550733491	-4.21316071761314	-2.67451545825331
H	3.18389089588812	-4.72676501338998	-1.91344591375554
H	2.51936603445776	-4.85231495808134	-3.54554273600206
N	1.19143628764584	-4.10824202880240	-2.16039887047658
N	0.98344312507949	-3.28804666219608	-1.29753727109513
N	0.67998882499326	-2.59025352123680	-0.44606013510652
H	2.34463078953926	-3.55112499039554	1.78527111862978
C	2.69141207679489	-2.83257785415267	2.51146602425514
C	3.56661246091161	-0.94894219138218	4.36364575652813
C	1.82961121714269	-1.81365009042008	2.93619470312512
C	3.97704383566908	-2.90134472118391	3.01563026573923
C	4.41799932155162	-1.96090963130480	3.93980831426961
C	2.27734013706357	-0.86803338683210	3.86546811881288
H	4.63834825374085	-3.68909837080807	2.68654007037603
H	5.42429217227240	-2.01693690643315	4.32796016135778
H	1.61170316367238	-0.08127464446471	4.18586245870842
H	3.90732007318267	-0.21913427964898	5.08294957233115
C	0.51754489274231	-1.72590957947029	2.40502320662090
H	-1.70683154712238	-2.04020678725578	2.36779386399636
C	-0.68948047369227	-1.79754686320062	2.15672135701407

61
3’h

Ru	-0.04404615214819	-0.03431813011682	-0.12960894145424
N	-0.00066437904364	-0.09863705028056	1.97538514973870
N	-0.01995368017534	0.90917265543904	2.61276812367299
N	-0.02921570357584	1.83931013227278	3.30100783554706
N	0.08607359741897	0.37105429983639	-2.16747967119925
N	0.01831926080734	1.66560711529012	-2.54418731799494
N	-1.46492765807287	1.40993494892243	-0.04754852765727
N	-1.27326479374552	2.54133762434107	-0.75942657267714
N	1.32669200766695	1.50037711109351	0.01111129510355
N	1.08876756379943	2.63333111408006	-0.67871281255377
C	-0.06866306764792	2.69125948324230	-1.53499139896566
H	-0.09177078834435	3.65666391651123	-2.02427652234539
C	0.26695622682726	-0.32843145606727	-3.28315189982589
H	0.34588669499040	-1.39829939431908	-3.23502904872246
C	0.32342938611330	0.52830422151702	-4.38693276665566
H	0.45843145501343	0.25503881402081	-5.41584790426715
C	0.16861788546842	1.79607237134138	-3.88393589712369
H	0.15081677344091	2.76396164554083	-4.34869524364307
C	-2.62169816541366	1.56150848473427	0.58954452272051
H	-2.98505673789197	0.77813759554758	1.22678699070560
C	-3.17686964188383	2.80775904438679	0.28398608994565
H	-4.10213410516340	3.21266106218641	0.64651272103032
C	-2.29484212209806	3.41153022144839	-0.57787841816397
H	-2.30125869227178	4.36466543388085	-1.07229317556803
C	2.45553087151254	1.70211034239835	0.68271272929476
H	2.84325671039586	0.93194287347700	1.32070515041508

C	2.94894533522441	2.98246526260955	0.41556218024501
H	3.84021235994936	3.42918369492141	0.81251555441090
C	2.05849210153185	3.55330859809720	-0.46032534405778
H	2.02769167096394	4.51221661920555	-0.94245746263035
H	2.54091696929292	-2.10702209638409	2.93823784327253
C	3.30936528552403	-1.34823197675899	2.89573442039028
C	5.27901881649698	0.60302419154123	2.78194595889342
C	4.32352913785569	-1.44949175295374	1.94659149421621
C	3.27770238742336	-0.27900395014191	3.78048334214677
C	4.26222402394780	0.69802829167046	3.72489114811319
C	5.30687477882400	-0.46341090562673	1.89604552900049
H	2.48254190439268	-0.20746053472203	4.50773276046135
H	4.23568750969361	1.53238115825749	4.41051699806368
H	6.09403732082118	-0.53462902462950	1.15840138351110
H	6.04654550216336	1.36184003291509	2.73526094206929
C	4.31620420661023	-2.56552391934152	0.95063762605024
H	3.82360295722909	-3.44923951664636	1.35360082221459
H	5.31955968203817	-2.83376402894778	0.64158224822483
N	3.62169880220569	-2.18745289380673	-0.32613138083829
N	2.48963789317242	-1.76387012539757	-0.20467228471031
N	1.40979698902888	-1.39498971850825	-0.22371903516353
H	0.28215581597757	-3.77498150818771	0.88563990320231
C	-0.43945961194360	-3.58631377893938	1.66504888636288
C	-2.29863336247768	-3.07304332366096	3.66802869411915
C	-1.41858574429111	-2.60505135188257	1.47331608771579
C	-0.39721764928013	-4.29997986711846	2.84982695325048
C	-1.32402402805726	-4.04589012001913	3.85403003381407
C	-2.34674707688210	-2.35052518149031	2.48824148190567
H	0.36165683356869	-5.05566554254169	2.99130981022493
H	-1.28635706912467	-4.60359953124507	4.77839746301670
H	-3.09528312233632	-1.58686534337648	2.34240087281139
H	-3.02004960622885	-2.87314632544025	4.44682338613499
C	-1.45065795312078	-1.85690516923733	0.27226720880804
H	-2.06037744590065	-1.26541282063485	-1.81019289043244
C	-1.61011836027126	-1.36103202230349	-0.84709712417432

65
3’i

N	-2.74406534927866	2.44780285228143	-0.02022458922636
N	-3.18397626110836	2.70127169493820	-1.09846774758108
N	-3.63869868716943	2.99387934011972	-2.12162819210018
Ru	-1.20365954774916	0.91853245879763	0.06869326675590
N	-1.05630783720631	5.10268859443166	1.47333858888331
N	-0.61437941967978	4.64243692682762	0.29178833572390
N	-0.24743601753530	1.92032273306912	1.60617809328208
N	0.62461624899206	2.93858000138188	1.40453559923732
N	-0.17392710333057	2.09420342682555	-1.31973174297683
N	0.66356456714965	3.10281555143008	-0.98769101655146
C	0.57520587217588	3.80177195535448	0.26160968471049
H	1.46340189312996	4.42405587177210	0.32321128785212
C	-2.02933066888848	5.95066729358757	1.15289227807243
H	-2.57215111043921	6.45317097142992	1.93458657221000
C	-2.19594658479765	6.04931357977061	-0.23603978028341
H	-2.90636588596957	6.64227541173644	-0.78090661940012
C	-1.25805563795518	5.19810580154966	-0.76456503319914

H	-1.02843848740992	4.93875836521790	-1.77999779227423
C	-0.09262151991396	1.56342900578084	2.87939542281556
H	-0.65300667241994	0.73788541874607	3.27100435058291
C	0.85840878850749	2.36670016162510	3.50945397323650
H	1.18524469724871	2.30992977903287	4.52972329595322
C	1.28859050776359	3.24020340668052	2.54806180574862
H	1.98836826872345	4.05424092305250	2.57334654509445
C	0.03724707334283	1.85836420311155	-2.61245432255804
H	-0.49123054633769	1.06235775969574	-3.10110843495690
C	0.99987493795934	2.73053170702776	-3.12290118819654
H	1.37114264923239	2.77543329630237	-4.12843885426132
C	1.37576814539935	3.51653715212147	-2.06554911443564
H	2.06406321227684	4.33713176508171	-1.98330008489738
P	0.63251620241743	-0.47994989795158	-0.01771949330308
H	1.67692890314779	-0.03404128531624	-0.85234882425194
H	1.33545339678752	-0.71797794074713	1.17804270211173
H	0.45655222827200	-1.79218672974595	-0.49311666581142
H	-4.18074231878206	-4.48938111278163	-1.16869737398201
C	-4.44197041177520	-4.68255095563701	-2.19933854669781
C	-5.12545281718362	-5.17690707915761	-4.84227322539793
C	-4.62002007633455	-3.61430729182849	-3.07490098465743
C	-4.60335707244233	-5.98744878139350	-2.64010026815790
C	-4.94602354077205	-6.23680841900946	-3.96363385866925
C	-4.96186924560876	-3.87145111957711	-4.39845989077412
H	-4.46676873329300	-6.80887905680127	-1.95154124413089
H	-5.07501721199644	-7.25311076071849	-4.30715112844858
H	-5.10329207878644	-3.04553045700168	-5.08172304934236
H	-5.39555324313685	-5.36562292718235	-5.87126640396261
C	-4.42183273451096	-2.21147632144717	-2.60110032187495
H	-4.80827426834655	-2.07947679045209	-1.59197962117263
H	-4.91641224090280	-1.50219290645173	-3.26282817452415
N	-2.95874866184351	-1.89468596618858	-2.60175491715030
N	-2.60421865075879	-0.94747324605375	-1.93106971750344
N	-2.11605513220882	-0.06040103597409	-1.40246669485881
H	-0.07505258882802	-1.66122109435160	3.20378731667176
C	-0.45445975472240	-2.43900270963745	2.55991253071329
C	-1.43797158819793	-4.44369064799418	0.90250117738363
C	-1.43366147823775	-2.13590795664409	1.60714603499724
C	0.02253311295632	-3.73365059868997	2.67640980584437
C	-0.46248785880066	-4.73708414175158	1.84776329735579
C	-1.92111338685704	-3.15368261902456	0.77881066492326
H	0.77555888210271	-3.95881626605802	3.41715511379366
H	-0.08383201215432	-5.74437767670652	1.93979181876327
H	-2.68025214373375	-2.92422273958007	0.04963340045125
H	-1.82465120572633	-5.22096325426697	0.25994448208472
C	-1.93158672905490	-0.81064755869702	1.45741716065828
H	-3.45347672385847	0.80997467882065	1.82945078810877
C	-2.63536959735311	0.20743067251542	1.50261529098145

PATH 3’i_a

65

TS1

C	-0.39067099217396	-1.04850394740753	0.81274519201038

N	-0.03668992420692	-0.34966131272520	-0.81717135495813
N	-0.43023773690479	1.67399741665967	1.33093152675866
N	-1.07646222937040	2.29612772006275	0.53999084275880
N	-1.73602283588333	2.92705474212291	-0.16656091718595
Ru	1.15563899917566	0.44427719723859	0.62838106503504
N	1.36549174667214	3.99267397297646	3.38838893200899
N	1.68693722611209	3.93885188121471	2.08598351720649
N	2.13827703827047	0.98583289223941	2.38245895916155
N	3.03123621192207	2.00860241465550	2.46200287033986
N	1.97647895328334	2.13156526510486	-0.30698393845675
N	2.85981156000731	2.98086893188092	0.26698910484872
C	2.88511057615279	3.20733789203150	1.68283610576608
H	3.76793745070754	3.80804085350448	1.88236808368303
C	0.32536209810151	4.81947386728872	3.43878544654367
H	-0.14229117654672	5.02496972956454	4.38597483994255
C	-0.00861122761673	5.31099587856684	2.16897781136973
H	-0.80374077773610	5.98163927325084	1.90221849253119
C	0.89603857482358	4.72888171711030	1.31672358344086
H	1.01537356278896	4.80635489307287	0.25370385364009
C	2.24000597024558	0.32909413572500	3.53206917681290
H	1.64244559744146	-0.54460502323037	3.70180538646057
C	3.19278595172688	0.92079326753461	4.36610613270731
H	3.48888597452745	0.60072539407915	5.34638110430206
C	3.66211102487649	1.99716449303041	3.66781447164876
H	4.36629751919884	2.76796142243227	3.91884639210286
C	2.01705408172428	2.39081583093960	-1.61252928300568
H	1.43029413740537	1.79830996903472	-2.28958534359123
C	2.90705481842807	3.43082792225036	-1.88074753115688
H	3.14405649704301	3.85641695203527	-2.83658578386325
C	3.42479002824826	3.79005365599352	-0.66313909799931
H	4.12731956151172	4.54909909024820	-0.37271600254126
P	3.02081761533581	-0.61272905926456	-0.27226780068736
H	3.41057734551022	-0.10893041174836	-1.52859062138775
H	4.24044492011686	-0.51735374454855	0.42792823401316
H	2.97942245980956	-1.99006395437349	-0.54636794131345
H	-3.80913403420039	-2.05349273385581	0.73595183127271
C	-3.25593150089438	-2.69164016441731	0.06056778905883
C	-1.81018245075541	-4.32171008984038	-1.64905525914042
C	-2.72923722934602	-2.15599932554765	-1.11242421975871
C	-3.06894938384117	-4.03007309702938	0.37310302158401
C	-2.34225143610257	-4.84967123646522	-0.48040803546237
C	-2.00867355829485	-2.98570059092225	-1.96694153417388
H	-3.48232626849616	-4.42971852489653	1.28796431096012
H	-2.18423541673505	-5.88908724833718	-0.23184877147457
H	-1.59098406820052	-2.58386030131490	-2.87857826017763
H	-1.24028161072443	-4.95137756391862	-2.31719147710068
C	-2.98614183945642	-0.71185912842235	-1.44491303082392
H	-3.16508410322102	-0.13866809743890	-0.53468585683056
H	-3.86872429766050	-0.61506804467067	-2.07197632442626
N	-1.89504153958579	-0.06638521878664	-2.22805729686294
N	-0.79747051863813	0.00975457933434	-1.68158253302696
H	3.23762222576006	-1.79466680962428	2.33653229802259
C	2.63424438371235	-2.68712642407184	2.34961147140092
C	1.09264522587588	-5.00379143498647	2.38747403907456
C	1.32472692197117	-2.65713230143136	1.84815693593925

C	3.15623257381941	-3.85549399884738	2.87003624868787
C	2.38952584041083	-5.01495206286325	2.89279102266805
C	0.56288049522181	-3.84180605548731	1.86620744290670
H	4.16480076396648	-3.86559818019801	3.25527085773017
H	2.80337419546185	-5.92676756445583	3.29757556551806
H	-0.43599059946354	-3.82705272882015	1.45824753121393
H	0.49944913375077	-5.90627981146184	2.39602334267477
C	0.72644893885043	-1.49820402453419	1.29685415378239
H	-1.39624144391379	-0.84290303523847	1.12211922781670

65
INT1

C	-0.32824480931935	-1.02952907553127	0.38889619725033
N	-0.10521799481876	-0.34017715468837	-0.73365602892052
N	-0.33173666802504	1.53319723601955	1.83145820802348
N	-1.10952866239306	2.25731663804845	1.28013172853635
N	-1.88299959268664	2.98410241462586	0.83321308425493
Ru	1.18300781219401	0.50429051857167	0.82903095780793
N	1.78922170604464	3.93528267828095	3.67704851100387
N	1.86423427005730	3.97461065392678	2.33655759863939
N	2.42913267381103	1.04503325152527	2.39785756936036
N	3.30668814166860	2.08325884383121	2.34239586887352
N	1.70595386078879	2.35024105830305	-0.15689359785220
N	2.69460433518277	3.16473486998670	0.27503973380551
C	2.98420306619979	3.31543765486296	1.67103268460313
H	3.87402281138536	3.93501191670508	1.73790485889269
C	0.76450969609008	4.72807485239924	3.97475093812115
H	0.47664257618222	4.85731469437937	5.00360537025418
C	0.19395373695446	5.29260386230648	2.82506436592299
H	-0.64718836800717	5.95658325270651	2.75659330943351
C	0.93302140325488	4.79256692076843	1.78240268748846
H	0.85467258023477	4.94470415763564	0.72364234513103
C	2.76085489026419	0.34545198948144	3.47550698925991
H	2.21636120182791	-0.54532785629241	3.72021917505816
C	3.84981393382769	0.92492386817307	4.13253497065410
H	4.33474827925926	0.57480060391611	5.02320031183810
C	4.15810951739325	2.03831539860084	3.40314591259628
H	4.88525288424220	2.81437608032680	3.55045646336203
C	1.51887983239250	2.65571885324204	-1.43822402273726
H	0.80070190920014	2.10306404814862	-2.01479673612562
C	2.36858090958518	3.69248307867235	-1.82860779766153
H	2.43856981730081	4.15148445001020	-2.79589180574550
C	3.09975584805751	4.00366508162694	-0.71074570679487
H	3.85052788921055	4.74867016302164	-0.52284430175074
P	2.99413333952697	-0.34712389015447	-0.35327197846406
H	3.67269979820255	0.58041980437315	-1.16537826100784
H	4.06806281497202	-0.90691567496865	0.36326000362858
H	2.71947513270526	-1.37141269438881	-1.27684662499456
H	-3.78285707851816	-1.20483140457871	-0.67737340636794
C	-3.40175847356991	-2.21516565792836	-0.66290893293841
C	-2.43398025579981	-4.81366958488911	-0.60352664689645
C	-2.33232502508142	-2.56647059938520	-1.48104368153053
C	-3.97319962585047	-3.15227152958000	0.18736664812754
C	-3.49263047357630	-4.45388349202352	0.22016700006700
C	-1.85650326847036	-3.87509615817912	-1.44547702514943

H	-4.79629071958690	-2.86227911952048	0.82451548561574
H	-3.93616664439022	-5.18126490850640	0.88434918843339
H	-1.02008757051085	-4.15530938611011	-2.07046170901786
H	-2.04573139840438	-5.82173208671290	-0.57969443550814
C	-1.69489552119453	-1.59066956023716	-2.43629716651410
H	-2.13197023934913	-1.72016034659073	-3.42464727992648
H	-0.62385036193559	-1.79202898950604	-2.49641316044906
N	-1.90138723850413	-0.17012299025048	-2.10439049387237
N	-1.15402776582214	0.38489547556247	-1.28857502460987
H	3.19611333074219	-1.96462492136523	2.34060472916538
C	2.52122572115984	-2.79692537625780	2.23818298566016
C	0.80254634611312	-4.96859513212536	1.94024089044534
C	1.27453315430031	-2.61529604337713	1.61268627715565
C	2.89716061143457	-4.04159818702505	2.69859474591668
C	2.03727738159148	-5.12768829099892	2.56098479192990
C	0.42935536606548	-3.73289253905386	1.45429874603034
H	3.86104202995511	-4.17171499980614	3.16724645253643
H	2.33405299344219	-6.09856601625715	2.92971303521638
H	-0.51868393068649	-3.61240068324130	0.95443481870622
H	0.14017566335438	-5.81348669475336	1.82354453804830
C	0.84246335505655	-1.36081571187183	1.11897600689593
H	-1.32668093473179	-1.11195761388233	0.81109964108402

65
TS2

N	1.49810649241674	-1.40912778741701	2.28713788390333
C	0.26809205594116	-0.24594042826607	3.98198550371723
C	-0.47051240483892	0.13356798691790	2.89716156621536
N	0.29925783485658	0.42483247457849	1.74414259359227
N	-0.94688920818784	2.75884447152403	3.09061194873235
N	-1.15990080439985	3.11151097660451	1.96719498101944
N	-1.41052917044853	3.49060286664931	0.90781567408291
Ru	0.84328872582645	1.67332011826247	3.45500631822403
N	-0.75717699065504	5.37319069415656	5.34253909844415
N	0.17232859044709	5.31196835797356	4.37573709422524
N	0.82718720601887	2.50981638873185	5.35490119813146
N	1.40063230946764	3.71018551378976	5.64086595380902
N	1.84611110030893	3.39654084013865	2.73878803180782
N	2.20204567735302	4.45163641459631	3.50070353474999
C	1.49406903023056	4.79199273791771	4.69980360253884
H	2.08193666040056	5.56168294729512	5.19182406015276
C	-1.77811673860110	6.01095733936238	4.77788301032814
H	-2.67661197383405	6.17934251262047	5.34570392056271
C	-1.49451696590035	6.37550015447773	3.45380900620503
H	-2.12826361218527	6.88704814776207	2.75387731335648
C	-0.22077838569830	5.91723546507462	3.22714874134390
H	0.40308812891717	5.95963043738924	2.35545953636401
C	0.58177197847508	1.93861419336062	6.52987387919727
H	0.16873457411985	0.95057682597411	6.56848850058058
C	0.96800059578331	2.77213235236408	7.57995377937574
H	0.88759955384769	2.57281130140435	8.63103139015878
C	1.47211651322188	3.89472971284234	6.98391450557127
H	1.85736481869833	4.81268868209495	7.38643875822252
C	2.53385979909554	3.51365065559962	1.60546466109078
H	2.44745240152346	2.75261183015821	0.85201418985788

C	3.31947351236120	4.66702952743800	1.62635618043374
H	3.97422699680235	5.02494565572783	0.85549979908276
C	3.08185302476048	5.24822186539995	2.84589547132210
H	3.44257123786220	6.15321807354700	3.29822390568949
P	2.97858672391536	0.89331961641684	3.96333257957592
H	4.03547960560753	1.67410897120004	3.45682542013913
H	3.31750165466156	0.86241237258928	5.32996767515192
H	3.34515148711494	-0.39798051397245	3.54667463614994
H	-0.27803810440259	-1.72034700329692	-0.14314232220797
C	-1.06863143222621	-2.01813841775749	0.53029080686686
C	-3.10861488394926	-2.80625086855626	2.23723954430472
C	-0.75321786724996	-2.49995894982356	1.80119523231992
C	-2.38920068195390	-1.91295439085160	0.12553816386946
C	-3.41366260933162	-2.29851343537896	0.98189050673652
C	-1.78647528998220	-2.90690658445145	2.64251754851215
H	-2.62015038581757	-1.53257752385931	-0.85885740362651
H	-4.44355141382317	-2.21163217031041	0.66729162926833
H	-1.55027056428642	-3.31158535078287	3.61447944851081
H	-3.89898338211942	-3.12532755738971	2.90113032889986
C	0.66854532444545	-2.64374605263528	2.23682589022296
H	1.20622174379457	-3.29967577302207	1.54934164330057
H	0.73637565880259	-3.08754406799934	3.22477644627406
N	1.34934476750830	-0.36433267247560	1.59168693441914
H	2.13580943545234	-1.20250783464851	5.68387042318221
C	1.12535427218982	-1.46517212055427	5.94949955653991
C	-1.47216938631005	-2.16301378479308	6.64037840620445
C	0.06087307042625	-1.04493477269928	5.14241313084293
C	0.89191708540461	-2.23540947643508	7.07348545120128
C	-0.40628463903849	-2.58229091466259	7.42715539024854
C	-1.24619463477363	-1.41500012141953	5.50018185277403
H	1.72452340443869	-2.56387665552701	7.67761394738415
H	-0.58665564706045	-3.17466391735648	8.31196888081871
H	-2.07575303791933	-1.08653448015665	4.89516981394394
H	-2.48429408351555	-2.42330230066888	6.91360228409748
H	-1.53160875398917	0.32444744522688	2.84588056198275

65
INT2

N	0.68084117919229	-1.22634314557529	3.66637312743009
C	-0.29182353010843	-1.22898288307070	4.61417942067556
C	-0.19858751610456	0.01248072184847	5.18908404627970
N	0.79761957836077	0.68580191638476	4.56220003904039
N	-0.47362217176376	3.13697382686466	5.47365509415082
N	-1.46399295849475	2.77894755812128	4.91212697518881
N	-2.45345977212629	2.46758669339316	4.40376077312941
Ru	1.49455136916723	2.54007399721728	5.12651681016757
N	-0.13508779153074	6.56582769189957	4.84810392023178
N	0.38658793466840	5.74422405228752	3.92416107608277
N	2.15941588581447	4.31614947807087	5.91583700479910
N	2.37945957441204	5.43005047230069	5.19032145322993
N	1.95086984176278	3.18351155525157	3.30814527506729
N	2.18310427458874	4.49324018299734	3.00897616104100
C	1.83271896021739	5.57761557227066	3.87605869232937
H	2.28816289258188	6.46763400321084	3.45184552629113
C	-1.42871787093419	6.62463955612010	4.54333752598729

H	-2.09015168606260	7.21638188379132	5.15204413557097
C	-1.73037853289187	5.85574712654838	3.41051936070405
H	-2.68587199463405	5.70455707902463	2.94468576288310
C	-0.53266574527074	5.30360897597330	3.02962623680878
H	-0.28093646768019	4.63883667734444	2.22624754893483
C	2.61585189654101	4.58139107636089	7.13740818178495
H	2.58622245670226	3.81873872911689	7.89370983127984
C	3.11534685609171	5.88652529628280	7.20142406977396
H	3.54724297035437	6.37739247344223	8.05220857416251
C	2.94399243563482	6.40592267803665	5.94513400042220
H	3.15848294721714	7.36961186256783	5.52343047697119
C	2.22313363647475	2.50354163602111	2.19275401128626
H	2.14881064473125	1.43319443323051	2.20193760788914
C	2.59894647523788	3.37325223115746	1.16737479266030
H	2.86823670047682	3.10932540077217	0.16286158279833
C	2.56126059982930	4.62768852481398	1.71027672026117
H	2.75123503634176	5.60169143896597	1.30006358275246
P	3.59704747068066	1.70012238046265	5.26050286246598
H	4.39231435161047	1.68139991030579	4.08883464685542
H	4.50803355662020	2.30952278061650	6.15313567936310
H	3.73805601605450	0.35245002069920	5.65639307632637
H	-0.49306076140886	-0.32015763704564	1.29803032099548
C	-0.90656718720646	-1.30967238782011	1.16093781085787
C	-1.98397153549482	-3.84438652469257	0.82217905285276
C	-0.29897266400261	-2.40548072732447	1.76717294602504
C	-2.04871013877953	-1.48049054840695	0.39462212113851
C	-2.59042722897329	-2.74907137595411	0.22365542185789
C	-0.84317182805162	-3.67141539546349	1.59392342107491
H	-2.51649974235073	-0.62455175898536	-0.06996048427883
H	-3.48287341368248	-2.88102623757474	-0.37075577374065
H	-0.38241958464188	-4.52027604696350	2.07804702202956
H	-2.40298681112151	-4.83238942493807	0.69791827839832
C	0.92269431499556	-2.21815572136040	2.62080473318565
H	1.76326303890707	-1.83500140758596	2.05003134611443
H	1.22415547247031	-3.15121767372155	3.08382531841416
N	1.33407571435712	-0.07141647667960	3.63734700396895
H	0.31933961433110	-3.79095169333611	5.21982455598142
C	-0.74226388163476	-3.61231309836908	5.13821321399008
C	-3.47528040018454	-3.14445244600707	4.96816231492009
C	-1.21194985413789	-2.33183328946023	4.85148262034923
C	-1.63713302443559	-4.65245553036121	5.32827908582926
C	-3.00372474971252	-4.42245098308764	5.23849616460309
C	-2.58471061551795	-2.10013702361244	4.77755369255338
H	-1.26700577509745	-5.64276194272442	5.54921149857636
H	-3.69949939874359	-5.23625561177215	5.38162491179478
H	-2.94592528882268	-1.10830203865719	4.54977700575388
H	-4.53751130256383	-2.96105984728546	4.89986064423365
H	-0.76131247225956	0.43302898406070	5.99796008943950

PATH 3’i_b

65

TS1

N	0.68084117919229	-1.22634314557529	3.66637312743009

C	-0.29182353010843	-1.22898288307070	4.61417942067556
C	-0.19858751610456	0.01248072184847	5.18908404627970
N	0.79761957836077	0.68580191638476	4.56220003904039
N	-0.47362217176376	3.13697382686466	5.47365509415082
N	-1.46399295849475	2.77894755812128	4.91212697518881
N	-2.45345977212629	2.46758669339316	4.40376077312941
Ru	1.49455136916723	2.54007399721728	5.12651681016757
N	-0.13508779153074	6.56582769189957	4.84810392023178
N	0.38658793466840	5.74422405228752	3.92416107608277
N	2.15941588581447	4.31614947807087	5.91583700479910
N	2.37945957441204	5.43005047230069	5.19032145322993
N	1.95086984176278	3.18351155525157	3.30814527506729
N	2.18310427458874	4.49324018299734	3.00897616104100
C	1.83271896021739	5.57761557227066	3.87605869232937
H	2.28816289258188	6.46763400321084	3.45184552629113
C	-1.42871787093419	6.62463955612010	4.54333752598729
H	-2.09015168606260	7.21638188379132	5.15204413557097
C	-1.73037853289187	5.85574712654838	3.41051936070405
H	-2.68587199463405	5.70455707902463	2.94468576288310
C	-0.53266574527074	5.30360897597330	3.02962623680878
H	-0.28093646768019	4.63883667734444	2.22624754893483
C	2.61585189654101	4.58139107636089	7.13740818178495
H	2.58622245670226	3.81873872911689	7.89370983127984
C	3.11534685609171	5.88652529628280	7.20142406977396
H	3.54724297035437	6.37739247344223	8.05220857416251
C	2.94399243563482	6.40592267803665	5.94513400042220
H	3.15848294721714	7.36961186256783	5.52343047697119
C	2.22313363647475	2.50354163602111	2.19275401128626
H	2.14881064473125	1.43319443323051	2.20193760788914
C	2.59894647523788	3.37325223115746	1.16737479266030
H	2.86823670047682	3.10932540077217	0.16286158279833
C	2.56126059982930	4.62768852481398	1.71027672026117
H	2.75123503634176	5.60169143896597	1.30006358275246
P	3.59704747068066	1.70012238046265	5.26050286246598
H	4.39231435161047	1.68139991030579	4.08883464685542
H	4.50803355662020	2.30952278061650	6.15313567936310
H	3.73805601605450	0.35245002069920	5.65639307632637
H	-0.49306076140886	-0.32015763704564	1.29803032099548
C	-0.90656718720646	-1.30967238782011	1.16093781085787
C	-1.98397153549482	-3.84438652469257	0.82217905285276
C	-0.29897266400261	-2.40548072732447	1.76717294602504
C	-2.04871013877953	-1.48049054840695	0.39462212113851
C	-2.59042722897329	-2.74907137595411	0.22365542185789
C	-0.84317182805162	-3.67141539546349	1.59392342107491
H	-2.51649974235073	-0.62455175898536	-0.06996048427883
H	-3.48287341368248	-2.88102623757474	-0.37075577374065
H	-0.38241958464188	-4.52027604696350	2.07804702202956
H	-2.40298681112151	-4.83238942493807	0.69791827839832
C	0.92269431499556	-2.21815572136040	2.62080473318565
H	1.76326303890707	-1.83500140758596	2.05003134611443
H	1.22415547247031	-3.15121767372155	3.08382531841416
N	1.33407571435712	-0.07141647667960	3.63734700396895
H	0.31933961433110	-3.79095169333611	5.21982455598142
C	-0.74226388163476	-3.61231309836908	5.13821321399008
C	-3.47528040018454	-3.14445244600707	4.96816231492009

C	-1.21194985413789	-2.33183328946023	4.85148262034923
C	-1.63713302443559	-4.65245553036121	5.32827908582926
C	-3.00372474971252	-4.42245098308764	5.23849616460309
C	-2.58471061551795	-2.10013702361244	4.77755369255338
H	-1.26700577509745	-5.64276194272442	5.54921149857636
H	-3.69949939874359	-5.23625561177215	5.38162491179478
H	-2.94592528882268	-1.10830203865719	4.54977700575388
H	-4.53751130256383	-2.96105984728546	4.89986064423365
H	-0.76131247225956	0.43302898406070	5.99796008943950

65
INT1

C	0.97217121539512	-1.84484570166553	1.39367767146598
N	0.80080689924002	-1.16073306366447	0.27089365780874
N	-0.67371387259349	1.37231215519839	0.84301257578379
N	-0.99823775433600	1.43788189765185	-0.30483502443013
N	-1.33289819299626	1.56308001308105	-1.40024217122361
Ru	1.23777475057959	0.68768945152385	1.28493824041748
N	-0.39605648960674	4.63103789410300	2.11750618382263
N	0.33811434648264	4.34530816341802	1.02912784978300
N	1.58501206784512	2.14895769953557	2.69340967760970
N	2.01902876674690	3.40724771203157	2.42480646586020
N	1.89610560020124	2.12394140411172	-0.25415663364012
N	2.26934758185811	3.38865051702724	0.03313857588292
C	1.76309733404489	4.08141831127687	1.18337053121588
H	2.30676184096938	5.02002409369461	1.23746806978296
C	-1.58409011427838	4.97748584762795	1.63116010493919
H	-2.37784229192196	5.23952144072704	2.30901232699405
C	-1.60455023057280	4.93532596809727	0.22957327792178
H	-2.42359208565170	5.15138437601673	-0.43055537797958
C	-0.34168351135084	4.53498074027910	-0.12927543686034
H	0.10042188074074	4.35581521741471	-1.08998267307005
C	1.73639710815172	1.99075768672677	4.00680567606199
H	1.50603463813834	1.04679740482218	4.46165322785445
C	2.23949070168194	3.15280529396094	4.59442939187097
H	2.45738127503808	3.31634091933709	5.63218288514884
C	2.39723300846943	4.04000363601206	3.56470715253452
H	2.72617016518632	5.06183383299770	3.53755041997921
C	2.37794505883078	1.87544792570778	-1.46844312979713
H	2.23351228687693	0.90312442573661	-1.90269465405825
C	3.05069025858976	2.99040126119769	-1.97538001697725
H	3.53725278875216	3.08648199927293	-2.92690937064590
C	2.95842054849929	3.94471625128082	-0.99413738674585
H	3.30167392829460	4.96124848325089	-0.94137838738879
P	3.50978058068866	0.17933336245068	1.39969296090395
H	4.14184092908382	0.34237396790864	2.64695080744990
H	3.89323195645871	-1.13267005277076	1.06210737329846
H	4.34937280896023	0.93749896363008	0.56415820465650
H	-2.66348242491917	-1.14891927959251	-1.91525483625015
C	-2.78909535380632	-1.21623989848769	-0.84462779675473
C	-3.13503554450870	-1.40362514171923	1.90011201432735
C	-1.99481413773109	-2.09398784914227	-0.11260491850720
C	-3.73486013811145	-0.42528237925121	-0.20916253018521
C	-3.90614261027995	-0.51078497982521	1.16474994767383
C	-2.19041182812368	-2.19059624400085	1.26258743173499

H	-4.33703445653251	0.25852180750998	-0.78935793818642
H	-4.64263265232270	0.10585869928254	1.65941208343873
H	-1.60474763630883	-2.89228820332722	1.83654162451474
H	-3.27148485361562	-1.48801703707373	2.96861855663336
C	-1.03001708170750	-2.99819777998265	-0.82221799831683
H	-1.54885402366941	-3.56049326349919	-1.59280586187933
H	-0.59786869041171	-3.72270668478167	-0.13180156540126
N	0.10463981420778	-2.36788522050816	-1.55372265943813
N	0.89770137516669	-1.58861123947319	-1.02225017257613
H	0.35785002957285	-3.30073715193157	3.52192148420086
C	0.89336082714364	-3.86586627689327	2.77429820909204
C	2.28411339667135	-5.31587313523526	0.85511627596125
C	1.27272845740867	-3.25003946684854	1.57467038658102
C	1.17866772839458	-5.20118420441972	2.99132553179074
C	1.87437347919218	-5.92904474285781	2.03381367011419
C	1.98352597579560	-3.98723780265296	0.62078052467497
H	0.86562760701445	-5.67363811155892	3.91037411424213
H	2.10482472557928	-6.96960963990521	2.20871818862822
H	2.32209902081390	-3.51514543141106	-0.28740924398772
H	2.84262086204850	-5.87517851901479	0.11947989787062
H	1.12086300749922	-1.04037649751303	3.45802384809355
C	1.02076934304299	-0.81251382489467	2.40297868567954

65
TS2

N	0.53606825917323	-2.56606148585701	2.71526285696686
C	-0.82828441062532	-0.92519081646465	1.19366925284025
N	-0.06775119090715	-0.62076016461210	3.51574830050697
C	-0.51192547082964	-0.17355828263227	2.36035475213547
H	2.99108447765322	-3.56721442559920	1.80835238448526
C	2.44043094398966	-4.31986723728114	1.26186249747770
C	1.02411688213903	-6.24333427589876	-0.14768522876912
C	1.15869790180267	-4.67211710345386	1.67838963829411
C	3.00770634934744	-4.92138687115028	0.14949097099433
C	2.30246897241590	-5.89021634798938	-0.55460725575371
C	0.45364703245474	-5.63180027607723	0.96165233806526
H	4.00133791242659	-4.63841911659088	-0.16691572457955
H	2.74511993135692	-6.36022682900739	-1.42090742823734
H	-0.54688544460374	-5.89948848911401	1.27179821950853
H	0.46638105015700	-6.98836768230879	-0.69645729228179
C	0.55101071541824	-4.02315006092765	2.88317990683871
H	1.13622875779032	-4.24843118857723	3.77663097945770
H	-0.46280440164814	-4.38166769185127	3.05620497229879
N	0.02948711901496	-1.93344194443364	3.65271498250551
H	1.33261638008267	1.59813771654493	3.25848690261992
C	0.59817042801240	2.05903228547879	2.61682980157758
C	-1.30976522868063	3.23318722713674	0.95185986577132
C	-0.43744276722526	1.27085084471551	2.08237809291083
C	0.67371694708465	3.39451156883840	2.30426054226457
C	-0.27435234669358	3.98476511409141	1.45796370424684
C	-1.41353652228858	1.87007575211770	1.25897545371372
H	1.47447668190578	3.99445227997769	2.71053237990350
H	-0.20051118594508	5.03591842670033	1.22204181617866
H	-2.33765679179858	1.35954072361013	1.05056263704270

H	-2.07019384576792	3.69110788435429	0.33760323953188
H	-1.30106413770716	-1.90068705663805	1.20733098938068
N	-2.41262872366045	0.22731323433094	-0.94876842272819
N	-3.04636347791000	-0.78061377480541	-0.87491025493777
N	-3.70469426382594	-1.72894101224992	-0.82243684149190
Ru	-0.39059775432572	0.20637123108381	-0.25175787061396
N	-2.13616215832711	1.32967780568168	-4.14477773690283
N	-1.61743680684457	0.12928274607732	-3.83907896683889
N	0.15968925082608	1.61596433533732	-1.87712117885566
N	0.38868177894501	1.20113341500350	-3.14042449097034
N	-0.05052987678867	-1.31540935937726	-1.56767931180366
N	0.15180286433762	-1.17359968466865	-2.90451221033510
C	-0.17755945207434	0.00039012669232	-3.66894206523260
H	0.28823589532801	-0.14596524649085	-4.63963660685828
C	-3.41363657230895	1.07626983453018	-4.41400269377743
H	-4.07084272961758	1.88815909432458	-4.67297027527638
C	-3.70696208793360	-0.29061793963255	-4.30437872377327
H	-4.65028128308586	-0.78264122794906	-4.45038865227780
C	-2.51964579791456	-0.87712655087982	-3.94324222419630
H	-2.26584486517970	-1.89811086964967	-3.73283669693272
C	0.70396560120917	2.82874372523473	-1.80458587842564
H	0.69004734359137	3.35959320882527	-0.87224052810800
C	1.25871668957480	3.20617153771797	-3.02826859654677
H	1.75948867069636	4.12511447843695	-3.26522851091716
C	1.03489209405299	2.14335083775669	-3.86544423228591
H	1.26793378340324	1.97746913381100	-4.90072567303558
C	0.23139115417092	-2.58854862363955	-1.29283089224478
H	0.19464316568768	-2.94049461026771	-0.28097873270657
C	0.59155883697458	-3.27752526112959	-2.44955274879510
H	0.86824583883917	-4.31007759497549	-2.52658129043398
C	0.53185436658333	-2.35413192156593	-3.45677414277957
H	0.70864284896770	-2.42723558896586	-4.51364033091117
P	1.89729740436875	0.24848044373509	0.19702224431182
H	2.53743126885897	1.49863325776156	0.29688460721238
H	2.33359329061332	-0.37876623101244	1.37986200642056
H	2.71015070526331	-0.39284542618160	-0.75850662484701

65

INT2

N	0.16741758503257	-2.25891704329746	2.33146741087243
C	-0.62594248108025	-1.34046881336638	1.72365254111847
N	1.05803067967477	-0.38480177525533	2.85018395380622
C	-0.05756502241568	-0.11791293471359	2.08032220714930
H	2.66712113267143	-3.75041731199459	2.17291554611227
C	2.16841353759168	-4.43547113791243	1.50333246334017
C	0.89075415752702	-6.19683336540658	-0.21953644599528
C	0.77924614691633	-4.53123432673691	1.51956299220504
C	2.91092151550311	-5.20401263344814	0.62019116640715
C	2.27363973564419	-6.08733184628434	-0.24254727938559
C	0.14643173210018	-5.41836335481587	0.65610649787409
H	3.98758565893188	-5.11661162798093	0.60810335250888
H	2.85328117483995	-6.68497470713711	-0.93088076432775
H	-0.93194842072987	-5.49058611676081	0.66094540516976
H	0.38912309319026	-6.87884228233443	-0.89049155836907
C	-0.03952413317417	-3.70115392415695	2.46613594190529

H	0.19910659367426	-3.93839494002108	3.49954135650195
H	-1.09862964567918	-3.87858851581359	2.31524154638595
N	1.16678278173538	-1.65808132049153	2.99447396519914
H	1.42732618923113	2.07979047006079	1.59423088640038
C	0.37240619466991	2.21958423702347	1.43806580688099
C	-2.35797882012074	2.79073355900374	1.59932255024090
C	-0.54596816302717	1.21739638151611	1.80739833461498
C	-0.10671915951797	3.50515863256222	1.13672144606317
C	-1.45035446724406	3.78218790411026	1.19752736588826
C	-1.91871901305771	1.52909006023422	1.89813944865841
H	0.59695489842945	4.28881222169362	0.90005091549664
H	-1.80822723085045	4.77638900007679	0.97442142276545
H	-2.61105282716191	0.76423548730704	2.21402274508703
H	-3.40889731820179	3.02585662638586	1.67916942017501
H	-1.56958801084879	-1.61589325222193	1.29468237032870
N	-1.85906965551624	0.42895272989770	-0.70420985889333
N	-2.64367218059619	-0.43878408281130	-0.50400833991549
N	-3.45054576822125	-1.25233360423885	-0.31528078881460
Ru	0.21989636586453	0.26384088720557	-0.19493828835626
N	-1.98150317032622	1.46083581954280	-3.86179031758605
N	-1.52360304633236	0.22979947738983	-3.58597881687632
N	0.67079490694247	1.49523268802777	-1.76808577077316
N	0.62504441104027	1.13905538421814	-3.07336381089544
N	0.35616066743022	-1.29528624417903	-1.51243397545320
N	0.25771633189562	-1.19768456362936	-2.86291943930099
C	-0.08621660030564	0.00294256129386	-3.56658227471846
H	0.26047539445970	-0.13954417108552	-4.58679662347412
C	-3.29523942178121	1.29950287696567	-3.99660404387188
H	-3.91513721064329	2.15427804298829	-4.20445877624819
C	-3.67320935296596	-0.04067088538112	-3.83294989679583
H	-4.65942764977619	-0.46364698274248	-3.87425926502530
C	-2.50050500749253	-0.70906230810275	-3.58132950331638
H	-2.29908234280754	-1.74268067276988	-3.37583393667702
C	1.32823439302490	2.65657235093223	-1.74173622118395
H	1.54962314297723	3.12547628543392	-0.80646479010673
C	1.67341806708787	3.06251167795462	-3.02934583363850
H	2.19862634808586	3.95509142139205	-3.30918775040372
C	1.20974332946709	2.07673386878465	-3.85719207098971
H	1.22701209106262	1.95944531679756	-4.92425214119962
C	0.67596694048102	-2.56432137434967	-1.26963007122791
H	0.85296670089798	-2.88674327453551	-0.26484502914588
C	0.76636150234436	-3.29268247880123	-2.45428587941404
H	1.00264136967859	-4.33315195983351	-2.55740336949518
C	0.50367643164913	-2.39462974140160	-3.45215282906093
H	0.45761393189402	-2.49351036033730	-4.52077762689032
P	2.47188407840205	-0.00730888075698	0.10771648591104
H	3.24359911563882	0.85487187982439	0.91573167556734
H	2.89173459381170	-1.25450517662311	0.61502035743582
H	3.22626319837483	0.03672014310617	-1.08373419024396

PATH 3'h_a

61			
TS1			
C	1.04683905107399	-2.25998661945339	0.25636089359455
N	2.27116349417015	-0.92666108865437	0.02712772393688
Ru	0.34110095482071	-0.19913939772904	0.02641570750239
N	0.43151225326463	-0.25866671123307	2.10101522208203
N	-0.23418361132682	0.46785359417335	2.77789879525468
N	-0.83332589611957	1.14684300225166	3.49386364125032
N	0.23240493138711	0.13485705684055	-2.04447093001157
N	-0.53901943109338	1.16662536533505	-2.45667744830547
N	-1.65285630405886	0.27787915536167	0.08446002516142
N	-2.09074031379275	1.31158335528902	-0.66834603195493
N	0.62694205055828	1.86783263390024	0.09149721618752
N	-0.14753259154753	2.65758797164683	-0.67196113569663
C	-1.15576336259758	2.03802722335484	-1.49120522121405
H	-1.69657776363203	2.81133004315931	-2.02263812386467
C	0.62855679991480	-0.48379999295846	-3.15267169736995
H	1.25721197233058	-1.35025621632554	-3.08860154041003
C	0.11512535621200	0.15654386940700	-4.28087322689162
H	0.28144620950821	-0.10626260659277	-5.30700373627790
C	-0.62709320132869	1.20709314609975	-3.80713592237922
H	-1.19103052122904	1.97782019633553	-4.29777409499732
C	-2.71322006723185	-0.16867544668163	0.75050937399732
H	-2.60947369433452	-1.00589042757793	1.41456184810928
C	-3.84232850478383	0.58689177828115	0.42297412623860
H	-4.84007118152133	0.45944213979147	0.79665528930715
C	-3.41571915401254	1.52583200916412	-0.48315081901397
H	-3.92451202301082	2.31403795909050	-1.00546069545524
C	1.49745919405379	2.67024093503689	0.69660061929983
H	2.22518804403813	2.25850484302429	1.37015138225226
C	1.27427818051384	3.99501507522366	0.30930555475433
H	1.81322255736149	4.86522163525008	0.63144606114980
C	0.21989245968912	3.95671187279494	-0.57049645557430
H	-0.29348270204719	4.72083439550350	-1.12307343426654
H	3.71638604957707	1.54725313610193	-1.55824355983457
C	3.57501786361177	1.48375807391342	-2.62711503916978
C	3.23388983607259	1.35552076750285	-5.37497455305625
C	3.99620845133077	0.34846546094263	-3.31812948435783
C	2.96640772014975	2.53197845327654	-3.29907005586666
C	2.79670797951595	2.47129084350503	-4.67649283064271
C	3.82286051372181	0.29686563754072	-4.69696701770663
H	2.63199767820991	3.39876241771330	-2.74749694913927
H	2.32211315583043	3.28827479766027	-5.20054851094177
H	4.14440115448280	-0.58040380357094	-5.24097790826607
H	3.10386925432450	1.30100690049535	-6.44626535465280
C	4.58356739717152	-0.82095419820177	-2.57987817336093
H	5.38343936854790	-0.50431216396679	-1.91083897044372
H	4.98519524668936	-1.55111742884561	-3.27324070502536
N	3.55996882051378	-1.55195526544265	-1.78190296510418
N	3.05221040020101	-0.87260945958048	-0.89024071968158
H	0.17205789602637	-4.73130381781883	-1.00829660357805
C	-0.68976586640213	-4.27702465570178	-1.47332428266133

C	-2.88741742880928	-3.07352732919667	-2.67999176063242
C	-0.91822383927203	-2.89945222138995	-1.29449659649516
C	-1.55787066379047	-5.03426703631262	-2.23336297013618
C	-2.65931994624640	-4.43574073913044	-2.83779836184292
C	-2.02376385330639	-2.30774811804500	-1.92114349729281
H	-1.37683699978091	-6.09103313377682	-2.36260793218288
H	-3.33427687018504	-5.02979499974301	-3.43638095992610
H	-2.19585585282654	-1.25240195268197	-1.81269291969957
H	-3.73656228082975	-2.60788533459543	-3.15753833285485
C	-0.00251081395689	-2.16227121124642	-0.50340673755117
H	1.29643244420000	-2.71546436851412	1.19756078570904

61
INT1

C	1.25327827714550	-1.70539386699960	0.21854456511010
N	2.08925973734444	-0.63619443887131	-0.06742057251315
Ru	0.03662090759564	0.00092673261169	-0.04536906389530
N	0.20877898535578	0.06422064093658	1.99055908326266
N	-0.66548989038964	0.43605175891555	2.72197611828116
N	-1.43127633666470	0.79876981876777	3.50039789654234
N	-0.20923820015539	0.35333593967601	-2.11773170740732
N	-1.03745391225953	1.35637225338295	-2.48930624745326
N	-2.00675625501131	0.35024824346865	0.04229195409533
N	-2.53324180512287	1.37520378531229	-0.66002122071177
N	0.19137128458623	2.14447357813462	-0.00958167995889
N	-0.69415770777849	2.87133838175193	-0.71220403028573
C	-1.68007815223486	2.17982488025603	-1.49721765431835
H	-2.29516891795337	2.91139504424909	-2.00485617861123
C	0.12999198535137	-0.26809189543905	-3.24360481537117
H	0.77753711441419	-1.12069539949375	-3.21405026175120
C	-0.47330276395767	0.34358613959164	-4.34305880376044
H	-0.37212395117065	0.06824532765401	-5.37500475428595
C	-1.21616126973927	1.37586653675834	-3.83071646824745
H	-1.84291332248590	2.11758733841295	-4.28921667720272
C	-3.02659186088138	-0.22937337709160	0.66895422451951
H	-2.85158564154945	-1.08363494824940	1.29544447210621
C	-4.21955804496092	0.42876672371195	0.36105711167295
H	-5.20380425496533	0.18927804654761	0.71486580874229
C	-3.87444664658258	1.44743052226533	-0.49230992981874
H	-4.45068190949304	2.20764537696852	-0.98551079699051
C	1.00683087741302	3.01653990554836	0.57370839346479
H	1.80294986176422	2.66467675982729	1.20260434437244
C	0.63717961549747	4.32096303897165	0.23482867190030
H	1.10326431690059	5.23370200459227	0.55303756264363
C	-0.45240270973924	4.19650127071589	-0.59425394833647
H	-1.06619450654095	4.91881998248240	-1.09907489995608
H	5.79771801849366	0.10740546013179	-2.17718415490553
C	5.20338500299971	0.63921854775901	-2.90758446542923
C	3.68336020136568	2.00327478583419	-4.78193129831562
C	4.11210096650654	0.00330201171538	-3.49581434266102
C	5.52726819459292	1.94494532288312	-3.24399062541691
C	4.76556813879039	2.63234100377756	-4.18173002266137
C	3.36299630444050	0.69598283769044	-4.44126778643152
H	6.37653506184375	2.42556680927834	-2.77947647105088
H	5.01791868079497	3.64936483645253	-4.44567449178667

H	2.52384351108476	0.20857383295885	-4.91600250422154
H	3.09147348444329	2.52712241943802	-5.51907121308108
C	3.74332660660463	-1.39176739250258	-3.09736906058905
H	4.63450722615339	-2.00814008269558	-2.97183031804187
H	3.14902176496724	-1.87399476634429	-3.87462056490607
N	2.97769769415974	-1.56347585684567	-1.84843804518869
N	2.69102882485061	-0.50094975210137	-1.26683483046461
H	0.98046761529536	-4.29320737346918	-0.98588817521961
C	-0.01088822387897	-4.12846057586841	-1.37980835696600
C	-2.55090088180806	-3.64345952042299	-2.42299066080034
C	-0.61737172075284	-2.86719186645403	-1.20489930630563
C	-0.68018233526326	-5.13032672474951	-2.04976756283079
C	-1.94831337721055	-4.89021948124288	-2.57220276727979
C	-1.89654600750501	-2.63685574724511	-1.74611099557266
H	-0.21731561336191	-6.09738095283512	-2.17692601572731
H	-2.46576747672280	-5.67523442722401	-3.10396675136325
H	-2.35167825897001	-1.66716767409900	-1.63408566059870
H	-3.53053275638673	-3.46458410025104	-2.83989914130334
C	0.07658045437234	-1.85856301666705	-0.52148934731058
H	1.33600399636876	-2.13532466226808	1.21253444059171

61

TS2

C	3.95551798010099	1.52840284282347	-0.22882505088458
N	3.15384602019170	1.33417169469334	-2.38349772689390
C	2.89288117949646	0.82542683528858	0.08252047677429
N	1.88085614519834	0.44830497526919	-0.83678422573056
Ru	0.19228645502899	-0.54198450074028	-0.25592389479381
N	-0.89971378791666	0.32721839726112	-1.75386229868258
N	-1.75795831990609	-0.46283783206319	-2.42923923394458
N	0.49299206701846	-2.00981291480920	-1.48960267438955
N	-0.54146056911373	-2.47686259459740	-2.22234584896769
N	-1.54536918161670	-1.48372834752799	0.28236811826145
N	-2.29549293183063	-2.00242203226369	-0.70912465424771
C	-1.83421972599463	-1.85575240737245	-2.06880271063184
H	-2.53780790228098	-2.35309797377839	-2.73285072134228
C	-1.00294921388129	1.54203935736795	-2.29980044889646
H	-0.39343629814434	2.35717582240720	-1.93895662078726
C	-1.95133475792970	1.52505867715106	-3.33816887598330
H	-2.24943094262244	2.35365833488286	-3.96168048328587
C	-2.41600928370030	0.22656809550624	-3.39934496588867
H	-3.14474176117585	-0.25807746015270	-4.03240851705094
C	1.56516262385807	-2.73416968754544	-1.82719590457589
H	2.51828094216070	-2.53724351945758	-1.36033084616326
C	1.20212265032615	-3.68550824134625	-2.79654848419391
H	1.84476004979730	-4.41686871743565	-3.26293784116382
C	-0.14585087608062	-3.49817170838117	-3.02836029397331
H	-0.84795515751752	-3.99469838905496	-3.68176596958231
C	-2.18590550755017	-1.76516466583574	1.42010480914790
H	-1.77178117381436	-1.44929954517544	2.36657051352810
C	-3.36829958964195	-2.47496044846711	1.14643189158370
H	-4.09429233171381	-2.83880677221201	1.85776017006987
C	-3.40971399436791	-2.61283460007571	-0.22721342525077
H	-4.12141788717319	-3.08216880525307	-0.89044230486416
N	1.17435950881370	-1.24068080881638	1.39033417756888

N	1.08692389123825	-2.31520831126262	1.91374941148244
N	1.04656426866567	-3.33370672106570	2.46426624615741
H	2.67988798018960	4.14778042898978	-1.94181709350319
C	1.99280593902805	4.10087950536279	-2.78218241756396
C	0.22062113644438	3.96889496348290	-4.93023015769257
C	2.16440763461488	3.11244114911010	-3.75969527473655
C	0.94138907245848	5.01066633149203	-2.87442786649754
C	0.05107441661627	4.94652424492486	-3.95029891098650
C	1.26984096535335	3.05268848372147	-4.83124267120743
H	0.81626715631425	5.77094879631831	-2.10910670062290
H	-0.76946088549799	5.65411848249816	-4.02153006370934
H	1.38818505631576	2.27987088987872	-5.58576271494754
H	-0.46913695443816	3.91033465187414	-5.76680015372622
C	3.26626266701452	2.09287573764536	-3.61361220817510
H	4.24884595577698	2.57074194662604	-3.58383843953191
H	3.27042091747571	1.39972715499816	-4.46232325873274
N	2.07214168450697	0.79179330891528	-2.06648574233426
H	4.38477108942416	4.13333184822456	0.11761893087075
C	5.29565414860917	3.54467435620223	0.10497529626806
C	7.62749461276352	1.99020221679224	0.04856694470579
C	5.20619968049685	2.14280076629276	-0.07598043681002
C	6.53541690577969	4.14738316727759	0.26677222776688
C	7.70112109042752	3.37468712080399	0.23679317099894
C	6.39438318875517	1.37228537549259	-0.10549761389803
H	6.59870742562408	5.22094258781726	0.41210190160024
H	8.66840732012257	3.85198822007809	0.35880230354223
H	6.32355989975804	0.29977182860206	-0.25074929286247
H	8.53528734089415	1.39589548866849	0.02564698071411
H	2.69122883943857	0.46956599535926	1.09108866983285

61
INT2

C	2.33608981217338	1.88224944479408	-0.96651989768094
N	1.58714952279306	2.54877277031632	-1.86068337058200
C	1.50014262328001	1.74419754378687	0.14231437681688
N	0.28771635869156	2.31242214494136	-0.17840448854409
Ru	-0.08880320915512	0.03235642629973	-0.03935558512539
N	-0.31057269375790	-0.16056736446271	-2.09703674961208
N	-0.17375872654841	-1.40999861930408	-2.60298589323130
N	1.37844816522698	-1.26797300911918	-0.13880415065875
N	1.26998044536299	-2.32717116088808	-0.97295646028175
N	-1.29783989033939	-1.56452193168272	0.05774055982433
N	-1.05804048997513	-2.60610490611073	-0.76956344843963
C	0.04227414196077	-2.50489529144053	-1.69745313173740
H	0.10241511940354	-3.42410243059239	-2.26638111626592
C	-0.72764046711254	0.58997477037830	-3.11811805596516
H	-0.91118023825751	1.63582723022210	-2.96729524326929
C	-0.85172681874272	-0.18148055871936	-4.27781163744889
H	-1.15217669666770	0.15597180431174	-5.25127648666736
C	-0.50631182518874	-1.45818616090673	-3.91646883878262
H	-0.46137993703104	-2.38159898279445	-4.46275805730597
C	2.55388213449899	-1.39603519818042	0.47742771466515
H	2.86953465573030	-0.66963462545316	1.19946086122372
C	3.20261688961500	-2.54717629300324	0.02545819694919

H	4.16685481643064	-2.90457490380659	0.33164568676778
C	2.36135578975748	-3.12553151068071	-0.89149906834109
H	2.43638806041265	-4.01669968542617	-1.48550139311891
C	-2.35410352376119	-1.90948179553154	0.79384939541599
H	-2.73334852849483	-1.23328707351653	1.53649306769707
C	-2.79729645043756	-3.18190300699033	0.42379686676704
H	-3.62771660332815	-3.72287186827884	0.83526906964760
C	-1.95190681864897	-3.60336306094486	-0.57384478647902
H	-1.90777733543436	-4.50687357938156	-1.15228251620055
N	-0.10260254105697	0.20620233848415	2.01748944851699
N	-0.00504245402073	-0.71074596297858	2.77801805543467
N	0.08358971585647	-1.54799126086033	3.56920709267614
H	2.96597154111985	5.26193223248899	-2.16214396823446
C	2.14892471153987	5.45742426118863	-2.84299033818562
C	0.05390767380874	5.95288837594837	-4.59481987159251
C	1.50949403240186	4.39419483808770	-3.47435368921274
C	1.73911504327380	6.75980857108551	-3.08154685594585
C	0.69093429001499	7.00991352200321	-3.95975856095809
C	0.46138755076531	4.64830121190711	-4.35022266805914
H	2.24017769712354	7.58027246741737	-2.58854051022646
H	0.37388689322430	8.02546686066315	-4.14792715182382
H	-0.03688428541231	3.82262060685143	-4.83840288652509
H	-0.76073531174053	6.14234898629641	-5.27867690408273
C	1.95243121452961	2.98805135694328	-3.21043585077306
H	3.03166744488147	2.89731491302508	-3.27449043634810
H	1.50907697302573	2.29551402987626	-3.92041165878144
N	0.35468048841176	2.81057881481357	-1.38940967986471
H	4.41994540641053	2.12816216680811	0.67891094587296
C	4.64613268770045	1.53840595749296	-0.19683506289485
C	5.21269584372924	0.01472836561336	-2.44983634803032
C	3.67002404050715	1.35607495715748	-1.17725304142227
C	5.89845122866507	0.96834855478326	-0.35123810985943
C	6.18512421210153	0.20730840069267	-1.47750229998511
C	3.95871863182381	0.58394260671236	-2.30362878136984
H	6.65144924979619	1.11769077843382	0.40843318898562
H	7.16132581136567	-0.24019171574743	-1.59333350928712
H	3.19242359363764	0.39650219648470	-3.04051078323137
H	5.42656382151020	-0.58945520275396	-3.31907634527753
H	1.78109338473894	1.52442072865632	1.15370836161429

PATH 3’h_b

61
TS1

N	1.63110069983301	-1.49666589334131	-0.15987385761978
C	0.27574244701439	-2.51627609391296	0.54234398180394
Ru	-0.00321985939856	-0.26143249312230	-0.04260755923348
N	0.56089498224365	0.15858195205818	1.88071639947907
N	0.14499160824856	1.10241862418602	2.48898087884860
N	-0.19014301529667	1.99630134266992	3.13483163769585
N	-0.49985461792628	-0.42612845013775	-2.09897785169773
N	-1.11435228570920	0.63334294674352	-2.66801981171067
N	-1.80605561537487	0.69692908077675	0.15919743126782

N	-2.17911541619459	1.59193921840270	-0.78089034529818
N	0.71388151307126	1.61638774743606	-0.67207240880137
N	-0.05346424964627	2.36976587547030	-1.47711314339870
C	-1.31913733755608	1.84000344077548	-1.90964731894308
H	-1.79785051048042	2.57020683417413	-2.54868814682315
C	-0.48972096718473	-1.37882153429989	-3.02621221842044
H	-0.04686259650751	-2.33203665124808	-2.81176536703551
C	-1.09555775567890	-0.92552670544135	-4.19887588169204
H	-1.21436265356299	-1.46496264484123	-5.11799408736789
C	-1.48586812523003	0.36365684240824	-3.94134780600779
H	-1.97400435313526	1.10836265730132	-4.54125607743362
C	-2.80039413242661	0.65279229213636	1.04060088894440
H	-2.72785604853285	0.00098253016434	1.89084266739197
C	-3.81988213640556	1.53237314953257	0.66367019498468
H	-4.74398370727480	1.71481046322153	1.17766636935733
C	-3.39707368981184	2.11695355325552	-0.50420365453098
H	-3.84301304826046	2.84695949983407	-1.15313016476132
C	1.84365998638227	2.29394631531466	-0.49236770208615
H	2.61818850720641	1.88729619260749	0.13037426627194
C	1.79583904406021	3.49890716789517	-1.19990925373874
H	2.55494445528405	4.25597717891941	-1.24630236885486
C	0.57376043384671	3.51663045560869	-1.82781545852218
H	0.10184133275203	4.22737821092078	-2.47976407739655
H	3.16663417873776	0.13360022261828	-2.61502631644188
C	2.81236847004552	-0.16163235107952	-3.59168167481238
C	1.91632156295204	-0.89811729753439	-6.10872302798963
C	2.86268146457817	-1.50201118276193	-3.97180225643938
C	2.29841175705265	0.79631363942048	-4.45103500904537
C	1.85095683540243	0.43013965702739	-5.71429104356707
C	2.41332649844856	-1.85917424794584	-5.23835388416101
H	2.25186811089397	1.82859365441350	-4.13520218858372
H	1.44973066346157	1.17635597365713	-6.38480300449923
H	2.44702575247657	-2.89636946714659	-5.54211891439094
H	1.56897786406508	-1.19030734455271	-7.08935062010010
C	3.34679134236575	-2.54946603192925	-3.00807994963193
H	4.30664924007723	-2.27054472066285	-2.57361587449738
H	3.46081831277073	-3.50528060962138	-3.50743119886427
N	2.36946945774339	-2.78421842879743	-1.91265744859922
N	2.22742298720343	-1.80920382575411	-1.16636938151722
H	2.72207674849376	-3.54594443831646	1.00838190496896
C	2.02478444057566	-3.71152234483243	1.81426203194437
C	0.21573987495024	-4.14468505735862	3.88420372213675
C	0.72150087453655	-3.21356935288977	1.71547546486297
C	2.41200191590685	-4.42632919029958	2.93372686444536
C	1.51096154951502	-4.64301951890401	3.96963567490689
C	-0.17860672765040	-3.42991092422180	2.76830066844932
H	3.41751745468400	-4.81449184221639	3.00098134913941
H	1.81844190231735	-5.19759662456616	4.84417229380282
H	-1.17749187673656	-3.02599238357812	2.70040048512368
H	-0.48168587166256	-4.30625030754785	4.69294598935358
H	-1.84839178806740	-2.38761773485993	-0.14322418223956
C	-0.84787588348568	-2.05365102522764	0.06600937157525

61

INT1

N	1.26509768841977	-1.20371380536924	0.33135767572529
C	0.51180488793780	-2.09483273084288	0.97190140695469
Ru	-0.18603813744456	0.26434973734444	0.30370105527333
N	0.67477598645953	1.15405665256820	1.91535684748023
N	0.41039564074460	2.27730396108156	2.24117424911106
N	0.23361216525440	3.35418167646067	2.60607892676175
N	-0.80291029940783	-0.36553092139624	-1.59667735860603
N	-1.44754290963826	0.52674466179074	-2.38025691639727
N	-1.91633226511702	1.34689820629104	0.38985853228562
N	-2.36362524907009	1.96459763226950	-0.72534646732471
N	0.55509039550218	1.97829386198363	-0.91095323487679
N	-0.31075375974779	2.51167752337404	-1.78940265911041
C	-1.60275787122042	1.89477027798551	-1.94509609139069
H	-2.14928790977505	2.42985518950287	-2.71141508017320
C	-0.68002985732883	-1.47230508336728	-2.32449306097386
H	-0.19447896531566	-2.33502621078258	-1.91039233105415
C	-1.23930121361579	-1.28566817810051	-3.58819231966462
H	-1.29144234936246	-1.99888097041301	-4.38797894699918
C	-1.71145939835594	0.00479977431963	-3.59852455196636
H	-2.20542611740036	0.58879636743953	-4.35234322729155
C	-2.81517353947963	1.60270175039299	1.33694076270403
H	-2.67400608204041	1.20553395984613	2.32466098925967
C	-3.84444593543987	2.39862742687355	0.82567964210086
H	-4.70552247352351	2.76492263054834	1.35090768692730
C	-3.53081795480553	2.61270590038966	-0.49348978100545
H	-4.02462209056780	3.15571130865933	-1.27737912251804
C	1.66938943312298	2.69167915471314	-1.02510253756520
H	2.51814224665093	2.45448347974170	-0.41051703648054
C	1.51585596843458	3.69010124560726	-1.99369444456101
H	2.24135937374905	4.41721726607151	-2.30476403421861
C	0.23658188784981	3.54404637914338	-2.47330698859424
H	-0.32017790050271	4.07081622486833	-3.22552076865368
H	2.95829802208203	0.44822462004691	-2.48449022717827
C	2.80741365475324	-0.14078629217787	-3.37755293968435
C	2.38692605918693	-1.65719750336254	-5.65953876249019
C	3.11607873710221	-1.49908785996956	-3.36770564266832
C	2.29593526214423	0.45625582699900	-4.51820691276038
C	2.08279176454561	-0.30276676385261	-5.66289906742706
C	2.89554673216578	-2.25466702579324	-4.51337065843658
H	2.05734232827604	1.51002471432310	-4.51091281590060
H	1.67809907916488	0.15921753920158	-6.55181814319073
H	3.12182232959433	-3.31168499263910	-4.50774981132421
H	2.22265609417460	-2.25162057264890	-6.54676062889832
C	3.59183517691127	-2.14038982510806	-2.08712362589443
H	4.39652527308946	-1.56929392485181	-1.62494859175599
H	3.91147886950568	-3.16263881033617	-2.27021651772462
N	2.41285607747046	-2.19393827086630	-1.22346211870633
N	2.43763260854308	-1.32783780065037	-0.33072140144754
H	2.99083945956329	-3.07166921787609	1.54309242090648
C	2.19526052656627	-3.78498937183753	1.69134262097643
C	0.15128781881998	-5.62099909433198	2.12301574755874
C	0.86874238325176	-3.42079251958556	1.43574607550665
C	2.48971594439861	-5.05311084887372	2.15567330478510
C	1.47113391522518	-5.97712784975354	2.36696682483573

C	-0.15226391338638	-4.34861282251946	1.67009875692523
H	3.51556032797564	-5.32338701639526	2.35824628394348
H	1.70685378793405	-6.96823579697311	2.72584088283050
H	-1.17900857135663	-4.07638979986818	1.47868027112449
H	-0.64287501889776	-6.33376105026037	2.28907926898952
H	-1.62282454352210	-1.74872919587519	1.55552071705556
C	-0.71011358024789	-1.35377282315875	1.12652387489212

61
TS2

N	2.19935853509353	-2.37453542850848	-1.76362294667021
C	1.30723235480412	-1.33233542083818	0.09261978553471
N	3.37060089436845	-0.67334099804946	-1.06095345197517
C	2.41148468526997	-0.48417700276118	-0.17637819545068
Ru	-0.10047044542200	-0.04078113807649	0.07537308405821
N	-0.19013608008184	0.19459343799108	2.14069125276915
N	-1.21894870356880	0.11806501004270	2.74159515234040
N	-2.17390747225036	0.07937485421182	3.39159593014410
N	-0.38980657964384	-0.07807987888772	-1.98298132765448
N	-1.65924450499106	-0.21750265382143	-2.42638963338286
N	-1.50961524958234	-1.46430785638886	0.17371037529499
N	-2.61709300160541	-1.35930604383382	-0.59166002980331
N	-1.79788370135948	1.36696517187361	0.01201871163202
N	-2.83503426548038	0.99420422291235	-0.76045458746679
C	-2.75429246671923	-0.24474664364860	-1.49124961744075
H	-3.67325708075553	-0.37178818351272	-2.04895326011373
C	0.37473989651615	-0.06105627368409	-3.07200458108853
H	1.43991403742358	0.03282905624808	-2.98823124041692
C	-0.41023204267128	-0.18288601182281	-4.22077382971201
H	-0.07230363913778	-0.20095252584967	-5.23897473240014
C	-1.70545126281007	-0.27716203186299	-3.77765309474160
H	-2.64278707307317	-0.37591456982024	-4.29201878477144
C	-1.69760270961046	-2.52209181784774	0.95901713977136
H	-0.93789250534939	-2.80961340946015	1.66016840762070
C	-2.94660571921501	-3.09082795923628	0.70235436975721
H	-3.36929201662010	-3.96054590762758	1.16644407825174
C	-3.51305806745349	-2.32730144638510	-0.28625653711067
H	-4.45063620866853	-2.39043328016483	-0.80537501032433
C	-2.17398932126978	2.49723920819976	0.59845553261870
H	-1.50543351595671	2.99296916800652	1.27708755266117
C	-3.46461711485934	2.85502977208967	0.19275106479551
H	-4.02883409531597	3.71692380058859	0.49382160111445
C	-3.86360578988737	1.87078492318240	-0.67860142021050
H	-4.76709646974814	1.71840282367320	-1.23890851406965
H	-0.26273140496052	-2.96544926909704	-3.34239209937606
C	-0.36726146711106	-3.79362434951466	-2.65669028258068
C	-0.63132974961968	-5.92471841842317	-0.90359664213498
C	0.76334991987242	-4.31360141316270	-2.03176166981336
C	-1.61807287817195	-4.33275346649535	-2.40764146017597
C	-1.75184037678544	-5.40469355378965	-1.53378870317081
C	0.62171979661253	-5.37610954295645	-1.14793740728204
H	-2.48837154579224	-3.91794459759080	-2.89486851892694
H	-2.72743189671207	-5.82523112686511	-1.33881241209859
H	1.49491973898264	-5.77989453188957	-0.65459062402594
H	-0.72989396069834	-6.75465658159599	-0.21883873304829

C	2.11383020544041	-3.72929873780088	-2.31291783068153
H	2.26288499251247	-3.63136595547628	-3.38983834961566
H	2.91530676779941	-4.34804403757354	-1.91444253165668
N	3.33184215910921	-1.89041878472895	-1.64043908390156
H	1.41132937821073	1.72872029466955	-1.40470685859551
C	1.54754007696584	1.84700065553096	-0.34383868362325
C	2.32809380450146	2.36872098127026	2.27893345720159
C	2.20798836206330	0.86243513839263	0.41206167184222
C	1.26495827242200	3.08740118087978	0.24505148121539
C	1.64020667941722	3.34077223533309	1.54433107681976
C	2.59790772512979	1.13794047631674	1.72842553688756
H	0.77105614945377	3.84695502939728	-0.34177992600663
H	1.42483935863610	4.29922941296765	1.99286062443281
H	3.10251295337198	0.37484882313949	2.30067997587439
H	2.64060559004706	2.58275991340982	3.29037543364760
H	1.29636804893434	-2.37214474127802	0.39172931523287

61
INT2

N	2.10508122168158	-2.44189473515760	-0.84835962105820
C	1.80967311377047	-1.53086718078931	0.12585209202572
N	2.70620090275714	-0.57578417883489	-1.70236319387951
C	2.21635554789285	-0.31212483651474	-0.44395359881160
Ru	-0.00598160027433	0.00648961130507	-0.08818924071725
N	0.13060924941262	-0.08043901238525	2.00072524624220
N	-0.83699181493569	-0.19848568480105	2.68943507214918
N	-1.72908471585088	-0.29648052978869	3.41951220802461
N	-0.41900125190057	0.02256335475894	-2.14841851956802
N	-1.72098580834801	-0.13258955266817	-2.48726532908654
N	-1.30868694713599	-1.54135943837069	-0.00068382854079
N	-2.46880228709977	-1.45508573454220	-0.68376485512533
N	-1.65103542023562	1.16853716655602	0.11660302619270
N	-2.74797638489432	0.88204041929861	-0.61393087375112
C	-2.72109716685831	-0.27820108512361	-1.46774938465466
H	-3.68733084773473	-0.37941411848915	-1.94493614907828
C	0.23090212832269	0.14486118228515	-3.30735758045316
H	1.29577035048353	0.27081976064552	-3.30975928823788
C	-0.65589233776407	0.06031533898302	-4.38362021467692
H	-0.41578088798340	0.11910981397901	-5.42785866642500
C	-1.89685759475229	-0.11759999818417	-3.82941224168829
H	-2.87492611564056	-0.23392902257805	-4.25693503395116
C	-1.37846431803648	-2.66912839995677	0.70276902302124
H	-0.56276715016837	-2.96547739499993	1.33228613432525
C	-2.59713428344570	-3.30654487238170	0.46708606716675
H	-2.92621753816341	-4.24194577482367	0.87537945032554
C	-3.27522799700963	-2.50872795165073	-0.41901719222491
H	-4.23590178385302	-2.59679644930981	-0.88973058398102
C	-1.96561197524542	2.22092046018903	0.86644513460412
H	-1.24000319062140	2.63447019045986	1.53979968635744
C	-3.28122405698471	2.61377411789028	0.60831958078178
H	-3.81293483183322	3.43142668800531	1.05577575663316
C	-3.75841833570374	1.73731445858270	-0.33588799148311
H	-4.70872936855289	1.64758045274576	-0.82760846910298
H	0.26226972513509	-2.63517208349242	-2.64984195057558
C	-0.11853398612649	-3.58774967945916	-2.31813295265635

C	-1.12652837702969	-6.02692241983848	-1.47194963260194
C	0.58162080127581	-4.32874992794403	-1.37473377109825
C	-1.31968920439082	-4.05732260977462	-2.82795179632550
C	-1.82520677991043	-5.28026055627536	-2.41040481305841
C	0.06796889108788	-5.55051749871213	-0.95263301982480
H	-1.86026118537900	-3.46177244160337	-3.54904807505588
H	-2.76272720867196	-5.64405102820009	-2.80508900779293
H	0.60236559031543	-6.12814526401702	-0.21090681381834
H	-1.51701718509290	-6.97587619909887	-1.13357707185973
C	1.91157262512183	-3.87922265479954	-0.82836862261777
H	2.72674125198944	-4.30489077619209	-1.40967698386849
H	2.03290633784058	-4.20719583359703	0.19978738902909
N	2.62722186060902	-1.84565053051702	-1.92039116776667
H	1.21156258404908	1.88121217191568	-1.64862070233316
C	1.39240113317649	1.99932852271123	-0.59525998890604
C	2.33591648133121	2.55034681223079	1.97296683434502
C	2.09189558722970	1.01315637344981	0.13593357498428
C	1.15520490288781	3.25073546705969	-0.00518706367287
C	1.60646903877187	3.51765442425382	1.26423672736296
C	2.56857562499791	1.31555924625121	1.42706710928705
H	0.63493543112092	4.00777071205168	-0.57225446884760
H	1.42587448179822	4.48424159791012	1.71102690666848
H	3.11569347741926	0.56277550749163	1.97229885344996
H	2.71405204683384	2.78144397665905	2.95789908226491
H	1.64771955031560	-1.83354237279755	1.14145480393463

PATH 3’g_a

62
TS1

C	2.91390065988682	0.34430608529481	0.59561527104162
N	2.10740709290178	0.69461851235608	-0.93149099000442
Ru	1.63427974462577	2.10552865645559	0.49364344909272
N	-0.37766836288150	1.41208821230334	0.79962708717980
N	-1.30070076505656	2.34968549393852	1.10364607635360
N	1.25858846832413	3.35269586931031	2.10698050442864
N	0.06120095807129	3.97626097543976	2.16622828194468
N	0.67131857176128	3.57671378578815	-0.63664063442537
N	-0.43473985694586	4.16092576443857	-0.13959339542127
C	-0.92669346624615	3.74032679272358	1.14403542246573
H	-1.80605977808090	4.32382102083543	1.38353945727051
C	-0.99876447901637	0.24322120413418	0.91801666251321
H	-0.47048445313134	-0.67114144295656	0.72792527448567
C	-2.32917176874917	0.43128998826509	1.29599919293053
H	-3.07338197186045	-0.32567634021339	1.45110769958693
C	-2.49305488860001	1.78821798053449	1.41043337649480
H	-3.33738647410780	2.39811326170680	1.67178577650696
C	1.93679636600139	3.75932861156381	3.17717056289350
H	2.92653894959225	3.38386573935078	3.35667420393242
C	1.16657347705259	4.64876627402750	3.92940934313539
H	1.44498822803168	5.13619325249092	4.84380846794057
C	-0.02638325190837	4.76802100230026	3.26104420343526
H	-0.91685598222038	5.33363883377638	3.46189433512040
C	0.83216382394398	4.06841876562473	-1.86211211357916

H	1.66213479718036	3.74899434328124	-2.46412735455084
C	-0.18928788666161	4.97598815251558	-2.15260532603616
H	-0.32493531905657	5.53150785905498	-3.06038542330469
C	-0.98992495156513	5.00774843156802	-1.03808272092894
H	-1.88759035505708	5.54867297955386	-0.80464658591767
P	3.64092950551871	3.17881136767363	0.17951401291017
H	4.53255547503598	3.21682786900186	1.26752935266853
H	4.48092080454863	2.70937366710152	-0.84915187112712
H	3.52883879231490	4.54294063321004	-0.14179304429371
H	0.48356842111909	0.39736831086786	-4.33954967836926
C	-0.45749256246632	0.55406592405756	-3.83104382590621
C	-2.87223721685437	0.95817705508628	-2.52778674824877
C	-0.91205577338870	-0.39340361300791	-2.91630069177816
C	-1.19845101198014	1.69882251695328	-4.08432487596905
C	-2.40444597840596	1.90640998164050	-3.42734888247470
C	-2.12938105733742	-0.18648019597397	-2.27605026688997
H	-0.83259467777546	2.43098825591716	-4.78947842732517
H	-2.97754982361146	2.80230506352424	-3.61758903776308
H	-2.48731759995035	-0.91779254136544	-1.56600968970637
H	-3.81158966984898	1.11130986447556	-2.01643059096025
C	-0.04640078782054	-1.55380937564173	-2.53649482642549
H	0.51472668528888	-1.93646528276118	-3.38915904093580
H	-0.62833562345592	-2.37276512904226	-2.12510149780531
N	0.94666720213323	-1.21280123160301	-1.47869558410141
N	1.31718024659142	-0.04387419338722	-1.47948481088633
H	2.96098071329424	-1.89621714757585	2.48214885650549
C	2.28653807062112	-1.41786265313235	3.17591168344902
C	0.52722675415907	-0.16115748669033	4.93181142543321
C	1.76384346687581	-0.14686923295057	2.86024164556961
C	1.93723105164258	-2.03568927027207	4.35770109085942
C	1.05874408156947	-1.40962090824698	5.23820565554075
C	0.86836381408654	0.46606784406871	3.75119888330501
H	2.34228374566932	-3.00757649397627	4.59663804062592
H	0.78375543074856	-1.89917980653046	6.16084754634027
H	0.44626892867238	1.42388286229092	3.50531452292752
H	-0.16104838484954	0.31574441632899	5.61310806307728
C	2.15081971685902	0.45459574259481	1.64737137647962
H	3.96960013476798	0.29408312190055	0.39876113068967

62
INT1

C	2.75926055792247	0.65008199479058	0.23756677330464
N	2.10303486549045	1.01926610182127	-0.93142879827479
Ru	1.64533176583160	2.39182240860243	0.65514386420379
N	-0.43456302540912	1.90367986739326	0.90837555053200
N	-1.27024944258253	2.91317895912594	1.23671933853541
N	1.32233090590287	3.56204545965564	2.35028660776989
N	0.21127752063428	4.32444005436168	2.42102701780125
N	0.82066870613312	4.04176294315461	-0.41525836517754
N	-0.21752481413043	4.71145228176630	0.12096466136418
C	-0.77132935812004	4.25836546686301	1.36893537422888
H	-1.59803381249340	4.90526136737151	1.63372855650948
C	-1.16345433883407	0.79480321306249	0.97850096866517
H	-0.71943554494948	-0.15888819911214	0.77558328223475
C	-2.47646468730908	1.09311467345479	1.34489964050939

H	-3.28930225436792	0.40358084721526	1.46641719014820
C	-2.51362008899457	2.45430981177522	1.50795209097656
H	-3.30059156011242	3.13075941658205	1.78439647961613
C	2.00204992875891	3.79437939994257	3.47082760365923
H	2.93562347364664	3.29236876374130	3.64375059790632
C	1.31425142404876	4.70834817965456	4.27088175314963
H	1.61178587394307	5.08450800982605	5.23066452096644
C	0.17321046140918	5.02675293987747	3.57704149262052
H	-0.64958228548309	5.68047254097149	3.79798149147421
C	1.04077079886754	4.59393522738774	-1.60600153698043
H	1.83588724281651	4.22586581748015	-2.22751288747400
C	0.13012993866641	5.62637144117606	-1.83428754484955
H	0.06438187822459	6.25760329467343	-2.69947469798215
C	-0.66837255097628	5.67180895282499	-0.71642379630217
H	-1.50179630492503	6.29378803838500	-0.44805517696092
P	3.72172036997840	3.39021441586509	0.43686504048235
H	4.65777108252028	3.15677586892495	1.45925020216316
H	4.45274114033978	3.08439127090388	-0.72505853345136
H	3.65232670057381	4.79176831389926	0.40309349867648
H	0.31099118654277	-0.95730142467025	-4.31984417319990
C	-0.64733623338838	-0.62295550532181	-3.94658523483926
C	-3.10716331231985	0.23038619638604	-2.99471246936560
C	-1.00935817548596	-0.91132739354962	-2.63237389772160
C	-1.50013772401879	0.09137853953943	-4.77431882390764
C	-2.73320836338141	0.52253842340116	-4.29917394860146
C	-2.24964881346163	-0.48493753981923	-2.16953784462612
H	-1.20657780685432	0.30724489469969	-5.79175656317063
H	-3.39924660430135	1.07755808554811	-4.94410959646440
H	-2.54879887241461	-0.71614493066784	-1.15752983018572
H	-4.06800733275809	0.55369771503374	-2.62019782901677
C	-0.06767949666349	-1.65061342699357	-1.73346828064801
H	0.41959660205744	-2.46803863782714	-2.26637914135366
H	-0.60190217218558	-2.10928575776436	-0.90042362561822
N	1.03235715167843	-0.88392296544754	-1.12226580586436
N	1.03720339956760	0.32897146900094	-1.39167727230575
H	2.66425451568952	-1.89141806406016	1.58851082067083
C	2.12593049038828	-1.53404689702116	2.45261287405549
C	0.69271474291100	-0.56447189482920	4.64724220534816
C	1.71968904885608	-0.18146518993188	2.49808766386532
C	1.82573271713304	-2.37551109200619	3.49967406167334
C	1.10983327101794	-1.89327514718004	4.59388368127848
C	0.99195543182581	0.28999745930975	3.61083621806960
H	2.13723566746770	-3.40849326419647	3.47110649996670
H	0.87013256794014	-2.56002164761637	5.40921584789491
H	0.66947994353874	1.31731145156377	3.63275257601773
H	0.13358049077310	-0.20825059164441	5.49878092851097
C	2.02895679872959	0.66616673030976	1.43527102876546
H	3.84413631409476	0.64019126233550	0.23243767072663

62

TS2

C	-0.19109941905905	-0.00522717056987	0.45819608049363
N	-0.44859904470325	0.25895882775595	2.70480567904619
C	-0.31831391571394	-1.32832904787118	0.83494979431180
N	-1.27997953266089	-1.57989042264556	1.83600646659223

Ru	-2.07724830445433	-0.58978801748578	0.01116468180328
N	-2.84423829733839	1.32953378426852	0.66917657749070
N	-3.92462359041650	1.80893793782394	0.00932250416999
N	-2.32966453197491	0.29522538673445	-1.83920089078046
N	-3.50004315000050	0.90330129302143	-2.12649918547935
N	-4.14015363402613	-1.06006850436971	-0.06662213207474
N	-4.99313177418615	-0.19365363758605	-0.64251358619535
C	-4.48910869459663	1.07415643646213	-1.09248645386214
H	-5.31148696461066	1.64592695118171	-1.50178621948214
C	-2.45426316048835	2.31505610997186	1.46728304076206
H	-1.58732686208514	2.19543808462048	2.07700080000952
C	-3.28900678104596	3.42575866253907	1.33630917919487
H	-3.22050687338398	4.35582708816379	1.86660837764652
C	-4.21386953345600	3.07623896584293	0.38702684188544
H	-5.04357296251833	3.60174856584961	-0.04769558611667
C	-1.59240445750466	0.36861730186412	-2.94495523828466
H	-0.60691129810427	-0.05744154706879	-2.95800298558792
C	-2.29383075389334	1.04274882473072	-3.94617951648151
H	-1.95719451821723	1.25739890515810	-4.94205788061752
C	-3.50502924335886	1.37822391347332	-3.39380021014496
H	-4.35714107014072	1.90532784514581	-3.78028896664454
C	-4.88384661834495	-2.07020332756280	0.37927609674007
H	-4.41680957521286	-2.90301351038000	0.87162829919997
C	-6.23074476265491	-1.83731141572001	0.09483898372457
H	-7.06371439250017	-2.47242000101073	0.32737945522291
C	-6.27180609567669	-0.62394375670374	-0.54851496280615
H	-7.08174413185226	-0.03824216166258	-0.94132075671933
P	-1.81678739037039	-2.55712543603303	-1.15257464212773
H	-0.67646483789904	-2.66209744889599	-1.96764899517850
H	-1.76950364480128	-3.74808937518732	-0.40600572007318
H	-2.86316435784556	-2.79145803591781	-2.05807200703611
H	-0.71704931791669	-0.58908780454405	5.78731977975097
C	-1.32991579633716	0.29964434410273	5.83146604046270
C	-2.88640086911349	2.59260364308200	5.96433647804084
C	-1.14721045880941	1.31000437589482	4.88989085518135
C	-2.28914771957395	0.42989310229500	6.82365181888083
C	-3.07290525716672	1.57567916934670	6.88995877148765
C	-1.92698921495861	2.45945625869681	4.96980734405074
H	-2.42131466190565	-0.35953039585966	7.54940221171851
H	-3.81901781579650	1.67747809389189	7.66474671750068
H	-1.77646151956485	3.26205089081330	4.26234998123166
H	-3.48239395383957	3.49219964708234	6.01767951268451
C	-0.11698625388161	1.16657425971433	3.81102910111367
H	0.81999097825760	0.77727816327524	4.21380679081253
H	0.12462202737594	2.12351104776101	3.35455241632500
N	-1.24841322792484	-0.65976062121026	2.85529083298747
H	2.40682482137309	-0.15409372572096	0.97662099964634
C	2.14032793808791	0.70880424006435	0.38518880269058
C	1.40847527055809	2.94621219619609	-1.10835152104659
C	0.79093873625264	0.92179362313993	0.04942569973565
C	3.10503180207787	1.59641272298321	-0.04222794009140
C	2.74137156776724	2.71642851098511	-0.78694497730434
C	0.43570271866134	2.05763282706506	-0.69370489552559
H	4.14193991379326	1.42680946228089	0.20667598966552
H	3.50027401558978	3.41270063617663	-1.11253356027263

H	-0.60055581889166	2.22909789470022	-0.93352025192424
H	1.13219427304264	3.81887749014142	-1.68071511948270
H	0.24099199794004	-2.16479212029127	0.43796119907934

62
INT2

C	-0.29055669859773	0.47059759765411	1.43892615012035
N	-0.96682749354933	0.85104120954125	2.53366662300911
C	-0.74629205049151	-0.82783738071211	1.18603047568092
N	-1.70357254561350	-1.11753036654752	2.14030407466870
Ru	-2.84241082085659	-0.99795547582560	0.21034317903771
N	-3.68787667404508	0.90368351358053	0.70361817667635
N	-4.21143146156059	1.60359505050565	-0.32815602730019
N	-2.15109958563244	-0.16418883981521	-1.44820060955282
N	-2.92109206329889	0.72839774789963	-2.10808594640659
N	-4.59795919523235	-1.22717593747298	-0.78910939533162
N	-5.01485517171395	-0.22176377205903	-1.58962746073888
C	-4.24819887290381	0.99720599334863	-1.63379854618876
H	-4.72671502139171	1.68169372631264	-2.32201985407081
C	-4.04439235246814	1.56989596169351	1.80093340329030
H	-3.75393607300924	1.20123110693352	2.76503641229605
C	-4.78894205451539	2.70478878029194	1.46917521317177
H	-5.19276507625043	3.43538470678703	2.14338300313070
C	-4.89501722968569	2.69107914628457	0.10201266799679
H	-5.38359640791914	3.34536384104334	-0.59566451936102
C	-0.98018813773180	-0.19086343253630	-2.08504673276713
H	-0.19129291430360	-0.83387302793471	-1.74828149656159
C	-1.00239061520268	0.70283096275474	-3.15607707413951
H	-0.20543310004146	0.89898948275958	-3.84700131143651
C	-2.24942190867482	1.27573721014153	-3.14744445629914
H	-2.71677732657503	2.00993914242463	-3.77631716739729
C	-5.54401838944799	-2.16384634089486	-0.84848170465771
H	-5.43599103430123	-3.07603488844157	-0.29319990046669
C	-6.58061010705575	-1.74501574597151	-1.68497959751797
H	-7.47537069552731	-2.28549502012060	-1.92692413095675
C	-6.21915161294763	-0.50053990537499	-2.13885788365021
H	-6.70106926874165	0.20092022911379	-2.79371214239165
P	-2.19293787806362	-3.09061529462142	-0.45271277513922
H	-1.21969214808536	-3.77296568023655	0.30628050584711
H	-3.19149782705989	-4.08182939586310	-0.52683433958878
H	-1.63485423278647	-3.20130995631129	-1.74336166198002
H	1.01800772845891	0.98774631000986	4.86759539377698
C	0.14189145208304	1.29038813316577	5.42431439346946
C	-2.10910612551110	2.07317185675385	6.84773698653181
C	-0.92482854236764	1.88768034290548	4.75861114073483
C	0.07974132366211	1.07922633599522	6.79265459383918
C	-1.04611101249608	1.47193063844513	7.50722519398765
C	-2.04872145169365	2.27827213200130	5.47601598429321
H	0.91078956081707	0.61494242736527	7.30348352045944
H	-1.09272640894470	1.31002796959765	8.57437973581392
H	-2.87772823434759	2.74016009034203	4.95873849898552
H	-2.98555087174452	2.38113313148526	7.39916322993194
C	-0.85695391653426	2.11129020616134	3.28021384144091
H	0.09421973058087	2.54480630857992	2.98910842191331

H	-1.65090395196404	2.77266905917547	2.94554174346379
N	-1.82810413829568	-0.09820326912258	2.95080853369247
H	2.06188935311787	-0.33771905334844	0.48503911459384
C	1.82563835122429	0.67949141033912	0.21108520014715
C	1.20718113904025	3.29585873193757	-0.49243790750823
C	0.64577219442640	1.26586950063304	0.67365656652950
C	2.69251479521994	1.40582050913694	-0.58645162745697
C	2.38648048246294	2.71441167603101	-0.93873695120234
C	0.33697406723673	2.57837172938696	0.31073794592262
H	3.60767820785991	0.95006968907043	-0.93413672632560
H	3.06277237607785	3.27713358998720	-1.56522418405484
H	-0.59689302058738	3.02044829056988	0.62172640559878
H	0.95876039782411	4.30727248626915	-0.77769970236319
H	-0.19385944032245	-1.58180518120568	0.65894550275916

PATH 3'g_b

62
TS1

N	1.41982402755210	0.62037846417722	0.89648996472606
C	0.81620671599830	-0.06720416846278	2.35247768163706
Ru	-0.03927183534663	1.91552882962445	1.55774610339457
N	-1.50112669864243	1.29448767340399	0.09723013698207
N	-2.37342686709681	2.23156763478361	-0.32776948007484
N	-1.62711323544538	2.93733771813768	2.39817657575901
N	-2.45786161670072	3.62535093137788	1.58522542265918
N	0.12817493379369	3.56539049596961	0.25605382219055
N	-0.99447523242256	4.14544416233298	-0.20697522111348
C	-2.26805816345130	3.58466692597687	0.15786568792521
H	-3.04935536038739	4.17428480049237	-0.30432711978381
C	-1.91118230311506	0.14846304636125	-0.43596099846966
H	-1.37113971874662	-0.75935153888076	-0.24574411472430
C	-3.05162836923895	0.35299161439242	-1.21514892835012
H	-3.59321024676150	-0.38132735646035	-1.77950040782723
C	-3.32631946892335	1.69388905737163	-1.12385934758703
H	-4.09749996032660	2.30755878067036	-1.55053407963859
C	-2.10097124171026	3.09808259771803	3.63015699405642
H	-1.59835603279271	2.63788332372313	4.46028512325133
C	-3.24796007267718	3.89580862184977	3.60704602146312
H	-3.84561022022302	4.19861912809823	4.44512995293037
C	-3.45506172767672	4.21340162634696	2.28812895791828
H	-4.20879487004049	4.79533718726054	1.79168697873823
C	1.14129260490327	4.18937338613957	-0.33936195947150
H	2.15222964396699	3.89955302705604	-0.12067184037226
C	0.65772869901069	5.17889516867304	-1.19804784376380
H	1.23348117831444	5.84716020350582	-1.80894242173556
C	-0.71114402455011	5.11973623527858	-1.10268053718380
H	-1.49678928479800	5.67380254890652	-1.58112506381022
P	1.41589747251859	2.91396778592084	3.04806008109295
H	2.76783478793265	2.52467497067555	3.01402285123282
H	1.51127732946909	4.30880852657748	2.90225598396814
H	1.11251169050013	2.78666413258938	4.41574989224191
H	3.06073375003010	0.84678390598550	-2.56807816937960
C	2.06466432772112	0.96722711113216	-2.97048132979300

C	-0.49072410063416	1.27685209288914	-4.00307648624026
C	1.14237345995899	-0.06934918797470	-2.84536158205433
C	1.71000927145184	2.15200131705810	-3.59867890234837
C	0.42865455302537	2.31155107678661	-4.11052616443136
C	-0.13345218345330	0.09215609252784	-3.37479395676266
H	2.43157622409982	2.95149100489727	-3.68496334386043
H	0.14997689246356	3.23680941806570	-4.59388109837369
H	-0.85397260619473	-0.70749334814561	-3.28077548191017
H	-1.48791472499155	1.39296285523499	-4.40245724651750
C	1.48027174876064	-1.28945028715625	-2.04555242160043
H	2.52551976055163	-1.57425102092179	-2.16725434412575
H	0.86717361939273	-2.13872067944360	-2.33076507998567
N	1.23877281138365	-1.10631807932024	-0.58899214566640
N	1.41433498996841	0.03703994317627	-0.17447817582859
H	3.39699347711652	-0.62801355120150	1.80272759437785
C	3.16070147888079	-0.67583407709446	2.85330428157770
C	2.53905632963238	-0.79509454843757	5.56505878528190
C	1.86161472296335	-0.39210573696690	3.28637652913307
C	4.13502385565693	-1.01924870063836	3.77246596629858
C	3.82758450844322	-1.07909656704818	5.12657892571577
C	1.55942844296666	-0.44843317169426	4.65442762326101
H	5.13602896127263	-1.24146611421638	3.43426244619252
H	4.59310357315640	-1.34363097318399	5.84116054613616
H	0.56092311062664	-0.20989241439489	4.98873965434766
H	2.30283270452041	-0.83557629117105	6.61788042207154
H	-1.38207255660567	-0.26123931711481	2.78875532717951
C	-0.47252893505058	0.19333370678205	2.43906895904341

62

INT1

C	2.75926055792247	0.65008199479058	0.23756677330464
N	2.10303486549045	1.01926610182127	-0.93142879827479
Ru	1.64533176583160	2.39182240860243	0.65514386420379
N	-0.43456302540912	1.90367986739326	0.90837555053200
N	-1.27024944258253	2.91317895912594	1.23671933853541
N	1.32233090590287	3.56204545965564	2.35028660776989
N	0.21127752063428	4.32444005436168	2.42102701780125
N	0.82066870613312	4.04176294315461	-0.41525836517754
N	-0.21752481413043	4.71145228176630	0.12096466136418
C	-0.77132935812004	4.25836546686301	1.36893537422888
H	-1.59803381249340	4.90526136737151	1.63372855650948
C	-1.16345433883407	0.79480321306249	0.97850096866517
H	-0.71943554494948	-0.15888819911214	0.77558328223475
C	-2.47646468730908	1.09311467345479	1.34489964050939
H	-3.28930225436792	0.40358084721526	1.46641719014820
C	-2.51362008899457	2.45430981177522	1.50795209097656
H	-3.30059156011242	3.13075941658205	1.78439647961613
C	2.00204992875891	3.79437939994257	3.47082760365923
H	2.93562347364664	3.29236876374130	3.64375059790632
C	1.31425142404876	4.70834817965456	4.27088175314963
H	1.61178587394307	5.08450800982605	5.23066452096644
C	0.17321046140918	5.02675293987747	3.57704149262052
H	-0.64958228548309	5.68047254097149	3.79798149147421
C	1.04077079886754	4.59393522738774	-1.60600153698043

H	1.83588724281651	4.22586581748015	-2.22751288747400
C	0.13012993866641	5.62637144117606	-1.83428754484955
H	0.06438187822459	6.25760329467343	-2.69947469798215
C	-0.66837255097628	5.67180895282499	-0.71642379630217
H	-1.50179630492503	6.29378803838500	-0.44805517696092
P	3.72172036997840	3.39021441586509	0.43686504048235
H	4.65777108252028	3.15677586892495	1.45925020216316
H	4.45274114033978	3.08439127090388	-0.72505853345136
H	3.65232670057381	4.79176831389926	0.40309349867648
H	0.31099118654277	-0.95730142467025	-4.31984417319990
C	-0.64733623338838	-0.62295550532181	-3.94658523483926
C	-3.10716331231985	0.23038619638604	-2.99471246936560
C	-1.00935817548596	-0.91132739354962	-2.63237389772160
C	-1.50013772401879	0.09137853953943	-4.77431882390764
C	-2.73320836338141	0.52253842340116	-4.29917394860146
C	-2.24964881346163	-0.48493753981923	-2.16953784462612
H	-1.20657780685432	0.30724489469969	-5.79175656317063
H	-3.39924660430135	1.07755808554811	-4.94410959646440
H	-2.54879887241461	-0.71614493066784	-1.15752983018572
H	-4.06800733275809	0.55369771503374	-2.62019782901677
C	-0.06767949666349	-1.65061342699357	-1.73346828064801
H	0.41959660205744	-2.46803863782714	-2.26637914135366
H	-0.60190217218558	-2.10928575776436	-0.90042362561822
N	1.03235715167843	-0.88392296544754	-1.12226580586436
N	1.03720339956760	0.32897146900094	-1.39167727230575
H	2.66425451568952	-1.89141806406016	1.58851082067083
C	2.12593049038828	-1.53404689702116	2.45261287405549
C	0.69271474291100	-0.56447189482920	4.64724220534816
C	1.71968904885608	-0.18146518993188	2.49808766386532
C	1.82573271713304	-2.37551109200619	3.49967406167334
C	1.10983327101794	-1.89327514718004	4.59388368127848
C	0.99195543182581	0.28999745930975	3.61083621806960
H	2.13723566746770	-3.40849326419647	3.47110649996670
H	0.87013256794014	-2.56002164761637	5.40921584789491
H	0.66947994353874	1.31731145156377	3.63275257601773
H	0.13358049077310	-0.20825059164441	5.49878092851097
C	2.02895679872959	0.66616673030976	1.43527102876546
H	3.84413631409476	0.64019126233550	0.23243767072663

62
TS2

C	-0.19109941905905	-0.00522717056987	0.45819608049363
N	-0.44859904470325	0.25895882775595	2.70480567904619
C	-0.31831391571394	-1.32832904787118	0.83494979431180
N	-1.27997953266089	-1.57989042264556	1.83600646659223
Ru	-2.07724830445433	-0.58978801748578	0.01116468180328
N	-2.84423829733839	1.32953378426852	0.66917657749070
N	-3.92462359041650	1.80893793782394	0.00932250416999
N	-2.32966453197491	0.29522538673445	-1.83920089078046
N	-3.50004315000050	0.90330129302143	-2.12649918547935
N	-4.14015363402613	-1.06006850436971	-0.06662213207474
N	-4.99313177418615	-0.19365363758605	-0.64251358619535
C	-4.48910869459663	1.07415643646213	-1.09248645386214
H	-5.31148696461066	1.64592695118171	-1.50178621948214

C	-2.45426316048835	2.31505610997186	1.46728304076206
H	-1.58732686208514	2.19543808462048	2.07700080000952
C	-3.28900678104596	3.42575866253907	1.33630917919487
H	-3.22050687338398	4.35582708816379	1.86660837764652
C	-4.21386953345600	3.07623896584293	0.38702684188544
H	-5.04357296251833	3.60174856584961	-0.04769558611667
C	-1.59240445750466	0.36861730186412	-2.94495523828466
H	-0.60691129810427	-0.05744154706879	-2.95800298558792
C	-2.29383075389334	1.04274882473072	-3.94617951648151
H	-1.95719451821723	1.25739890515810	-4.94205788061752
C	-3.50502924335886	1.37822391347332	-3.39380021014496
H	-4.35714107014072	1.90532784514581	-3.78028896664454
C	-4.88384661834495	-2.07020332756280	0.37927609674007
H	-4.41680957521286	-2.90301351038000	0.87162829919997
C	-6.23074476265491	-1.83731141572001	0.09483898372457
H	-7.06371439250017	-2.47242000101073	0.32737945522291
C	-6.27180609567669	-0.62394375670374	-0.54851496280615
H	-7.08174413185226	-0.03824216166258	-0.94132075671933
P	-1.81678739037039	-2.55712543603303	-1.15257464212773
H	-0.67646483789904	-2.66209744889599	-1.96764899517850
H	-1.76950364480128	-3.74808937518732	-0.40600572007318
H	-2.86316435784556	-2.79145803591781	-2.05807200703611
H	-0.71704931791669	-0.58908780454405	5.78731977975097
C	-1.32991579633716	0.29964434410273	5.83146604046270
C	-2.88640086911349	2.59260364308200	5.96433647804084
C	-1.14721045880941	1.31000437589482	4.88989085518135
C	-2.28914771957395	0.42989310229500	6.82365181888083
C	-3.07290525716672	1.57567916934670	6.88995877148765
C	-1.92698921495861	2.45945625869681	4.96980734405074
H	-2.42131466190565	-0.35953039585966	7.54940221171851
H	-3.81901781579650	1.67747809389189	7.66474671750068
H	-1.77646151956485	3.26205089081330	4.26234998123166
H	-3.48239395383957	3.49219964708234	6.01767951268451
C	-0.11698625388161	1.16657425971433	3.81102910111367
H	0.81999097825760	0.77727816327524	4.21380679081253
H	0.12462202737594	2.12351104776101	3.35455241632500
N	-1.24841322792484	-0.65976062121026	2.85529083298747
H	2.40682482137309	-0.15409372572096	0.97662099964634
C	2.14032793808791	0.70880424006435	0.38518880269058
C	1.40847527055809	2.94621219619609	-1.10835152104659
C	0.79093873625264	0.92179362313993	0.04942569973565
C	3.10503180207787	1.59641272298321	-0.04222794009140
C	2.74137156776724	2.71642851098511	-0.78694497730434
C	0.43570271866134	2.05763282706506	-0.69370489552559
H	4.14193991379326	1.42680946228089	0.20667598966552
H	3.50027401558978	3.41270063617663	-1.11253356027263
H	-0.60055581889166	2.22909789470022	-0.93352025192424
H	1.13219427304264	3.81887749014142	-1.68071511948270
H	0.24099199794004	-2.16479212029127	0.43796119907934

62
INT2

C	-0.29055669859773	0.47059759765411	1.43892615012035
N	-0.96682749354933	0.85104120954125	2.53366662300911

C	-0.74629205049151	-0.82783738071211	1.18603047568092
N	-1.70357254561350	-1.11753036654752	2.14030407466870
Ru	-2.84241082085659	-0.99795547582560	0.21034317903771
N	-3.68787667404508	0.90368351358053	0.70361817667635
N	-4.21143146156059	1.60359505050565	-0.32815602730019
N	-2.15109958563244	-0.16418883981521	-1.44820060955282
N	-2.92109206329889	0.72839774789963	-2.10808594640659
N	-4.59795919523235	-1.22717593747298	-0.78910939533162
N	-5.01485517171395	-0.22176377205903	-1.58962746073888
C	-4.24819887290381	0.99720599334863	-1.63379854618876
H	-4.72671502139171	1.68169372631264	-2.32201985407081
C	-4.04439235246814	1.56989596169351	1.80093340329030
H	-3.75393607300924	1.20123110693352	2.76503641229605
C	-4.78894205451539	2.70478878029194	1.46917521317177
H	-5.19276507625043	3.43538470678703	2.14338300313070
C	-4.89501722968569	2.69107914628457	0.10201266799679
H	-5.38359640791914	3.34536384104334	-0.59566451936102
C	-0.98018813773180	-0.19086343253630	-2.08504673276713
H	-0.19129291430360	-0.83387302793471	-1.74828149656159
C	-1.00239061520268	0.70283096275474	-3.15607707413951
H	-0.20543310004146	0.89898948275958	-3.84700131143651
C	-2.24942190867482	1.27573721014153	-3.14744445629914
H	-2.71677732657503	2.00993914242463	-3.77631716739729
C	-5.54401838944799	-2.16384634089486	-0.84848170465771
H	-5.43599103430123	-3.07603488844157	-0.29319990046669
C	-6.58061010705575	-1.74501574597151	-1.68497959751797
H	-7.47537069552731	-2.28549502012060	-1.92692413095675
C	-6.21915161294763	-0.50053990537499	-2.13885788365021
H	-6.70106926874165	0.20092022911379	-2.79371214239165
P	-2.19293787806362	-3.09061529462142	-0.45271277513922
H	-1.21969214808536	-3.77296568023655	0.30628050584711
H	-3.19149782705989	-4.08182939586310	-0.52683433958878
H	-1.63485423278647	-3.20130995631129	-1.74336166198002
H	1.01800772845891	0.98774631000986	4.86759539377698
C	0.14189145208304	1.29038813316577	5.42431439346946
C	-2.10910612551110	2.07317185675385	6.84773698653181
C	-0.92482854236764	1.88768034290548	4.75861114073483
C	0.07974132366211	1.07922633599522	6.79265459383918
C	-1.04611101249608	1.47193063844513	7.50722519398765
C	-2.04872145169365	2.27827213200130	5.47601598429321
H	0.91078956081707	0.61494242736527	7.30348352045944
H	-1.09272640894470	1.31002796959765	8.57437973581392
H	-2.87772823434759	2.74016009034203	4.95873849898552
H	-2.98555087174452	2.38113313148526	7.39916322993194
C	-0.85695391653426	2.11129020616134	3.28021384144091
H	0.09421973058087	2.54480630857992	2.98910842191331
H	-1.65090395196404	2.77266905917547	2.94554174346379
N	-1.82810413829568	-0.09820326912258	2.95080853369247
H	2.06188935311787	-0.33771905334844	0.48503911459384
C	1.82563835122429	0.67949141033912	0.21108520014715
C	1.20718113904025	3.29585873193757	-0.49243790750823
C	0.64577219442640	1.26586950063304	0.67365656652950
C	2.69251479521994	1.40582050913694	-0.58645162745697
C	2.38648048246294	2.71441167603101	-0.93873695120234
C	0.33697406723673	2.57837172938696	0.31073794592262

H	3.60767820785991	0.95006968907043	-0.93413672632560
H	3.06277237607785	3.27713358998720	-1.56522418405484
H	-0.59689302058738	3.02044829056988	0.62172640559878
H	0.95876039782411	4.30727248626915	-0.77769970236319
H	-0.19385944032245	-1.58180518120568	0.65894550275916

22

[CpRuCl(NCCMe)(PH_3)]

C	-0.33964476011385	0.46139029131008	-0.13158721415920
H	-1.36350998659406	0.18613335957632	0.05153077376193
C	0.66611929985233	-0.40253313931055	-0.64650893872279
H	0.53084911704768	-1.41730654796565	-0.97348661132255
C	1.89169919460935	0.33891021559642	-0.64309901912827
H	2.85268969789046	-0.02870572331329	-0.95774917277690
C	1.62632205932340	1.65261013439730	-0.16600945360164
H	2.34082639250143	2.45255650251433	-0.08842208749415
C	0.22708476297353	1.73086039936276	0.15134463177107
H	-0.29861603179640	2.59541303070040	0.51391337961732
P	1.87776149684853	1.63592772098955	3.06787950951360
H	3.14091838103070	1.45279113158807	3.68487142302992
H	2.00573236080543	3.00807113895188	2.75637031553900
H	1.10767602741422	1.76232690536020	4.25173867790036
N	2.57284401482445	-1.11431292453984	1.98779765994856
C	3.36562997353324	-1.89439526111129	2.30382752130194
C	4.35595164361723	-2.86701457406759	2.68075896000570
H	4.17246383831978	-3.80865079095736	2.16657177065608
H	4.31872665604887	-3.04296544940318	3.75400373772889
H	5.35147535023905	-2.51316361113149	2.41873501727871
Ru	1.21461957601365	0.23652657337307	1.40151397570517
Cl	-0.37870906438912	-0.85339938192013	2.98967514344721

6. Reactivity of complex [Ru(κ^3-Tpm)(PPh_3)($NCMe)_2$]$(NO_3)_2$ (3) along cycloaddition of phenyl azide and phenylacetylene in dimethylformamide

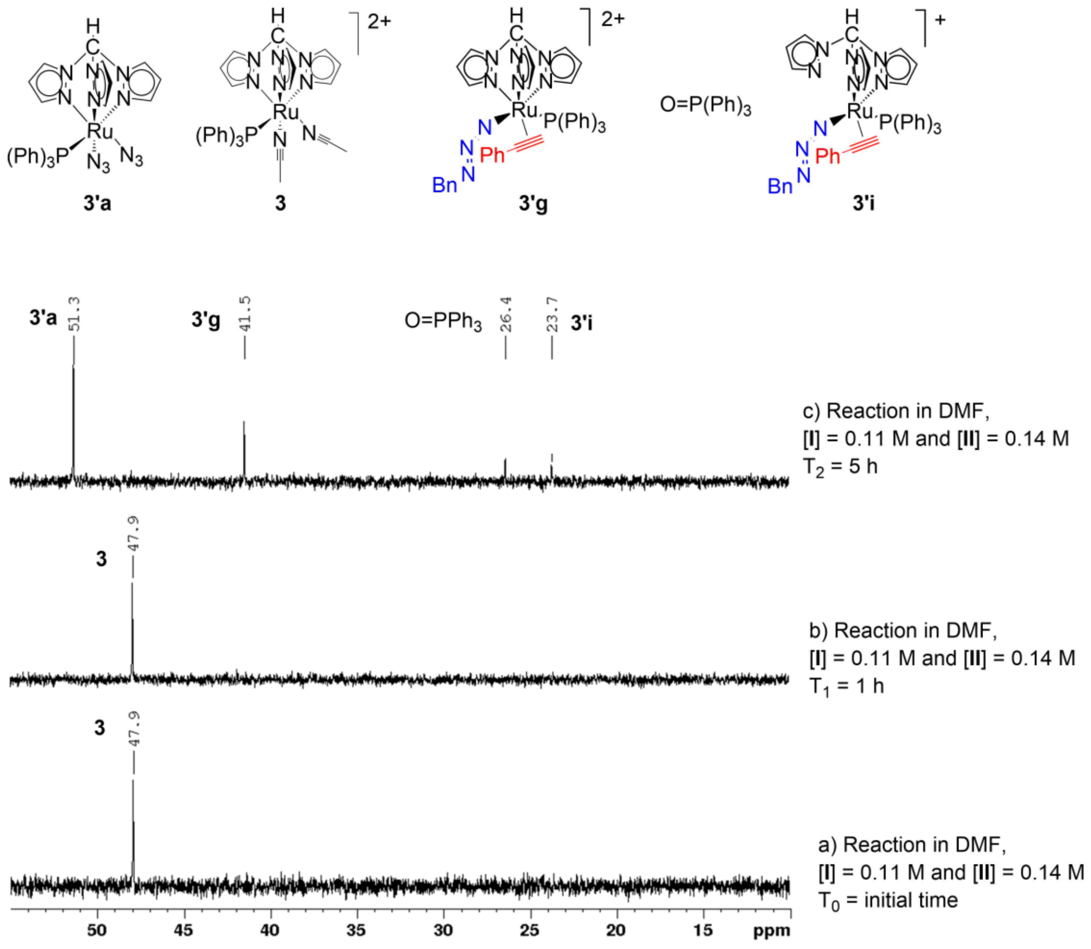

Figure S25. Proposed assignation for the species in solution **3'a**, **3**, **3'g**, O=P(Ph)$_3$ and **3'i** of the reaction of **3** and phenyl azide (**I**) and phenylacetylene (**II**) in DMF vs time (a, b and c) in the ^{31}P {^{1}H} NMR (121.49 MHz, 25°C, D_2O capillary). Conditions: 1 mol% **3**, 0.6 mL of the reaction in DMF, **I** was formed *in situ* by reaction of **I'** and **I''** in a ratio 1:1, **I**/**II** = 1:1.25, [**I**] = 0.55 M and [**II**] = 0.7 M.

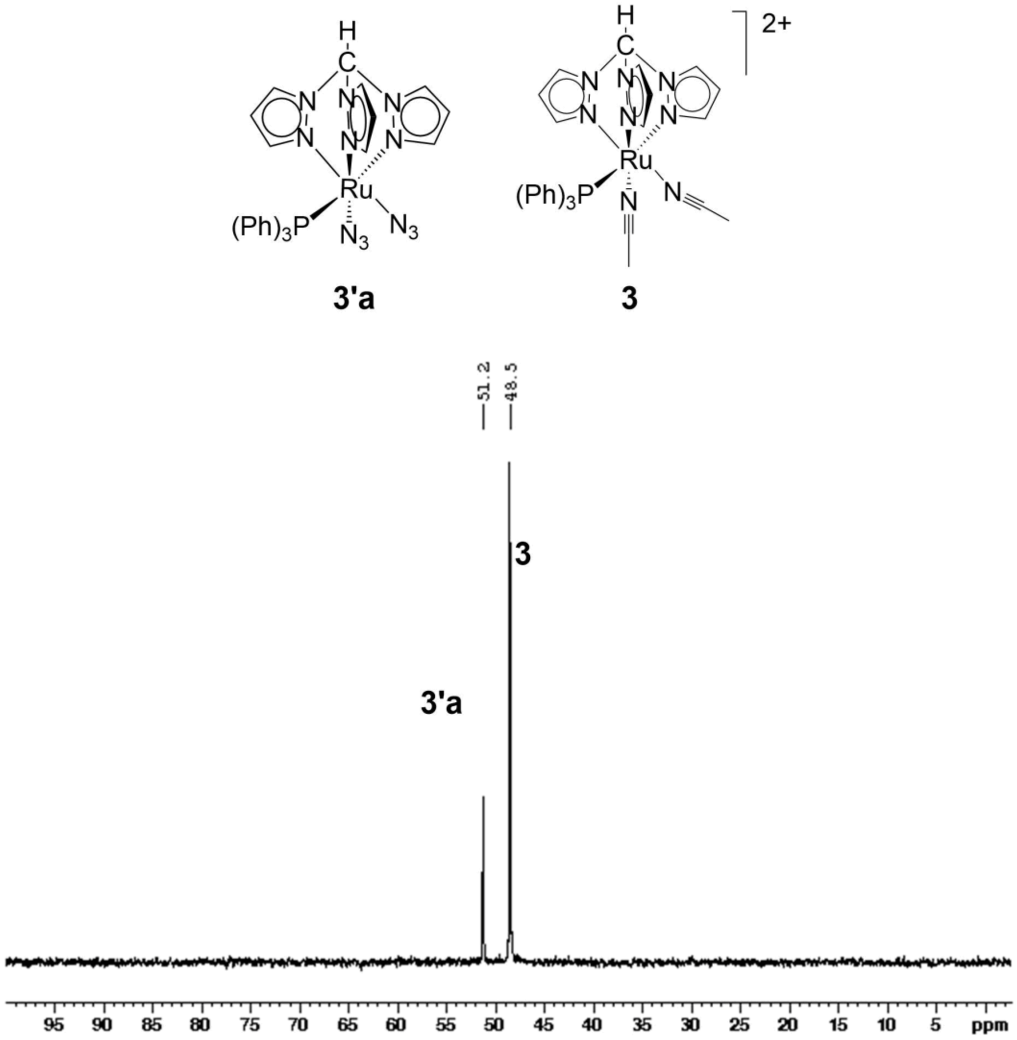

Figure S26. Proposed assignation for the species (**3’** and **3**) in solution of the reaction of **3** and sodium azide (**I’**) in DMF-d7 in the ^{31}P {^{1}H} NMR (121.49 MHz, 25°C, DMF-d7). Conditions: 1 mol% **3**, 0.6 mL of the reaction in DMF-d7, [**I’**] = 0.55 M.

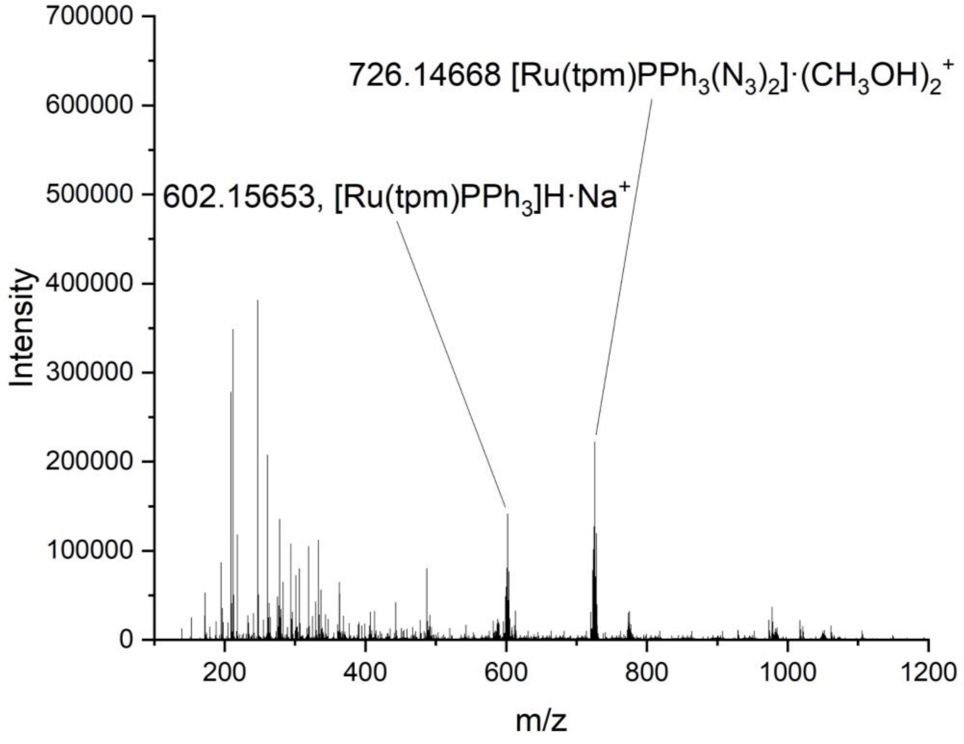

Figure S27. Mass spectrum of the reaction of complex **3** and sodium azide (**I**) in DMF at 100ºC for 1 h.

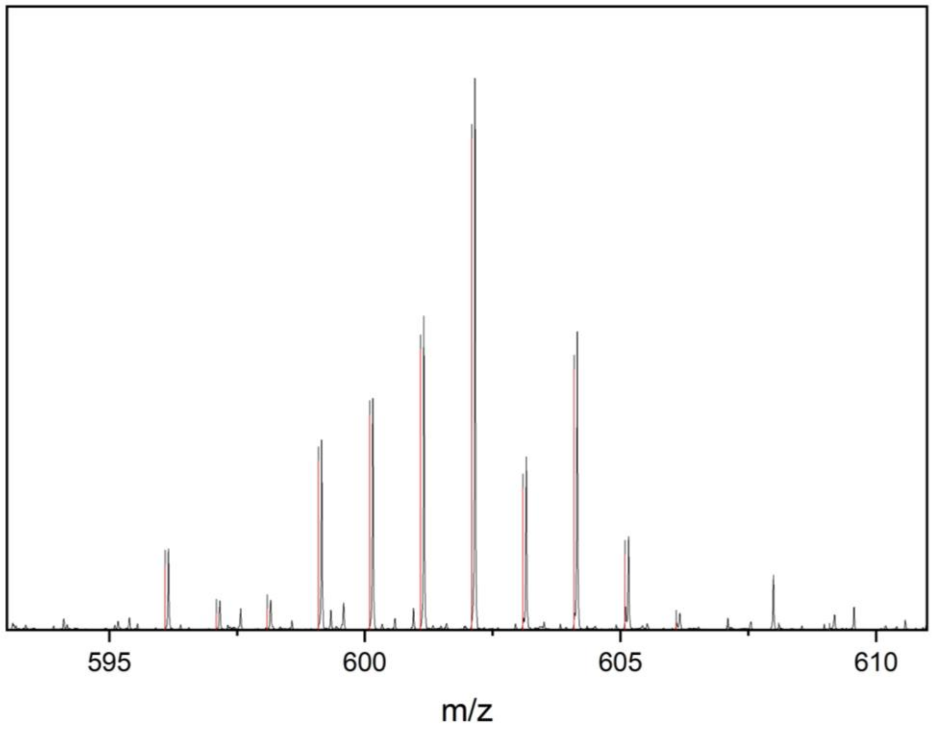

Figure S28. Ampliation of the mass spectrum of Figure S26 showing the experimental (black) and calculated (red) isotopic distribution for [Ru(tpm)(PPh_3)]H·Na.

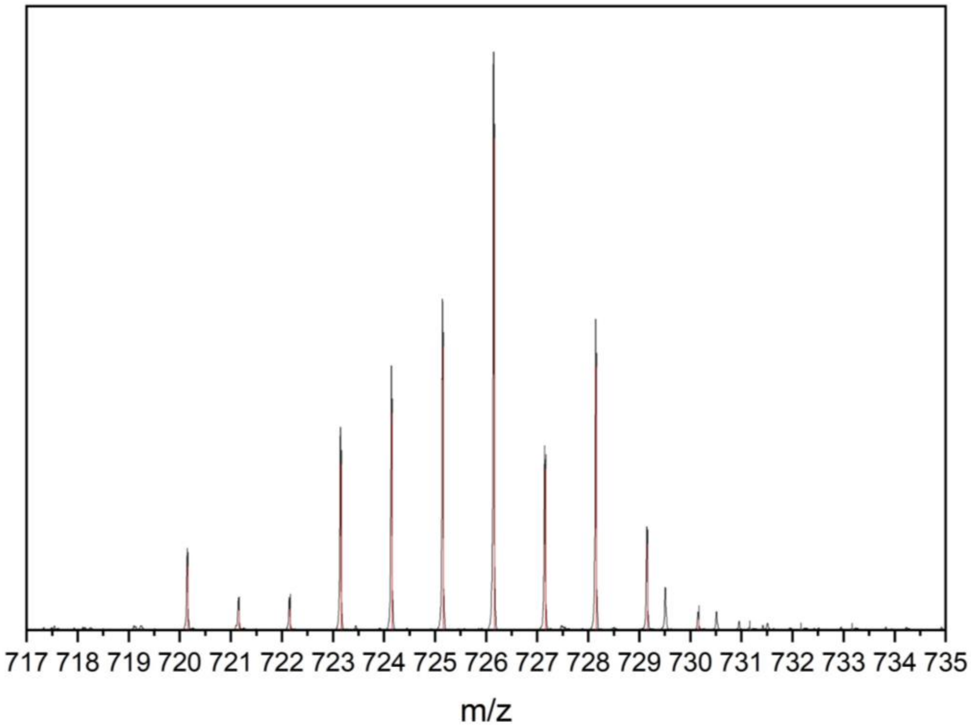

Figure S29. Ampliation of the mass spectrum of Figure S26 showing the experimental (black) and calculated (red) isotopic distribution for $[Ru(tpm)(PPh_3)(N_3)_2]\cdot(CH_3OH)_2$.

7. References

(1) A. Gobbo, J. Vančo, S. Benetti, T. Malina, Z. Dvořák, C. Castelli, A. Chiappa, M. Guelfi, S. Zacchini, T. Biver, Z. Trávníček, F. Marchetti, *Inorg. Chem.* **2025**, *64*, 24615–24633.
